# Re-generation of cytotoxic γδT cells with distinctive signatures from human γδT-derived iPSCs

**DOI:** 10.1016/j.stemcr.2023.02.010

**Published:** 2023-03-23

**Authors:** Nobuyuki Murai, Michiyo Koyanagi-Aoi, Hiroto Terashi, Takashi Aoi

**Affiliations:** 1 Division of Stem Cell Medicine, Graduate School of Medicine, Kobe University, Kobe, Hyogo, Japan; 2 Division of Advanced Medical Science, Graduate School of Science, Technology and Innovation, Kobe University, Kobe, Hyogo, Japan; 3 Division of Plastic Surgery, Graduate School of Medicine, Kobe University, Kobe, Hyogo, Japan; 4 Center for Human Resource Development for Regenerative Medicine, Kobe University Hospital, Kobe, Hyogo, Japan

**Keywords:** γδT cell, iPSC, immunotherapy, cytotoxicity, single-cell RNA-seq, HLA, MHC, TCR, T cell repertoire, granzyme, perforin

## Abstract

For a long time, *ex vivo*-expanded peripheral-blood-derived γδT cell (PBγδT)-based immunotherapy has been attractive, and clinical trials have been undertaken. However, the difficulty in expanding cytotoxic γδT cells to an adequate number has been a major limitation to the efficacy of treatment in most cases. We successfully re-generated γδT cells from γδT cell-derived human induced pluripotent stem cells (iPSCs). The iPSC-derived γδT cells (iγδTs) killed several cancer types in a major histocompatibility complex (MHC)-unrestricted manner. Single-cell RNA sequencing (scRNA-seq) revealed that the iγδTs were identical to a minor subset of PBγδTs. Compared with a major subset of PBγδTs, the iγδTs showed a distinctive gene expression pattern: lower *CD2*, *CD5*, and antigen-presenting genes; higher *CD7*, *KIT*, and natural killer (NK) cell markers. The iγδTs expressed granzyme B and perforin but not interferon gamma (IFNγ). Our data provide a new source for γδT cell-based immunotherapy without quantitative limitation.

## Highlights

• We successfully re-generated γδT cells from γδT cell-derived human iPSCs
• The re-generated γδT cells showed cytotoxicity against cancer cell lines
• Distinctive signatures of the re-generated γδT cells were identified

## Graphical abstract

**Figure:**
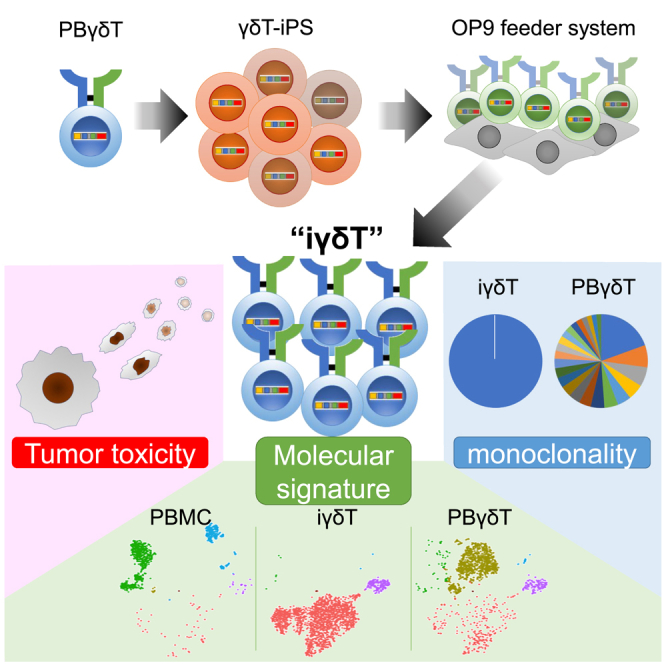

## Teaser

Murai et al. successfully re-generated γδT cells from γδT cell-derived human iPS cells. Molecular signatures of the re-generated γδT cells (iγδTs) were identical to those of a minor subset of *ex vivo*-expanded peripheral-blood-derived γδT cells. Cytotoxicity of iγδTs against several cancer cell lines was exhibited in an MHC-unrestricted manner. The novel cell resource will advance off-the-shelf γδT cell-based immunotherapy.

## Introduction

Gamma delta T (γδT) cells attack various types of cancer cells in a major histocompatibility complex (MHC)-unrestricted manner (Wrobel et al., 2007). Therefore, peripheral blood-derived γδT cell (PBγδT)-based immunotherapy has received attention, and clinical trials have been undertaken (Kobayashi et al., 2010). Because the proportion of γδT cells in adult peripheral blood mononuclear cells amounts to only a few percent or less (Aljurf et al., 2002), γδT cells need to be expanded by stimulants *ex vivo* for clinical use (Khan et al., 2021). However, in most cases, the difficulty in expanding cytotoxic γδT cells derived from peripheral blood to an adequate number has been a major limitation to the efficacy of the treatment (Wada et al., 2014). Furthermore, PBMC-derived γδT cells can be expanded enough for them to be used for autologous adoptive immunotherapy, but not enough for them to be used for allogenic mass-produced immunotherapeutic modalities.

Induced pluripotent stem cell (iPSC) technology may be able to overcome these limitations and enable us to realize off-the-self allogenic γδT cell-based therapy, which has several advantages over autologous γδT cells therapy: (1) the *ex vivo* expansion rate of PBMC-derived γδT cells varies widely from donor individual to individual; thus, *ex vivo*-expanded autologous PBγδT cell therapy cannot be applied to all patients; (2) patients have to wait for the expansion of autologous cells but not for off-the shelf allogenic cells; and (3) an autologous approach would be associated with high costs. iPSCs have infinite proliferation ability: they show logarithmic growth for at least 100 days and ∼1,028-fold expansion during 100 days (∼10,000-fold/2 weeks) (Nakagawa et al., 2014). To generate γδT cells from human iPSCs (hiPSCs), the favorable cell of origin of the iPSC is a γδT cell, because T cell receptor (TCR) gene rearrangement is theoretically retained throughout the process of reprogramming and differentiation. Indeed, αβT cells differentiated from αβT cell-derived hiPSCs reportedly retained parental αβTCR gene rearrangement (Nishimura et al., 2013; Vizcardo et al., 2013), and re-generated αβT cells from iPSCs showed an antigen-specific cytotoxicity to cancer cells (Nishimura et al., 2013).

Previously we successfully established hiPSC lines from peripheral blood-derived γδT cells with a simple and clinically applicable method (Watanabe et al., 2018). These γδT cell-derived iPSCs (γδT-iPSCs) were demonstrated to be able to differentiate into CD34(+)CD43(+) hematopoietic progenitor cells. However, it has not yet been clarified whether the γδT-iPSCs can differentiate into γδT cells that can kill various types of cancer in an MHC-unrestricted manner. In a previous report by Zeng et al. (2019), the authors reported the generation of “mimetic γδT cells” endowed with natural killer (NK) receptors from γδT cell-derived iPSCs and designated them as γδNKT cells. However, the cells do not match any type of physiologically existing, authentic, or *bona fide* lymphocyte, including γδT cells, and should be categorized as cells resulting from an aberrant characteristic of lymphocytes derived from iPSCs, or abnormal cells. Previous studies have demonstrated that such abnormal cells can be derived from pre-rearranged TCR-carrying pluripotent stem cells or hematopoietic progenitors, and such iPSCs reportedly gave rise to abnormal T cells expressing TCR, an NK cell marker NK1.1, and CD8αα (Vizcardo et al., 2018). Similarly, the resultant cells generated by Zeng et al. also express TCR, NK cell molecules, and CD8αα, suggesting the possibility that the cells might be mere "abnormal T cells" that had previously been known to be derived from pre-rearranged TCR-carrying pluripotent stem cells through unphysiological processes but not cells with any potential clinical utility.

Accordingly, if the γδT-iPSCs could differentiate into γδT cells, whether the molecular signatures of the re-generated resultant γδT cells from the γδT-iPSCs are identical to some subset of authentic γδT cells or absolutely artificial and unnatural cells should be revealed.

In this study, we successfully re-generated γδT cells from γδT-iPSCs. The iPSC-derived γδT cells (iγδTs) exhibited cytotoxicity against several cancer cell lines in an MHC-unrestricted manner. We identified distinctive molecular signatures of iγδTs and clarified that the iγδTs were identical to a minor subset of *ex vivo*-expanded PBγδT cells. Our data provide a new source for γδT cell-based immunotherapy without quantitative limitations.

## Results

### Re-differentiation of γδT-iPSCs into γδT cells

For re-differentiation into γδT cells, we used two γδT-derived hiPSC lines from different donors: 62B3, which was established in our previous report (Watanabe et al., 2018), and 121-3, which was newly established in this study. We confirmed that both iPSC clones expressed undifferentiated markers (NANOG, OCT3/4, and SOX2) at protein levels (Figure S1A) and mRNA levels (Figure S1B) and that the Sendai virus vector used for the introduction of reprogramming factors had been removed (Figure S1B). An *in vitro* embryoid body (EB)-mediated differentiation experiment showed that they could differentiate into three germ layers (Figure S1C). In a Q-band analysis, karyotype abnormality was not observed (Figure S1D). Genomic PCR to examine the rearrangement at the *TCRG* and *TCRD* gene locus showed Vγ9-to-JP and Vδ2-to-JD1 recombination (Figure S1E). These data verified that the two lines (62B3 and 121-3) are γδT-derived iPSCs (γδT-iPSC) that carry Vγ9Vδ2-TCR genes.

**Figure 1:**
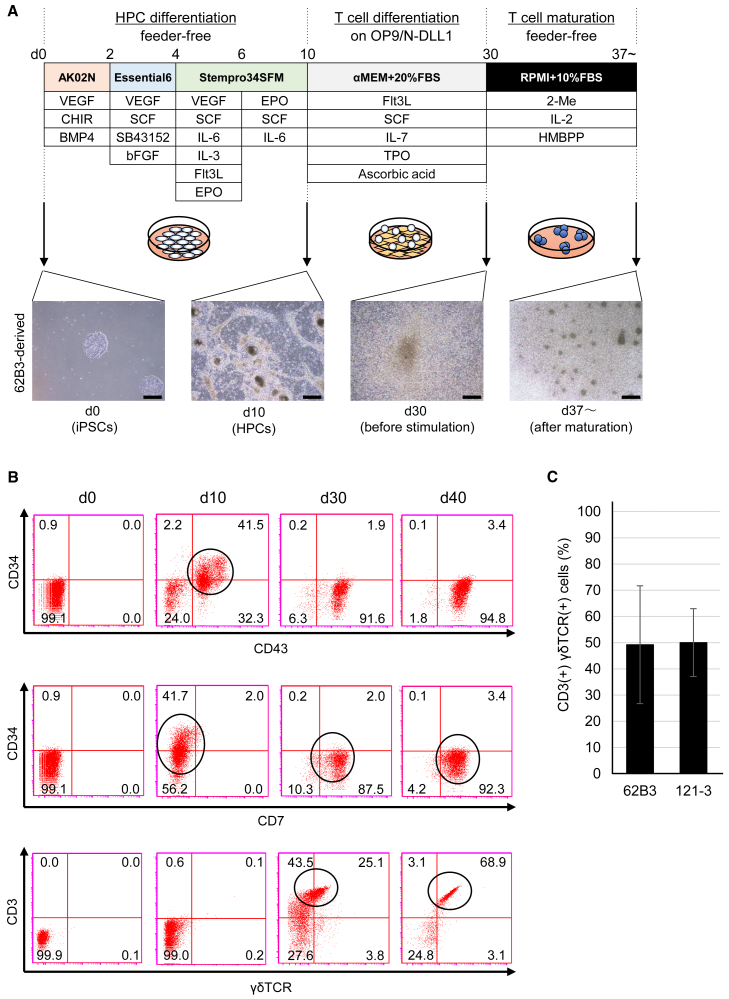
Re-differentiation of γδT-iPSCs into γδT cells (A) Schematic diagram of the protocol for differentiation from γδT-iPSC into γδT cells is shown in the upper panel. Representative phase-contrast images of iPSCs (day 0) and iPSC derivatives (day 10, day 30, and day 37∼) are shown in the lower panels. Scale bars, 500 μm. (B) Cell surface markers were analyzed by flow cytometry on days 0, 10, 30, and 40. The circle in scattergram indicates HPCs at day 10, immature T cells at day 30, and mature T cells at day 40. (C) The proportions of CD3(+)γδTCR(+) cells in the derivatives of γδT-iPSCs (n = 4 independent experiments, mean ± SD).

Next, we re-differentiated these γδT-iPSCs into γδT cells according to previously reported protocols (Kutlesa et al., 2009; Watanabe et al., 2018) with slight modifications shown in Figure 1A. On day 10, we confirmed the induction of cells positive for both CD34 and CD43 (Figure 1B upper panels), the subset of which was shown to be hematopoietic progenitor cells (HPCs) (Timmermans et al., 2009). At this time point, no cells expressed CD7, CD3, or γδTCR (Figure 1B middle and lower panels). From day 10, the derivatives of γδT-iPS were co-cultured with OP9/N-DLL1 feeder cells, which have been commonly used for differentiation of HPCs into T lymphocytes (Kutlesa et al., 2009; Schmitt et al., 2004). Thereafter, the expression of CD34 gradually became negative, and cells positive for CD7, a pre-lymphoid and mature T cell marker (Ohishi et al., 2002; Timmermans et al., 2009), increased (Figure 1B middle panels).

On day 30, the expression of CD3 was clearly positive, while the expression of γδTCR was still weak (Figure 1B lower panels). To differentiate not only nonadherent differentiated cells but also more immature cells adhering to the feeder (Jing et al., 2010), we collected all cells, including feeder cells, and transferred them into a feeder-free dish. We then started γδTCR stimulation with (E)-4-Hydroxy-3-methyl-but-2-enyl diphosphate (HMBPP) (Figure 1A), which is a metabolite in a non-mevalonate pathway and which is known to activate Vγ9Vδ2 T cells (Nerdal et al., 2016). Although some feeder cells adhered to the new dish, they peeled off after several days (data not shown). Seven to 10 days after the start of γδTCR stimulation, we found cell aggregations with phase-contrast microscopy (Figure 1A), and most of the cells became clearly positive for both CD3 and γδTCR (Figure 1B lower panels), suggesting the maturation of the cells to γδT cells progressed. We confirmed reproducibility of the differentiation to CD3(+) γδTCR(+) cells from two γδT-iPSC lines (Figure 1C). We named the resultant cells iγδTs. At day 40 of differentiation, we obtained up to 3 × 10^5^ iγδT cells from 2 × 10^3^ iPSCs. Even after the induction of iγδT, CD3-negative cells still existed. Although it was unclear what type of cells the CD3-negative cells were, they were at least negative for CD56 and CD335, which are known markers of NK cells (Figure S2A).

### Monoclonal γδTCR expression in iγδTs

**Figure 2:**
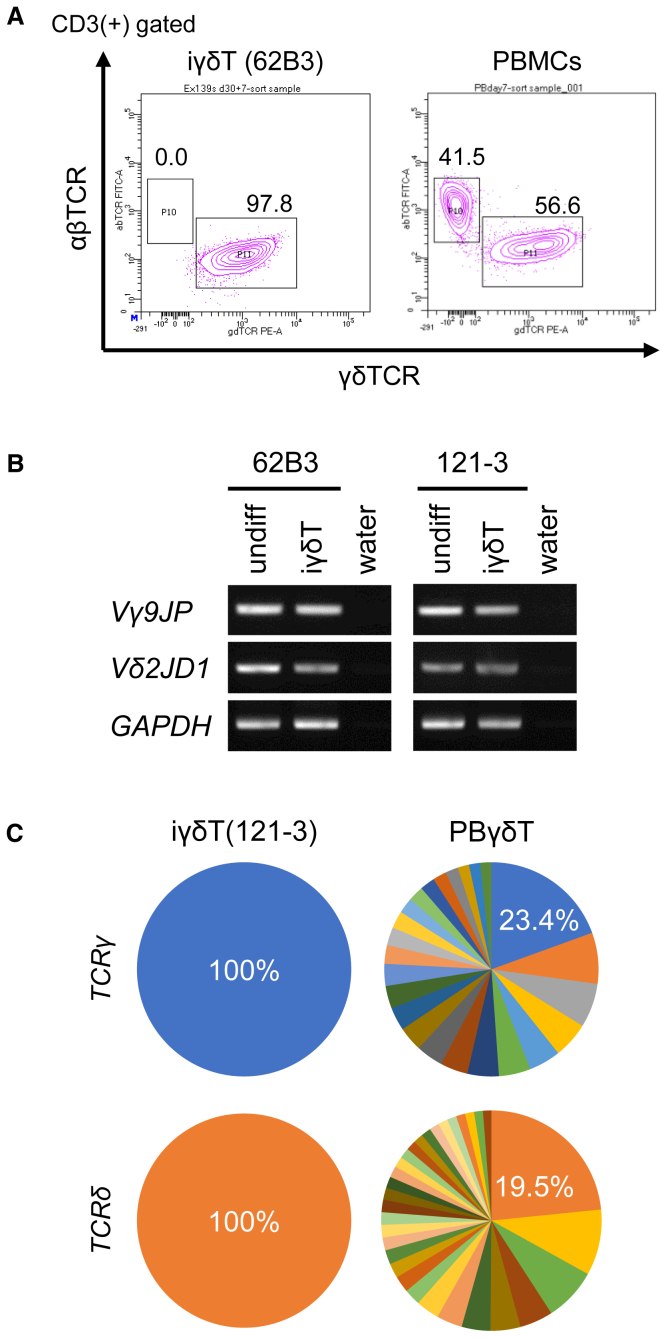
Validation of iγδTs (A) Flow cytometry to detect the expression of γδT cell receptor (γδTCR) and αβTCR in CD3(+) sorted 62B3-derived iγδTs (left) and PBMCs (right). Both were stimulated with HMBPP and IL-2 for 7 days. (B) Genomic PCR to detect TCR gene rearrangement in undifferentiated original iPSC (undiff) and CD3(+) γδTCR(+) sorted iγδTs derived from the iPSC lines (iγδT). (C) The circle graph indicates the variance of the VDJ sequence in iγδTs and PBγδTs. The number indicates the proportion of the dominant repertoire.

Next, we examined whether the iγδTs expressed a monoclonal γδTCR, as theoretically expected. In contrast to CD3-positive cells in peripheral blood mononuclear cells (PBMCs) stimulated with HMBPP for 1 week being composed of both γδTCR-positive and αβTCR-positive cells, CD3-positive iγδTs contained no αβTCR-positive cells (Figure 2A). Genomic PCR to detect *TCRG* and *TCRD* genes showed that Vγ9-to-JP and Vδ2-to-JD1 recombination in γδT-iPSCs was retained in iγδTs (Figure 2B). Furthermore, we performed an analysis of the TCR γ and TCR δ repertoire of CD3(+) γδTCR(+) cells sorted from the iγδTs as well as HMBPP-stimulated PBγδTs. The results of the amino acid sequences in the CD3R lesion, which guarantee reliability only for amino acid sequences with a frequency of more than 1%, demonstrated that PBγδTs consisted of more than 25 clones (Figure 2C right panels; Table S1), whereas iγδTs consisted of only a single clone (Figure 2C left panels; Table S2)).

These data indicated that we successfully re-generated monoclonal γ9δ2 T cells via γδT-iPSCs.

### Cytotoxicity of iγδTs against cancer cell lines

A key advantage of γδT cells for cancer immunotherapy is that one type of γδT cell is applicable for various types of cancer in a human leukocyte antigen (HLA)-unrestricted manner (Wrobel et al., 2007). We therefore evaluated the toxicity of the iγδTs against four types of cancer cell lines, including two non-solid tumor (Jurkat cells [an acute T cell leukemia cell line] and K562 cells [a chronic myelogenous leukemia cell line]) and two solid tumors (Huh-7 cells [a hepatocellular carcinoma cell line] and SW480 cells [a colorectal adenocarcinoma cell line]). We confirmed that these cell lines have different HLA types from the two iPSC lines used in this study (Table 1).

**Table 1:** HLA phenotyping of iPSC and cancer cell lines

	HLA-A		HLA-B		HLA-C		HLA-DRB1	
62B3	02:01	24:02	40:01	54:01	01:02	03:04	04:03	04:05
121-3	24:02	31:01	35:01	52:01	04:01	12:02	09:01	13:02
Jurkat	03:01	–	07:02	35:03	04:01	07:02	07:01	15:01
SW480	02:01	24:02	07:02	15:18	07:02	07:04	01:03	13:01
K562	31:01	–	40:01	50:01	03:04	05:01	03:04	–

**Figure 3:**
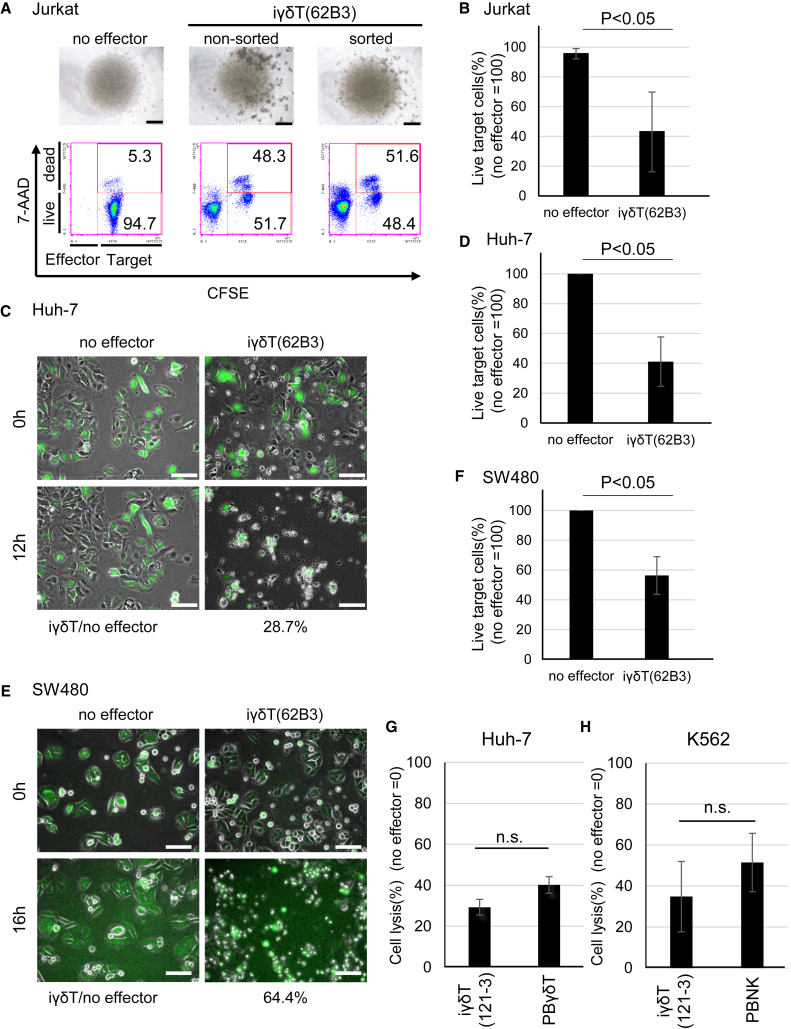
Cytotoxicity of iγδTs against cancer cell lines (A) Phase-contrast images and dot plots for CFSE and 7-AAD staining of Jurkat cells after co-culture for 16 h with no-effector cells (left), whole iγδTs (non-sorted, middle) and CD3(+) γδTCR(+) sorted iγδTs (sorted, right) (E:T ratio = 2:1). Scale bars, 500 μm in upper panels. Flow cytometry was performed to determine the percentage of live (CFSE[+] and 7-AAD[−]) or dead (CFSE[+] and 7-AAD[+]) cells in target Jurkat cells. (B) The proportions of live Jurkat cells after co-culture with or without 62B3-derived iγδTs for 1 day (n = 8 independent experiments, mean ± SD, two-tailed paired t test). (C) Representative continuous images at 0 and 12 h displaying GFP-Huh-7 cells co-cultured with or without 62B3-derived iγδTs. The proportion of the GFP-positive area in target cells co-cultured with iγδTs for 12 h was 28.7% of the GFP-positive area of the target cells with no effector, which was set to 100%. See also Figure S3B. Scale bars indicate 100 μm. (D) The relative proportion of live GFP-Huh-7 cells after co-culture with or without iγδTs was calculated according to the GFP-positive area. The proportion of the no-effector group at 12 h was set to 100%, as a control (n = 3 independent experiments, mean ± SD, two-tailed paired t test). (E) Representative continuous images at 0 and 16 h displaying CFSE-stained SW480 cells co-cultured with or without 62B3-derived iγδTs. The proportion of the CFSE-positive area at 16 h in target cells co-cultured with iγδTs was 64.4% of the CFSE-positive area of the target cells with no effector, which was set to 100%. Scale bars indicate 100 μm. (F) The relative proportion of live CFSE-stained SW480 cells after co-culture with or without iγδTs was calculated by CFSE-positive area. The proportion of the no-effector group at 16 h was set to 100%, as a control (n = 3 independent experiments, mean ± SD, two-tailed paired t test). (G) Huh-7 cells were incubated with CD3(+) sorted iγδTs or PBγδTs at an E:T ratio of 2:1 and the cytotoxicity was determined using an xCELLigence RTCA system. The proportion of the no-effector group was set to 0%, as a control (n = 3 independent experiments, mean ± SD, two-tailed paired t test). (H) K562 cells were incubated with CD3(+) sorted iγδT or PBNK cells, and the cytotoxicity was determined using an xCELLigence RTCA system. The proportion of the no-effector group was set to 0%, as a control (n = 3 independent experiments, mean ± SD, two-tailed paired t test).

First, we labeled Jurkat cells with carboxyfluorescein diacetate succinimidyl ester (CFSE) as target cells, co-cultured with the iγδTs as effector cells at an effector (E):target (T) ratio of 2:1 and stained these cells with 7-aminoactinomycin-D (7-AAD) to identify dead cells, followed by flow cytometry (FCM). The no-effector condition showed that only approximately 5% of Jurkat cells were dead (Figure 3A left panel). In contrast, the derivatives of iPSCs showed obvious cytotoxicity; co-culture with effector cells for 1 day resulted in cell death of approximately half of the target cells, regardless of whether or not CD3 and γδTCR co-positive cells were sorted (Figure 3A middle and right panels). Accordingly, we decided to use unsorted iγδTs as effector cells for the subsequent cytotoxicity assays to avoid cellular damage caused by sorting. We confirmed the reproducibility of cytotoxicity using a γδT-iPSC line, 62B3-derived iγδTs, generated in eight independent differentiation experiments. All the experiments showed the cytotoxicity of iγδTs toward Jurkat cells (Figure 3B), although the magnitude of efficacy varied from experiment to experiment. Another γδT-iPSC line, 121-3-derived iγδTs, also showed cytotoxicity toward Jurkat cells (Figure S3A).

To assess the cytotoxicity of iγδTs toward solid tumor cells, we observed the co-culture of 62B3 γδT-iPSC line-derived iγδTs with GFP-Huh-7 cells by time-lapse imaging. After 12 h of co-culture, the areas of GFP-Huh-7 cells decreased to 28.7%, 30.1%, and 64.5% of those in no-effector control culture in three independent experiments (Figures 3C and 3D). Moreover, we were able to catch iγδTs coming into contact with GFP-Huh-7 cells and peeling off as time went on (Video S1). The change in the area of GFP-Huh-7 cells each hour is shown in Figure S3B. Similarly, co-culture of CFSE-labeled SW480 and the derivatives of a γδT-iPSC line, 62B3, resulted in a decrease in the areas of SW480 cells to 38.6%, 64.4%, and 66.0% of those of the no-effector control at 16 h in three independent experiments (Figures 3E and 3F). Another γδT-iPSC line, 121-3-derived iγδTs, also showed cytotoxicity toward Huh-7 cells (Figure S3C) and SW480 cells (Figure S3D).

Video S1. Cytotoxicity toward Huh-7 cells This video displays the continuous imaging of iγδTs co-cultured with GFP-labeled Huh-7 cells at an E:T ratio of 2:1 from 0 to 6 h. The scale bar (upper left) indicates 100 μm.

Next, to confirm the cytotoxicity of purified-iγδTs, we co-cultured CD3-MACS-purified iγδT cells and tumor cells and quantified the tumor toxicity at an E:T ratio of 2:1 at 12 h using xCELLigence. Against Huh-7 cells, the purified iγδT and PBγδT cells showed no significant difference in cytotoxicity (Figure 3G). Moreover, because γδT cells reportedly express NK receptor molecules, such as NKG2D (Rincon-Orozco et al., 2005), we performed a cytotoxicity assay using K562 cells, which are known to be killed by NK cells, as target cells. iγδT cells and peripheral blood-derived NK (PBNK) cells showed similar cytotoxic activity against K562 cells, with no significant difference (Figure 3H).

These data demonstrated that the iγδTs have cytotoxicity for at least four different types of cancer cells in an HLA-unrestricted manner. Moreover, we were able to catch iγδTs coming into contact with GFP-Huh-7 cells and peeling off as time went on (Video S1).

### Mode of action of iγδTs

**Figure 4:**
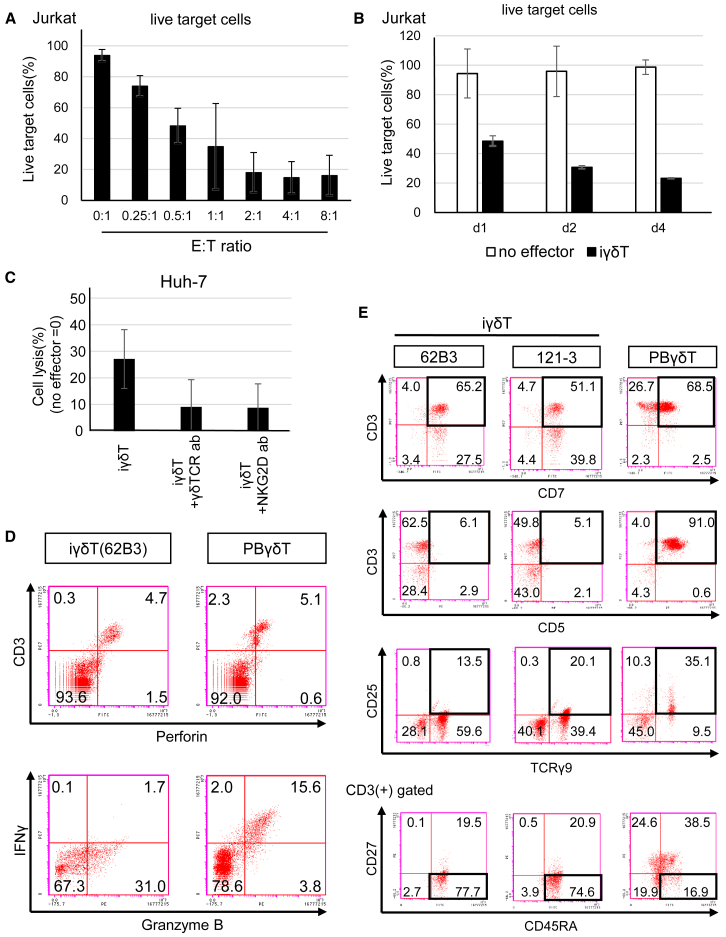
Modes of action of iγδTs and comparison with PBγδTs (A) The percentage of live Jurkat cells after co-culture with 62B3-derived iγδTs at the indicated E:T ratios for 1 day (n = 3 independent experiments, mean ± SD). See also Figure S3E. (B) The time course analysis of live Jurkat cells co-cultured with (black bars) or without (white bars) 62B3-derived iγδTs at an E:T ratio of 0.5: 1 for 4 days (n = 3 independent experiments, mean ± SD). See also Figure S3F. (C) The percentage cytolysis of Huh-7 cells after 12 h of co-culture with or without neutralizing antibodies (20 μg/mL). The percentage cytolysis of the no-effector group was set to 0%, as a control (n = 3 independent experiments, mean ± SD). (D) Flow cytometry to detect cytotoxic molecules. PBγδTs and iγδTs were pre-incubated with Brefeldin A and co-cultured with Jurkat cells at the E:T ratio of 2:1 for 4 h. In the upper panels, effector cells were pre-labeled with a CD3 antibody before the start of co-culture. (E) The expression of T cell-related markers were analyzed by flow cytometry in γδT-iPSC-derived iγδTs and PBγδTs. The cells were stimulated with HMBPP and IL-2 for 10 days and sorting was not performed.

We next performed several experiments to obtain insight into the mode of action of the iγδTs. The co-culture of iγδTs and Jurkat cells at various E:T ratios showed the dose-dependent cytotoxic effects of iγδTs (Figures 4A and S3E). Notably, even at an E:T ratio of only 0.25:1, cytotoxicity was clearly observed and reached a plateau at 2:1, while a previous report on iPSC-derived T cells showed their cytotoxicity toward lymphoma cell lines at E:T ratios of greater than 20:1 (Themeli et al., 2013). To evaluate the persistence of cytotoxicity, we co-cultured iγδTs and Jurkat cells at a low E:T ratio of 0.5:1 for up to 4 days. The results showed that cytotoxicity increased in a time-dependent manner and lasted for at least 4 days (Figures 4B and S3F).

Next, we investigated the mechanism by which iγδTs exhibit cytotoxicity. Both blocking antibodies for γδTCR and NKG2D reduced the cytotoxicity of the purified iγδTs, suggesting that the iγδTs recognize tumor cells by both γδTCR and NKG2D (Figure 4C). Perforin, granzyme B, and interferon gamma (IFNγ) are reported to play important roles in the cytotoxicity of γδT cells (O'Neill et al., 2020). To determine whether iγδTs release these factors, iγδTs re-generated from the 62B3 γδT-iPSC line and Jurkat cells were co-cultured with the addition of Brefeldin A, which blocks the transportation of proteins to the Golgi bodies and induces the accumulation of proteins in the ER. The iγδTs were pre-labeled with a CD3 antibody before co-culture to distinguish iγδTs from Jurkat cells. At 4 h of co-culture, the cells were fixed with 4% PFA and analyzed by FCM. Granzyme B and perforin, which are expressed in cytotoxic T cells (Brandes et al., 2009; Voskoboinik et al., 2015), were expressed in both the iγδTs and PBγδT cells (Figure 4D), suggesting that the iγδTs, like authentic γδT, directly attach to and attack tumor cells with lytic granules carried by secretory lysosomes.

Notably, no iγδTs were positive for IFNγ. In contrast, most granzyme B-positive cells in PBγδT cells were positive for IFNγ (Figure 4D). This finding raised the question as to whether iγδTs have other distinct molecular signatures from PBγδTs.

### Comparison of cell surface markers in iγδTs and PBγδTs

We compared the cell surface markers of iγδTs with PBγδT cells by FCM (Figure 4E). Both derivatives of the two γδT-iPSC lines (62B3 and 121-3) and PBMCs were stimulated with HMBPP and interleukin (IL)-2 for 10 days (iγδTs, from day 30 of differentiation; PBγδTs, from the first day of culture). In iγδTs, >90% of CD3(+) cells were positive for CD7, whereas <80% of CD3(+) cells were positive for CD7 in PBγδTs (Figure 4E uppermost panels). Moreover, CD3(+) cells were also positive for CD5 in <20% of iγδTs and >90% of PBγδT cells (Figure 4E second row of panels). In TCRγ9(+) cells, <50% of the cells were positive for CD25 (IL2RA) in iγδTs, whereas >85% of PBγδTs were positive (Figure 4E third row of panels).

In general, T cells are divided into four subsets of naive or memory phenotypes corresponding to the CD45RA and CD27 expression patterns (Berard and Tough, 2002). Despite stimulation during the same period, most iγδTs showed a CD45RA(+) CD27(−) phenotype. In contrast, PBγδTs existed in all four subsets (Figure 4E bottom panels). CD45RA(+) CD27(−) γδT cells have been reported to correspond to terminally differentiated effector memory T cells, which have a low expansion capacity (Odaira et al., 2016). Although the significance of the expression patterns of CD45RA and CD27 in γδT cells remains unclear, the expression patterns of these molecules also differ between iγδTs and PBγδTs.

### scRNA-seq reveals distinct populations of γδT cells in PBγδT cells and iγδTs

**Figure 5:**
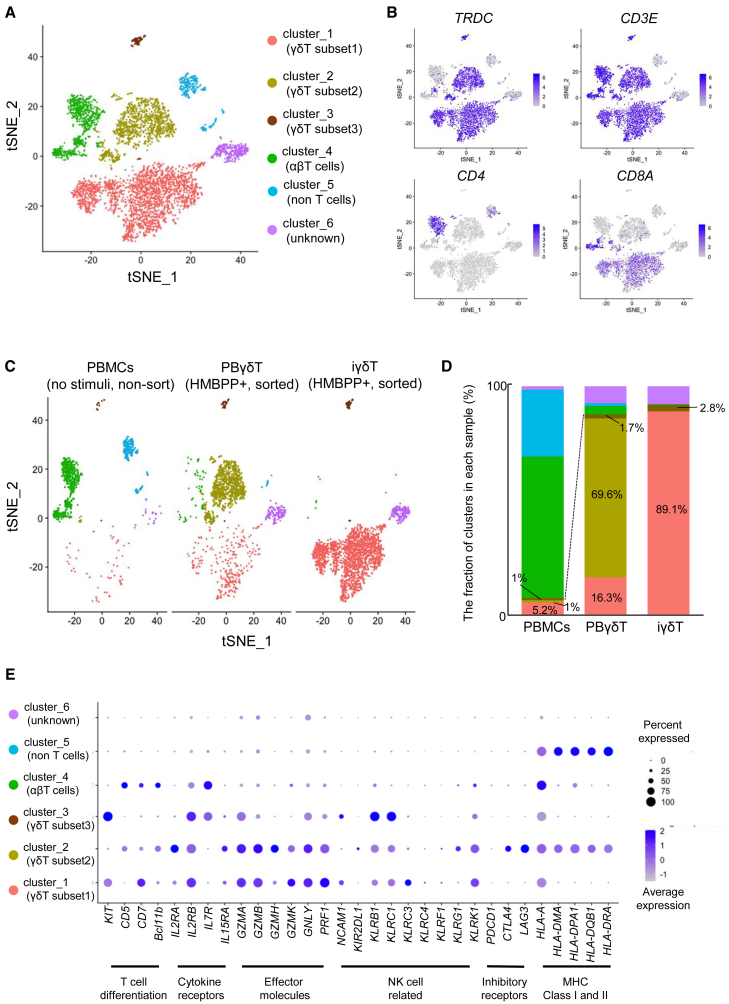
Clustering by single-cell RNA-seq (A) Combined scRNA-seq analysis of freshly isolated PBMCs, PBγδTs, and iγδTs. t-SNE visualization showing six clusters. (B) Feature plots show the expression of marker genes to define clusters. Blue indicates a high expression level; light gray indicates that the gene was not expressed. See also Figure S4A. (C and D) Comparison of cluster distribution across three samples illustrated by split t-SNE (C) and a bar graph (D). Freshly isolated PBMCs, unstimulated and unsorted PBMCs; PBγδT, *in vitro* expanded with HMBPP and CD3(+) γδTCR(+) sorted peripheral blood-derived γδT cells; iγδT, *in vitro* expanded with HMBPP and CD3(+) γδTCR(+) sorted iγδTs. (E) Dot plot showing the expression of immune-related genes for the cells in each cluster. Dot size represents the percentage of cells expressing the genes; color scale represents the gene expression level.

γδT cells have been reported to have various subtypes (Lawand et al., 2017; Li et al., 2020; Wu et al., 2017) and it was found that iγδTs and PBγδTs show different expression patterns in the bulk state from the verification of cell surface markers. To examine whether there are differences in the subtypes of γδT cells between iγδTs and PBγδT cells, we performed targeted single-cell RNA sequencing (scRNA-seq) of the following three types of cells: (1) freshly isolated PBMCs (no stimulation and no sorting). (2) PBγδT cells; PBMCs were expanded with HMBPP *in vitro* for 7 days and CD3(+) γδTCR(+) cells were sorted. (iii) iγδTs; differentiated cells from γδT-iPSC clone 62B3 (according to the protocol shown in Figure 1A) were stimulated with HMBPP for 6–12 days in three independent experiments and CD3(+) γδTCR(+) cells were sorted. Unsupervised clustering of three datasets (freshly isolated PBMCs, PBγδT cells, and one of three iγδT samples) identified six distinct cell clusters, which was shown by t-distributed stochastic neighbor embedding (t-SNE) (Figure 5A).

Cells in cluster 1 were characterized by the expression pattern of *TRDC*(+), *CD3E*(+), *CD4*(−), *CD8A*(+), and *CD8B*(−) (Figures 5B and S4A), and we categorized these as γδT subset 1. Cells in clusters 2 and 3 were characterized by the expression pattern of *TRDC*(+), *CD3E*(+), *CD4*(−) *CD8A*(−), and *CD8B*(−) (Figures 5B and S4A), and we categorized these as γδT subset 2 and γδT subset 3, respectively. Cells in cluster 4, which expressed either *CD4* or *CD8A*/*CD8B*, were categorized as αβT cells. These cells also expressed *SELL* (Figure S4B). Cells in cluster 5 were non-T cells, consisting of *MS4A1+* B cells and *S100A9+* monocytes (Figure S4C). Cells in cluster 6 partially expressed *TRDC* and *CD3E* (Figure 5B), but did not have other features, except that their expression of *LGALS1* was higher compared with cells in other clusters (Figure S4D). We categorized these as unknown.

The t-SNE distribution of each sample and the fraction of clusters in each sample are shown in Figures 5C and 5D, respectively. As expected, freshly isolated PBMCs were mostly occupied by αβT cells (cluster 4) and non-T cells (cluster 5) and contained 5.2%, 1%, and 1% of γδT subsets 1, 2, and 3, respectively (Figure 5D left bar graph). In PBγδT cells, γδT subset 2 accounted for approximately 70% of the total with 16.3% of γδT subset 1 and 1.7% of γδT subset 3 (Figure 5D middle bar graph). On the other hand, γδT subset 1 accounted for the majority (89.1%) in iγδTs. The rest was γδT subset 3 at 2.8%, and γδT subset 2 was completely absent (Figure 5D right bar graph). These results indicated that the major γδT subsets differ between PBγδT cells and iγδTs, and that cells similar to the major subset of iγδTs exist in PBγδT as a minor subset as well as in PBMCs.

To examine the expression of major immune-related genes in each γδT subset, we created dot plots for T cell-differentiation marker genes (Terstappen et al., 1992; Tydell et al., 2007), cytokine receptors (Caccamo et al., 2005; Corpuz et al., 2016; Sugamura et al., 1996), effector molecules (Fan and Zhang, 2005; O'Neill et al., 2020; Yang et al., 2020), NK cell-related genes (Yang et al., 2020), inhibitory receptors (Themeli et al., 2013), and MHC class I and II (Hurley, 2021; Karakikes et al., 2012; Yang et al., 2020) (Figure 5E). These data suggest that the expression levels of immune-related genes and the proportion of cells expressing them differed among the γδT subsets.

### Differentially expressed genes in each γδT cell subset

**Figure 6:**
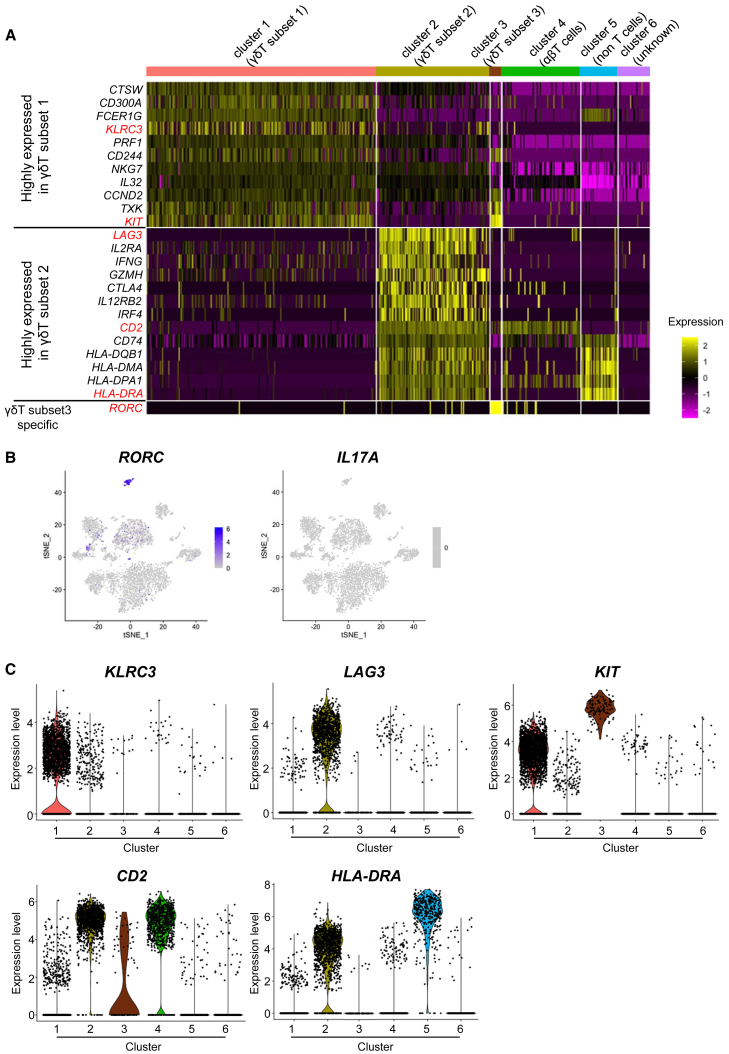
Extraction of genes with characteristic expression in each γδT cell subset (A) A heatmap of genes that were highly expressed in γδT subset 1 (n = 11), γδT subset 2 (n = 13), and γδT subset 3 (n = 1) in the analyzed cells. Each column represents the gene expression profile of a single cell. The gene expression is color coded with a scale based on the *Z* score distribution, from low (purple) to high (yellow). (B) Feature plots show the expression of marker genes. Blue indicates a high expression level; light gray indicates that the gene was not expressed. (C) Violin plots showing the expression of selected genes from each cluster.

Next, we extracted differentially expressed genes in each cluster against the rest of the clusters (e.g., cluster 1 vs. the mean of clusters 2–6). Cells in γδT subset 1, the main population of iγδTs, were enriched for NK cell-related genes (e.g., *CTSW*, *FCER1G*, *KLRC3*, *CD244*, *NKG7*, as well as the cytotoxic marker perforin coding gene [*PRF1*]). Cells in γδT subset 2, the dominant population of *in vitro*-expanded PBγδTs stimulated with HMBPP, expressed immune checkpoint inhibitory receptors (*PDCD1* [PD-1], *CTLA4*, and *LAG3*) (Figure 5E). The dominant population of iγδT were positive for the expression of these inhibitory receptor genes in cells in γδT subset 1, but the expression levels were lower than those in γδT subset 2. Antigen-presenting genes (*CD74*, *HLA-DQB1*, *HLA-DMA*, *HLA-DPA1*, *HLA-DRA*) and *IFNγ* and IFNγ-inducing genes (*IL12RB*, *IRF4*) (Xu et al., 2010; Yao et al., 2013) were expressed at higher levels compared with cells in other clusters. The *RORC* expression was restricted to cells in γδT subset 3 (Figures 6A and 6B left panel). *IL-17A*, which was reported to be released from *RORC*+ γδT cells (Ben Hmid et al., 2020), was not expressed in cells in γδT subset 3 or in any of the other cells in this study (Figure 6B right panel). A violin plot showed that *KLRC3* and *LAG3* were specifically expressed in γδT subsets 1 and 2, respectively (Figure 6C). The expression of *KIT* was higher in the cells of γδT subsets 1 and 3 (Figures 6A and 6C). On the other hand, pan-T cell marker *CD2* and MHC class II molecule *HLA-DRA* were expressed in γδT subset 2, but not in γδT subsets 1 and 3 (Figures 6A and 6C).

In order to investigate the reproducibility of the iγδT data, the expression of these marker genes in scRNA-seq data of iγδTs prepared three times independently was shown by a heatmap. The similar expression patterns indicated that the gene expression of iγδTs was reproducible (Figure S4E).

Taken together, we successfully re-generated MHC-unrestricted cytotoxic γδT cells from iPSCs and clarified the distinctive molecular signatures of iPSC-derived γδT cells.

## Discussion

In the present study, we successfully re-generated CD3(+)γδTCR(+) cells from γδT cell-derived iPSCs. Although there have been reports of the re-generation of αβT cells from αβT cell-derived iPSCs (Maeda et al., 2016; Nishimura et al., 2013; Vizcardo et al., 2013), it was unclear whether a protocol similar to that for αβT cell differentiation from iPSCs could be applied to γδT cells, because the development process of γδT cells was reported to differ from that of αβT cells in several points. First, during fetal development, γδT cells precede αβT cells (Hayday, 2000). HPCs first differentiate into CD4/8 double-negative (DN) cells and then progress to CD4/8 double-positive (DP) cells (Seo and Taniuchi, 2016). While αβT cells differentiate from DP cells, γδT cells can differentiate from both DP and DN cells (Van Coppernolle et al., 2012). The weak and strong TCR signal strength received by DN cells favors αβT and γδT lineage development, respectively (Hayes et al., 2005). Furthermore, transcription factor Bcl11b-knockout mouse studies revealed that Bcl11b was essential for the differentiation of DN cells into αβT cells, but not necessary for differentiation into γδT cells (Ikawa et al., 2010), and these γδT cells without Bcl11b only show a CD5(−) phenotype (Hatano et al., 2017). The expression of Bcl11b and CD5 were low in our iγδTs, as shown in Figures 4E and 5E, suggesting that the development of iγδTs may be similar to that of CD5(−) γδ T cells *in vivo*.

With a scRNA-seq analysis, we revealed the distinct signatures of iγδTs from PBγδTs. They shared common clusters with a minor part of freshly isolated PBMCs and *ex vivo*-expanded PBγδTs, indicating that the cells resembling the major population of iγδTs exist in adult PBMCs in nature but are not expandable with HMBPP stimulation, at least under the culture condition used in this study. Previously there have been many studies concerning the classification of human peripheral γδT cells. For example, the functions of γδT cells were reportedly separated into five subsets: IFN-γ-producing, antigen-presenting, follicular B helper, regulatory γδT, and IL-17-producing cells (Wu et al., 2017). One other group classified γδT cells according to the effects on tumor cells: anti-tumor or tumor promoting (Li et al., 2020). Another group divided γδT cells according to the expression of activation marker genes, such as CD16 (Braakman et al., 1992), CD69 (Cibrian and Sanchez-Madrid, 2017), and RORC (Ben Hmid et al., 2020). Our iγδTs did not fully correspond to any of these previously reported γδT cell types in postnatal peripheral blood, in terms of the gene expression patterns. Notably, in contrast to PBγδTs, the iγδTs were negative for CD2 and positive for CD7. A previous report showed that a human fetal thymus-derived γδT cell clone showed a CD2 (low) CD7(+) phenotype and low IFNγ secretion (Carding et al., 1990). Our iγδTs might correspond to γδT cells derived from the fetal thymus.

The cytotoxicity of our iγδTs, which showed distinctive molecular signatures, can be supported by some previous reports. The iγδTs were CD5(−), and CD5(−) γδT cells were reported to be more cytotoxic than CD5(+) γδT cells (Srour et al., 1990). In addition, approximately three-quarters of the iγδTs showed a terminally differentiated T cell phenotype: CD45RA(+)/CD27(−). Terminally differentiated γ9δ2T cells reside in inflamed tissue, where they display an immediate effector function (Dieli et al., 2003) and exert higher cytotoxicity and lower IFNγ production compared with other subsets in terms of the CD45RA and CD27 expression pattern (Caccamo et al., 2005). Together, molecular mechanisms that link the molecular signatures of our iγδTs and their function should be clarified in future studies.

NK cell-related markers were expressed in iγδTs. A subset of authentic γδT cells reportedly expressed NK cell-related genes and recognize target cells by a similar mechanism to NK cells. In addition, it is reported that mimetic-γδ NKT cells, which expressed low T cell-related genes and high NK cell-related genes, were induced from iPSCs (Zeng et al., 2019). The shared NK cell markers support tumor direct recognition by γδT cells in an MHC-unrestricted manner (Wrobel et al., 2007). This NK-related gene expression may be responsible for the cytotoxicity of iγδTs.

We herein demonstrated that our iγδTs were completely negative for αβTCR and that they killed tumor cells in an MHC-independent manner. The negative expression of αβTCR may reduce the risk of graft-versus-host disease (Radestad et al., 2014). For this reason, there were studies in which allogenic PBγδTs were used as carriers for chimeric antigen receptor T (CAR-T) (Rozenbaum et al., 2020) and TCR-T (Ichiki et al., 2020).

Several limitations of the present study should be addressed in our future studies. First, the induction efficiency of iγδTs was not satisfactory, and we have not clarified what CD3(−) cells existing after iγδTs induction were. Second, it should be evaluated whether or not the iγδTs attack non-cancer cells of KIR-ligand mismatch recipients. Third, the iγδT induction protocol established in this study used xenogenic serum and feeder cells, which are difficult for clinical applications. We are currently trying to generate iγδTs under feeder-free and serum-free conditions (data not shown). Our technologies will advance off-the-shelf γδT cell-based immune therapy.

## Experimental procedures

### Resource availability

#### Materials availability

This study did not generate new unique reagents.

### Differentiation to γδT cells from iPSCs

Seven days before induction, human γδT-iPSCs were seeded onto a six-well plate at a density of 2.0 × 10^3^ cells and cultured in StemFit medium (Ajinomoto, AK02N).

On day 0, the medium was completely replaced by StemFit medium supplemented with 4 μM CHIR99021 (Tocris, 4423), 80 ng/mL BMP4 (R&D, 314-BP), and 80 ng/mL vascular endothelial growth factor (VEGF) (R&D, 293-VE). On day 2, the medium was replaced by Essential 6 medium (Thermo Fisher, A1516501) supplemented with 2 μM SB431542 (WAKO, 033-24631), 50 ng/mL bFGF (WAKO, 060-04543), 50 ng/mL SCF (R&D, 255-SC), and 80 ng/mL VEGF. On day 4, the medium was replaced by StemPRO-34 SFM (Thermo Fisher, 10639-011) supplemented with 2 mM L-glutamine, 50 ng/mL IL-3 (Peprotech, AF-200-03), 50 ng/mL IL-6 (R&D, 206-IL), 50 ng/mL FLT3L (R&D, 308-FK), 50 ng/mL SCF, 20 ng/mL VEGF, and 10 IU/mL EPO (ESPO, Kyowa Kirin).

On days 6 and 8, the medium was replaced with StemPRO-34 SFM supplemented with 2 mM L-glutamine, 50 ng/mL IL-6, 50 ng/mL SCF, and 10 IU/mL EPO.

On day 10, hematopoietic cells were transferred into wells co-cultured with feeder cells. Floating cells and supernatant were collected in the tube. Adhesive cells were dissociated with Accutase (Nacalai Tesque, 12679-54), and incubated at 37°C for 10 min. Supernatant was returned to the well, pipetted, and filtered using a 35-μm cell strainer. After centrifugation at 1,200 rpm for 4 min, cells were suspended in OP9 medium supplemented with 10 ng/mL SCF, 10 ng/mL TPO (Peprotech, AF-300-18), 5 ng/mL IL-7 (R&D, 207-IL), 5 ng/mL FLT3L, and 100 μg/mL L-ascorbic acid (Nacalai Tesque, 30264-56). Cells were reseeded to the same well and incubated at 37°C for 30 min. Without pipetting, supernatant and floating cells were transferred into a new well confluent with pre-seeded OP9/N-DLL1 cells.

On day 12, half of the medium was changed and cells were transferred into new wells with fresh OP9/N-DLL1 cells by vigorous pipetting. Then, half of the medium was changed every other day and cells were transferred onto fresh OP9/N-DLL1 cells every 6 days.

On day 30, we collected cells with Accutase, similarly to day 10. Cells were suspended with RPMI1640 medium supplemented with 10% FBS, 1 nM HMBPP (Cayman Chemical, 13580), 100 IU/mL IL-2 (Shionogi Pharmaceuticals, Imunace), and 10 μM 2-mercaptoethanol and seeded onto new plates in a feeder-free condition. Half of the medium was changed every other day. After more than a week of stimulation, cytotoxicity was analyzed.

### scRNA-seq

The day before the targeted scRNA-seq analysis, CD3(+)γδTCR(+) cells were sorted on a BD FACS Aria III from PBγδTs and iγδTs that were stimulated with HMBPP for the indicated days as described above. To infer the origin of the sample, all cells were labeled with multiplex sample tags. Single-cell capture and cDNA library preparation were performed using a BD Rhapsody Single-Cell Analysis System with a BD Human Single-Cell Multiplexing Kit (BD Biosciences, #633781) and BD Human Immune Response Targeted Panel for Human (BD Biosciences, #633750), which contains 399 primer pairs, targeting 397 different genes, according to the manufacturer’s recommendations. The concentration, size, and integrity of the resulting PCR products were assessed using a Qubit High-Sensitivity dsDNA Kit.

Sequencing was performed using an Illumina HiSeq X (Illumina, San Diego, CA) in Macrogen (Tokyo, Japan). Fastq files were uploaded to the Seven Bridges Genomics online platform. The obtained counts were adjusted by distribution-based error correction (DBEC), an error correction algorithm developed by BD Biosciences. DBEC data were then loaded into Seurat (version 4.0.4.). Cells were then clustered using a resolution of 0.03 and visualized by t-SNE. The Seurat functions FeaturePlot, DotPlot, DoHeatmap, and Vlnplot were used to visualize the gene expression with feature plot, dot plot, heatmap, and violin plot, respectively. Markers for a specific cluster against all remaining cells were found by using the Seurat function FindAllMarkers.

ScRNA-seq data have been deposited in GEO under accession number GSE194072.

### Statistical analysis

Data are expressed as the mean ± SD. Differences between two groups were analyzed using a paired t test. Statistical analyses were performed using Microsoft Excel 2013 and EZR. p values of <0.05 were considered statistically significant.

## Author contributions

Conceptualization, T.A; methodology, N.M; software, T.A. and M.K.-A; validation, M.K.-A.; formal analysis M.K.-A.; investigation, N.M.; resources, N.M.; writing – original draft, N.M.; writing – review & editing, T.A., M.K.-A.; supervision, T.A. and H.T.; project administration, T.A.

## Data Availability

The accession number for the scRNAseq reported in this paper is GEO: GSE194072.

## Supplemental information

Document S1. Figures S1–S4, Tables S1–S5, and supplemental experimental procedure

Document S2. Article plus supplemental information
