# Supplementary material for: Re-generation of cytotoxic γδT cells with distinctive signatures from human γδT-derived iPSCs
Source: Stem Cell Reports. 2023 Mar 23;18(4):853–68. doi: 10.1016/j.stemcr.2023.02.010 (PMC10147660; doi:10.1016/j.stemcr.2023.02.010)
Supplement: Document S1. Figures S1–S4, Tables S1–S5, and supplemental experimental procedure [file mmc1.pdf]

Stem Cell Reports, Volume *18*

Supplemental Information

Re-generation of cytotoxic γδT cells with distinctive signatures from human γδT-derived iPSCs

Nobuyuki Murai, Michiyo Koyanagi-Aoi, Hiroto Terashi, and Takashi Aoi

Figure.S1

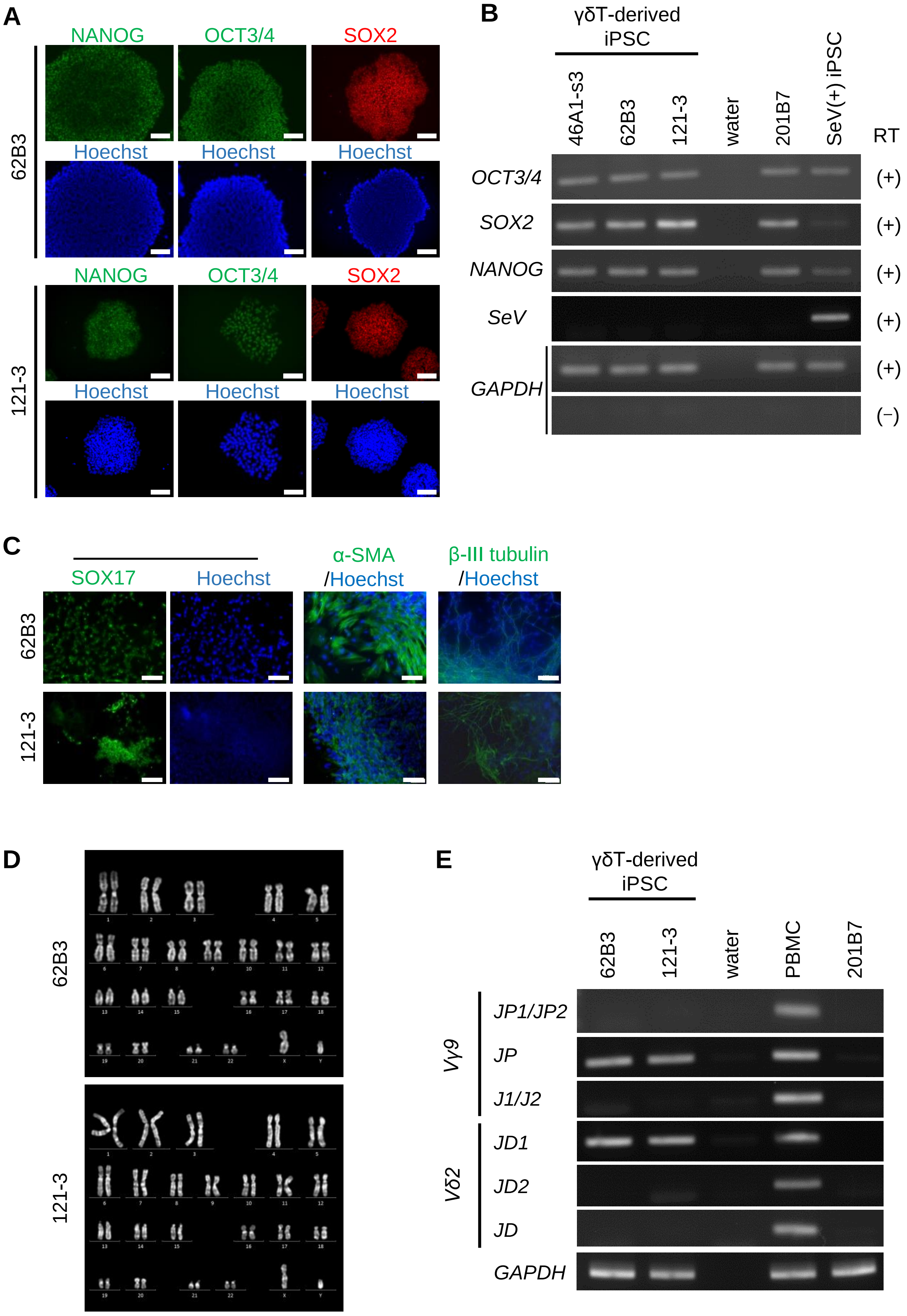
A
NANOG
OCT3/4
SOX2
Hoechst
62B3
121-3
B
γδT-derived iPSC
46A1-s3
62B3
121-3
water
201B7
SeV(+) iPSC
RT
OCT3/4
SOX2
NANOG
SeV
GAPDH
(+)
(−)
C
SOX17
Hoechst
α-SMA /Hoechst
β-III tubulin /Hoechst
62B3
121-3
D
62B3
121-3
E
γδT-derived iPSC
62B3
121-3
water
PBMC
201B7
Vγ9
JP1/JP2
JP
J1/J2
Vδ2
JD1
JD2
JD
GAPDH

Figure.S2

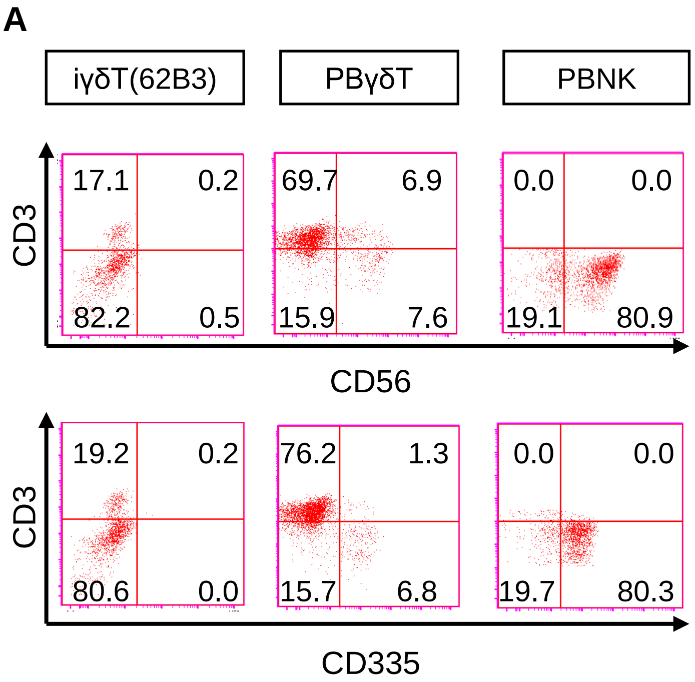
A
iγδT(62B3)
PBγδT
PBNK
CD3
17.1
0.2
82.2
0.5
69.7
6.9
15.9
7.6
0.0
0.0
19.1
80.9
CD56
CD3
19.2
0.2
80.6
0.0
76.2
1.3
15.7
6.8
0.0
0.0
19.7
80.3
CD335

Figure.S3

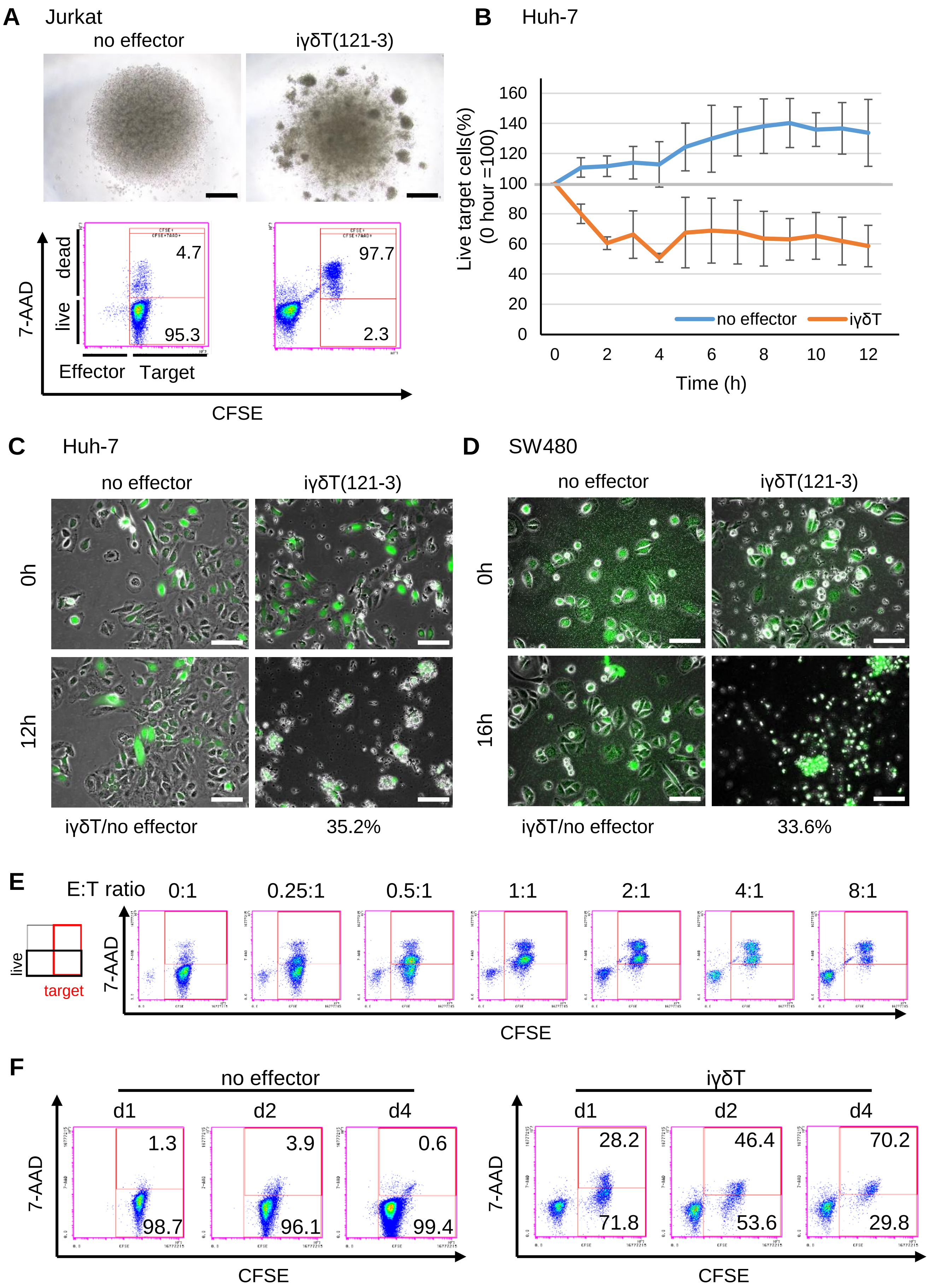
A
Jurkat
no effector
iγδT(121-3)
7-AAD
dead
live
4.7
95.3
97.7
2.3
Effector
Target
CFSE
B
Huh-7
Live target cells(%)
(0 hour =100)
160
140
120
100
80
60
40
20
0
0
2
4
6
8
10
12
Time (h)
no effector
iγδT
C
Huh-7
no effector
iγδT(121-3)
0h
12h
iγδT/no effector
35.2%
D
SW480
no effector
iγδT(121-3)
0h
16h
iγδT/no effector
33.6%
E
E:T ratio
0:1
0.25:1
0.5:1
1:1
2:1
4:1
8:1
live
target
7-AAD
CFSE
F
no effector
iγδT
d1
d2
d4
1.3
98.7
3.9
96.1
0.6
99.4
28.2
71.8
46.4
53.6
70.2
29.8
7-AAD
CFSE

Figure.S4

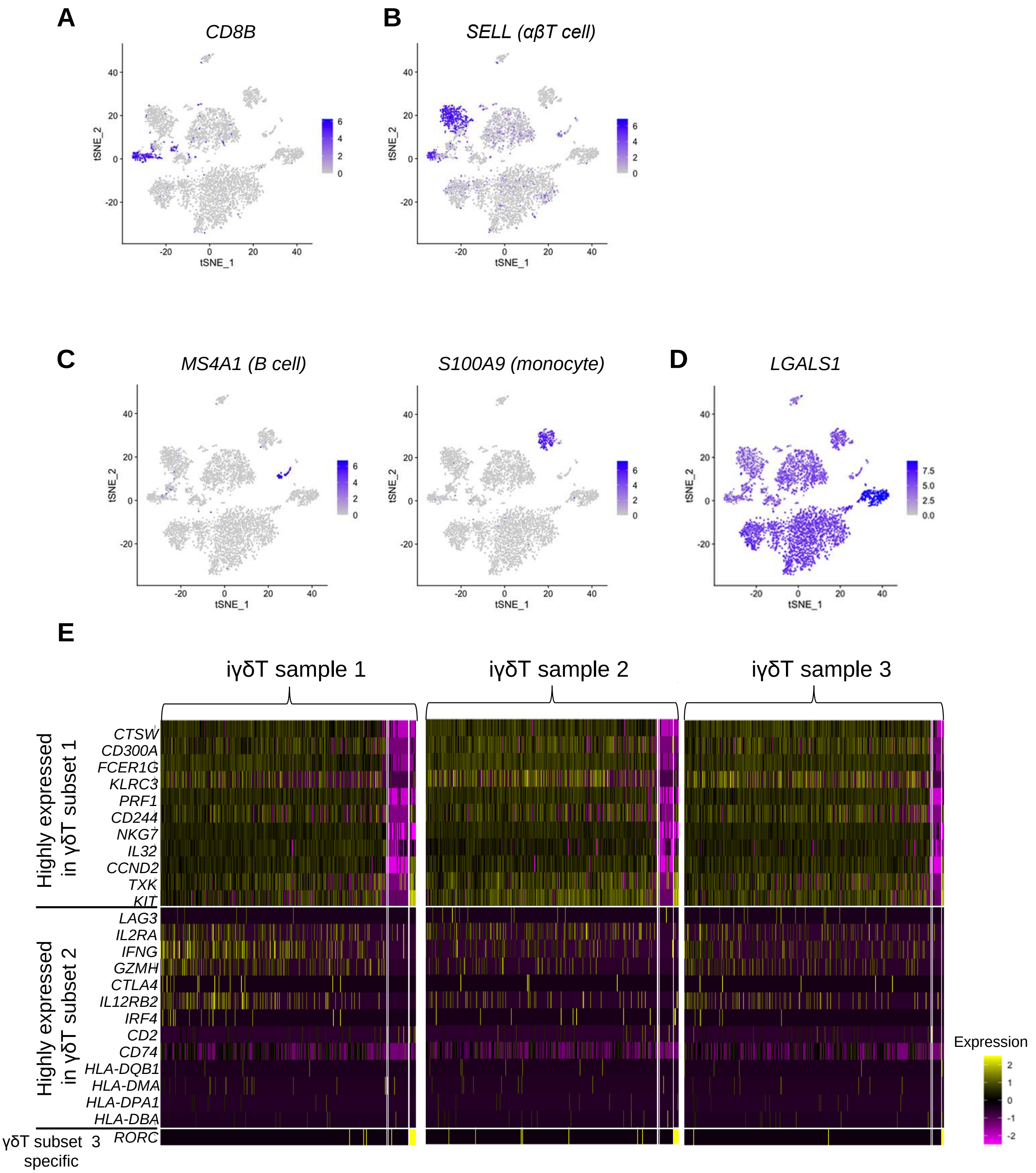

A
CD8B
B
SELL (αβT cell)
C
MS4A1 (B cell)
S100A9 (monocyte)
D
LGALS1
tSNE_1
tSNE_2
E
iγδT sample 1
iγδT sample 2
iγδT sample 3
Highly expressed in γδT subset 1
CTSW
CD300A
FCER1G
KLRC3
PRF1
CD244
NKG7
IL32
CCND2
TXK
KIT
Highly expressed in γδT subset 2
LAG3
IL2RA
IFNG
GZMH
CTLA4
IL12RB2
IRF4
CD2
CD74
HLA-DQB1
HLA-DMA
HLA-DPA1
HLA-DBA
γδT subset 3 specific
RORC
Expression

SUPPLEMENTARY FIGURE LEGENDS

Figure S1. Validation of γδT-iPSCs

A) Immunofluorescent staining of pluripotent cell markers NANOG, OCT3/4 and SOX2 in 62B3 (upper panels) and 121-3 (lower panels). Nuclei were stained with Hoechst 33342. Scale bars indicate 100 μm.

B) RT-PCR to detect pluripotent cell markers. The absence of Sendai virus (SeV) in γδT-derived iPSCs (46A1-s3, 62B3 and 121-3) was also confirmed. The conventional clone 201B7, which was established by retrovirus vectors, was used as a negative control. RT: reverse transcriptase.

C) *In vitro* differentiation via embryoid body formation of 62B3 (upper panels) and 121-3 (lower panels). Immunofluorescent staining for SOX17 (endoderm marker), α-SMA (mesoderm marker) and β-III-tubulin (ectoderm marker) in γδT-iPSCs-derived differentiated cells. Scale bars indicate 50 μm.

D) Karyotyping of γδT-iPSCs. Images of the Q-band analysis are shown.

E) Genomic PCR for TCR gene rearrangement in γδT- iPSCs. PBMCs were used as a positive control. The conventional iPSC clone 201B7, which was established from fibroblasts, was used as a negative control.

Figure S2. Flow cytometry for NK cell markers

A) Cell surface markers, including CD3/ CD56 (upper panels) and CD3/ CD335 (NKp46) (lower panels) were analyzed by flow cytometry. Each cell was stimulated (iγδT at day 48, PBγδT and PBNK at day 18) and not sorted.

Figure S3. Verification of Cytotoxicity in iγδTs

A) Phase contrast images and dot plots for CFSE and 7-AAD staining in CFSE-stained Jurkat cells (target) co-cultured with or without 121-3-derived iγδTs (effector) for one day (E:T ratio = 2: 1). Scale bars indicate 500μm (upper panels). Numbers in scattergrams indicate the percentages of live or dead cells in CFSE-stained target cells (lower panels).

B) The time course analysis of the relative proportion of live GFP-Huh-7 cells (=GFP-positive area) during co-culture with or without iγδTs. A series of image frames were acquired by fluorescence microscopy at 1 h/frame over a 12 h period. The area of GFP-positive cells at 0 h in each condition was set to 100 %. Changes in relative proportion of live target cells were shown (n=3 independent experiments, mean ±SD). See also Figure 3C.

C) Representative continuous images at 0 and 12 h displaying GFP-labeled Huh-7 cells co-cultured with or without 121-3 derived iγδTs are shown. The proportion of GFP-positive area at 12 h in target cells co-cultured with iγδT was 35.2% of that with no effector, which was set to 100%. Scale bars indicate 100 μm.

D) Representative continuous images at 0 and 16 h displaying CFSE-labeled SW480 cells co-cultured with or without 121-3 derived iγδTs are shown. The proportion of CFSE-positive area at 16 h in target cells co-cultured with iγδTs was 33.6% of that with no effector, which was set to 100%. Scale bars indicate 100 μm.

E) Dot plots showing the cytotoxicity of 62B3-derived iγδTs against Jurkat cells, which was analyzed using a CFSE/7-AAD assay with the indicated E:T ratios after one day of co-culture. See also Figure 4.

F) Dot plots showing the results of the time course analysis of the proportions of live Jurkat cells co-cultured with or without 62B3-derived iγδTs for 4 days at an E:T ratio of 0.5: 1. Numbers in scattergrams indicate the percentage of live or dead cells in CFSE-stained target cells. See also Figure 4B.

Figure S4. Characterization of each cluster and reproducibility of γδT cell induction from iPSCs

A)-D) Feature plots show the expression of CD8B (A), SELL (B), MS4A1 and S100A9 (C) and LGALS1 (D) to define clusters. Blue indicates a high expression level; light grey indicates no expression.

E) Heatmap of genes that were highly expressed in γδT subset 1 (n=11), γδT subset 2 (n=13) and γδT subset 3 (n=1) in iγδTs from three independent experiments. Three iγδT samples were resultant cells stimulated with HMBPP for 6, 11, and 12 days respectively.

SUPPLEMENTAL TABLES

Table S1. Repertoire analysis of PBγδTs.

Rearrangement of the TCRγ (TRG), TCRδ (TRD) gene locus and amino acid sequence of CDR3, related to Figure 2C.

**ranking**	***TRGV***	***TRGJ***	**CDRJ**	**%reads**	**ranking**	***TRDV***	***TRDJ***	**CDRJ**	**%reads**
1	*TRGV9*	*TRGJP*	CALWEVQELGKKIKVF	13.04841	1	*TRDV2*	*TRDJ3*	CACDSGEAGWDTRQMFF	19.46068
2	*TRGV9*	*TRGJP*	CALWEEELGKKIKVF	5.168883	2	*TRDV2*	*TRDJ1*	CACDTLLKQGDLITDKLIF	8.034431
3	*TRGV9*	*TRGJP*	CALWEVLELGKKIKVF	4.480577	3	*TRDV1*	*TRDJ1*	CALGPRYFRNWGIRPNADKLIF	6.500837
4	*TRGV10*	*TRGJP1*	CAAWDRPRRWFKIF	3.634133	4	*TRDV2*	*TRDJ1*	CACDTVDGGYAHTDKLIF	4.056395
5	*TRGV9*	*TRGJP*	CALWEVRELGKKIKVF	3.221555	5	*TRDV2*	*TRDJ1*	CACDTVGDSNTDKLIF	3.667546
6	*TRGV9*	*TRGJP*	CALWEVKAPQELGKKIKVF	3.204322	6	*TRDV2*	*TRDJ1*	CACAALPTMGMGYTDKLIF	3.502581
7	*TRGV8*	*TRGJP2*	CATWDRGSDWIKTF	3.186076	7	*TRDV2*	*TRDJ1*	CACDTVLARQYTDKLIF	3.142012
8	*TRGV9*	*TRGJP*	CALWEEFQELGKKIKVF	2.777553	8	*TRDV2*	*TRDJ3*	CACDHWGSSSWDTRQMFF	2.853322
9	*TRGV9*	*TRGJP*	CALWEAQELGKKIKVF	2.753224	9	*TRDV2*	*TRDJ3*	CACDTVSSWDTRQMFF	2.023189
10	*TRGV9*	*TRGJP*	CALWEVEELGKKIKVF	2.4704	10	*TRDV2*	*TRDJ3*	CACDTAITGGSSSWDTRQMFF	2.004925
11	*TRGV9*	*TRGJP*	CALWEALQELGKKIKVF	2.36092	11	*TRDV2*	*TRDJ1*	CACEALAYTDKLIF	1.787524
12	*TRGV9*	*TRGJP*	CALWEVQRAQELGKKIKVF	2.282864	12	*TRDV2*	*TRDJ1*	CACDTVGGGYASDKLIF	1.738034
13	*TRGV9*	*TRGJP*	CALWEMQELGKKIKVF	2.237248	13	*TRDV2*	*TRDJ1*	CACDTVGGPYTDKLIF	1.555982
14	*TRGV9*	*TRGJP*	CALWEVKELGKKIKVF	1.89056	14	*TRDV2*	*TRDJ1*	CACDTLGDRRTDKLIF	1.554215
15	*TRGV10*	*TRGJP1*	CAAWDGPTGWFKIF	1.834807	15	*TRDV2*	*TRDJ1*	CACDTLLGDKVDKLIF	1.535362
16	*TRGV9*	*TRGJP*	CALWESQELGKKIKVF	1.732422	16	*TRDV2*	*TRDJ1*	CACDTIPGGSLGTDKLIF	1.517687
17	*TRGV9*	*TRGJP*	CALWEDPELGKKIKVF	1.592531	17	*TRDV2*	*TRDJ1*	CACDIMGDTPFADKLIF	1.477034
18	*TRGV5*	*TRGJ2*	CATWDRLDYYKKLF	1.550969	18	*TRDV2*	*TRDJ1*	CACDGLGDIPYTDKLIF	1.386303

19	*TRGV8*	*TRGJ2*	CATWGRAVYYKKLF	1.512448
20	*TRGV9*	*TRGJP*	CALWEVQERELGKKIKVF	1.37053
21	*TRGV9*	*TRGJP*	CALWEVWELGKKIKVF	1.295515
22	*TRGV9*	*TRGJP*	CALWEVSEELGKKIKVF	1.161706
23	*TRGV8*	*TRGJ2*	CATWDKNYYKKLF	1.134336
24	*TRGV8*	*TRGJ2*	CATWGWTRNYYKKLF	1.117103
25	*TRGV9*	*TRGJ2*	CALWEGSNYYKKLF	1.098857
26	*TRGV9*	*TRGJ2*	CALWEVQLAGRYKKLF	0.937677
27	*TRGV9*	*TRGJP*	CALWESQQELGKKIKVF	0.90017
28	*TRGV9*	*TRGJP*	CALWEVVIELGKKIKVF	0.858608
29	*TRGV9*	*TRGJP*	CALWEEQELGKKIKVF	0.840362
30	*TRGV9*	*TRGJP*	CALWEVGELGKKIKVF	0.835293
31	*TRGV9*	*TRGJP*	CALWEAKQELGKKIKVF	0.795759
32	*TRGV3*	*TRGJ2*	CATWDISYYKKLF	0.765347
33	*TRGV9*	*TRGJP*	CALWELELGKKIKVF	0.746087
34	*TRGV9*	*TRGJ1*	CALWEVSPHKKLF	0.701484
35	*TRGV9*	*TRGJP*	CALWEESQELGKKIKVF	0.680196
36	*TRGV9*	*TRGJP*	CALWIQELGKKIKVF	0.670059
37	*TRGV9*	*TRGJP*	CALWEVRSELGKKIKVF	0.644717
38	*TRGV9*	*TRGJP*	CALWSQELGKKIKVF	0.59099
39	*TRGV9*	*TRGJP*	CALWEVPELGKKIKVF	0.580853
40	*TRGV9*	*TRGJP*	CALWEVQIGELGKKIKVF	0.577812
41	*TRGV9*	*TRGJP*	CALWEVLGLQELGKKIKVF	0.53625
42	*TRGV9*	*TRGJP*	CALWEHKGEEKLGKKIKVF	0.518003
43	*TRGV9*	*TRGJP*	CALWAAELGKKIKVF	0.512935
44	*TRGV9*	*TRGJP*	CALWEAHEELGKKIKVF	0.501784

19	*TRDV2*	*TRDJ3*	CACDTSTGGPFSWDTRQMFF	1.384536
20	*TRDV2*	*TRDJ1*	CACDTVLTGGYVDDKLIF	1.293215
21	*TRDV2*	*TRDJ1*	CACDTVGGHGGTDKLIF	1.274951
22	*TRDV2*	*TRDJ1*	CACDTVGIRGPDKLIF	1.213678
23	*TRDV2*	*TRDJ1*	CACDTLLGDTREGDKLIF	1.201306
24	*TRDV2*	*TRDJ1*	CAVLPVGHITDKLIF	1.200127
25	*TRDV2*	*TRDJ1*	CACETLDGGSQYTDKLIF	1.199538
26	*TRDV2*	*TRDJ1*	CACDPVLGDTTYTDKLIF	1.166545
27	*TRDV2*	*TRDJ1*	CACDSIGGGYASQLIF	1.140622
28	*TRDV2*	*TRDJ1*	CACDSLRPGGAHTDKLIF	1.117055
29	*TRDV2*	*TRDJ1*	CACDTAYAGSTDKLIF	1.100559
30	*TRDV2*	*TRDJ1*	CACDSVTGGYTPDKLIF	1.078759
31	*TRDV2*	*TRDJ1*	CACDTLGDQPRHTDKLIF	1.016308
32	*TRDV2*	*TRDJ1*	CACDTVKGLGENTDKLIF	0.721137
33	*TRDV2*	*TRDJ1*	CACDTIGLMLGERRDTDKLIF	0.716424
34	*TRDV2*	*TRDJ1*	CACDSLGGPYTDKLIF	0.688144
35	*TRDV2*	*TRDJ1*	CACDPWGPHTDKLIF	0.643956
36	*TRDV2*	*TRDJ1*	CACDTLPTRGVLGDTLYTDKLIF	0.592699
37	*TRDV2*	*TRDJ3*	CACDKTGGHLSSWDTRQMFF	0.586807
38	*TRDV2*	*TRDJ1*	CACRSLGEDTDKLIF	0.56383
39	*TRDV2*	*TRDJ3*	CACDTSGGYASSWDTRQMFF	0.524945
40	*TRDV2*	*TRDJ1*	CACDTFPVLGDKELIF	0.5002
41	*TRDV2*	*TRDJ3*	YACDSGEAGWDTRQMFF	0.443641
42	*TRDV2*	*TRDJ1*	CACDTILGDTSDTDKLIF	0.384724
43	*TRDV2*	*TRDJ1*	CACDDLGDTATDKLIF	0.378243
44	*TRDV2*	*TRDJ3*	CACDTGFVGGSHSWDTRQMFF	0.375298

45	*TRGV9*	*TRGJP*	CALWELQELGKKIKVF	0.497729	45	*TRDV2*	*TRDJ1*	CACDTVKRGPHLTDKLIF	0.273961
46	*TRGV9*	*TRGJP*	CALWEPTRRTQELGKKIKVF	0.495702	46	*TRDV2*	*TRDJ1*	CACDTLDPLGDTDHTDKLIF	0.244503
47	*TRGV9*	*TRGJP*	CALWEVHELGKKIKVF	0.477455	47	*TRDV2*	*TRDJ3*	CACDSEEAGWDTRQMFF	0.076591
48	*TRGV9*	*TRGJP*	CALWEADPQELGKKIKVF	0.471373	48	*TRDV2*	*TRDJ1*	CACGTLLKQGDLITDKLIF	0.074235
49	*TRGV9*	*TRGJP*	CALWEVRLELGKKIKVF	0.464277	49	*TRDV2*	*TRDJ1*	CVCDTLGDRRTDKLIF	0.060684
50	*TRGV9*	*TRGJP*	CALWEVMELGKKIKVF	0.463263	50	*TRDV2*	*TRDJ4*	CARPLIF	0.05656

Table S2. Repertoire analysis of iγδTs.

Rearrangement of the TCRγ (TRG), TCRδ (TRD) gene locus and amino acid sequence of CDR3, related to Figure 2C.

ranking	*TRGV*	*TRGJ*	CDRJ	%reads
1	*TRGV9*	*TRGJP*	CALWEVQELGKKIKVF	94.51407
2	*TRGV9*	*TRGJP*	CALWEVQELDKKIKVF	0.254869
3	*TRGV9*	*TRGJP*	CALWEVQELSKKIKVF	0.242366
4	*TRGV9*	*TRGJP*	CALWGVQELGKKIKVF	0.166386
5	*TRGV9*	*TRGJP*	GALWEVQELGKKIKVF	0.142342
6	*TRGV9*	*TRGJP*	CALWEVQELGKKSKVF	0.142342
7	*TRGV9*	*TRGJP*	CALWELQELGKKIKVF	0.123107
8	*TRGV9*	*TRGJP*	CALWEVKELGKKIKVF	0.118298
9	*TRGV9*	*TRGJP*	CVLWEVQELGKKIKVF	0.114451
10	*TRGV9*	*TRGJP*	CAVWEVQELGKKIKVF	0.089445
11	*TRGV9*	*TRGJP*	CALWEVRELGKKIKVF	0.083674
12	*TRGV9*	*TRGJP*	RALWEVQELGKKIKVF	0.082712
13	*TRGV9*	*TRGJP*	CALWEVQELGKKIKVV	0.076942

ranking	*TRDV*	*TRDJ*	CDRJ	%reads
1	*TRDV2*	*TRDJ1*	CACDPLLGDTPRYTDKLIF	90.05091
2	*TRDV2*	*TRDJ1*	CACEPLLGDTPRYTDKLIF	0.330323
3	*TRDV2*	*TRDJ1*	CACDPLLGDMPRYTDKLIF	0.264663
4	*TRDV2*	*TRDJ1*	CACDPLLGDTPGYTDKLIF	0.259612
5	*TRDV2*	*TRDJ1*	CACDPLLGDTTRYTDKLIF	0.236378
6	*TRDV2*	*TRDJ1*	CACDPLLGGTPRYTDKLIF	0.210114
7	*TRDV2*	*TRDJ1*	CACDQLLGDTPRYTDKLIF	0.204053
8	*TRDV2*	*TRDJ1*	CACDTLLGDTPRYTDKLIF	0.192941
9	*TRDV2*	*TRDJ1*	CDCDPLLGDTPRYTDKLIF	0.1889
10	*TRDV2*	*TRDJ1*	CARDPLLGDTPRYTDKLIF	0.182839
11	*TRDV2*	*TRDJ1*	CVCDPLLGDTPRYTDKLIF	0.164656
12	*TRDV2*	*TRDJ1*	CACDPILGDTPRYTDKLIF	0.162636
13	*TRDV2*	*TRDJ1*	CACDPLLGDKPRYTDKLIF	0.153545

14	*TRGV9*	*TRGJP*	YALWEVQELGKKIKVF	0.073094
15	*TRGV9*	*TRGJP*	SALWEVQELGKKIKVF	0.073094
16	*TRGV9*	*TRGJP*	CASWEVQELGKKIKVF	0.073094
17	*TRGV9*	*TRGJP*	CALWEVQELGKKIKVC	0.069247
18	*TRGV9*	*TRGJP*	CALWEVQELGKRIKVF	0.068286
19	*TRGV9*	*TRGJP*	CALWEVQELGKKIKGF	0.063477
20	*TRGV9*	*TRGJP*	CALWEVQELGKKIEVF	0.063477
21	*TRGV9*	*TRGJP*	WALWEVQELGKKIKVF	0.062515
22	*TRGV9*	*TRGJP*	CAWWEVQELGKKIKVF	0.061553
23	*TRGV9*	*TRGJP*	CALWEVQELGKKIKVL	0.061553
24	*TRGV9*	*TRGJP*	CDLWEVQELGKKIKVF	0.05963
25	*TRGV9*	*TRGJP*	CALWEVQELGKEIKVF	0.056744
26	*TRGV9*	*TRGJP*	CTLWEVQELGKKIKVF	0.051936
27	*TRGV9*	*TRGJP*	CALWEVQELGKKIRVF	0.051936
28	*TRGV9*	*TRGJP*	CALWEVQGLGKKIKVF	0.050974
29	*TRGV9*	*TRGJP*	CALWEVQELGKKNKVF	0.050012
30	*TRGV9*	*TRGJP*	CAMWEVQELGKKIKVF	0.04905

14	*TRDV2*	*TRDJ1*	CACDPLLGDTPRYTDKRIF	0.151524
15	*TRDV2*	*TRDJ1*	CACDPRLGDTPRYTDKLIF	0.148494
16	*TRDV2*	*TRDJ1*	WACDPLLGDTPRYTDKLIF	0.137382
17	*TRDV2*	*TRDJ1*	CACGPLLGDTPRYTDKLIF	0.133341
18	*TRDV2*	*TRDJ1*	CACDPLMGDTPRYTDKLIF	0.117179
19	*TRDV2*	*TRDJ1*	CACDPLLGDTPKYTDKLIF	0.105057
20	*TRDV2*	*TRDJ1*	CACDPLLGDTQRYTDKLIF	0.103037
21	*TRDV2*	*TRDJ1*	CACDPLLRDTPRYTDKLIF	0.101016
22	*TRDV2*	*TRDJ1*	CACDPLLGDTPRYTNKLIF	0.100006
23	*TRDV2*	*TRDJ1*	CTCDPLLGDTPRYTDKLIF	0.096976
24	*TRDV2*	*TRDJ1*	CACDPLRGDTPRYTDKLIF	0.088894
25	*TRDV2*	*TRDJ1*	CGCDPLLGDTPRYTDKLIF	0.086874
26	*TRDV2*	*TRDJ1*	CAYDPLLGDTPRYTDKLIF	0.086874
27	*TRDV2*	*TRDJ1*	CACDPLLGDTPRYTDKLIL	0.078793
28	*TRDV2*	*TRDJ1*	CACNPLLGDTPRYTDKLIF	0.072732
29	*TRDV2*	*TRDJ1*	CACDPLLGNTPRYTDKLIF	0.071722
30	*TRDV2*	*TRDJ1*	CACDPLLEDTPRYTDKLIF	0.070711

31	*TRGV9*	*TRGJP*	CALWEVQELGKKIKVY	0.045203	31	*TRDV2*	*TRDJ1*	YACDPLLGDTPRYTDKLIF	0.068691
32	*TRGV9*	*TRGJP*	CALWEVQELGKKIKLF	0.041356	32	*TRDV2*	*TRDJ1*	CASDPLLGDTPRYTDKLIF	0.067681
33	*TRGV9*	*TRGJP*	CALREVQELGKKIKVF	0.041356	33	*TRDV2*	*TRDJ1*	CACDPLLGDTPRYNDKLIF	0.066671
34	*TRGV9*	*TRGJP*	CALWEVQELGKKINVF	0.040394	34	*TRDV2*	*TRDJ1*	RACDPLLGDTPRYTDKLIF	0.06465
35	*TRGV9*	*TRGJP*	CALWEVQELCKKIKVF	0.039433	35	*TRDV2*	*TRDJ1*	CACDPLLGDTPRYTGKLIF	0.057579
36	*TRGV9*	*TRGJP*	CALWEMQELGKKIKVF	0.039433	36	*TRDV2*	*TRDJ1*	CACDPLLGDTPRYTDKLSF	0.057579
37	*TRGV9*	*TRGJP*	CALWEVQKLGKKIKVF	0.037509	37	*TRDV2*	*TRDJ1*	CSCDPLLGDTPRYTDKLIF	0.055559
38	*TRGV9*	*TRGJP*	CALWKVQELGKKIKVF	0.036547	38	*TRDV2*	*TRDJ1*	CACDPLLGDTPRYTEKLIF	0.054549
39	*TRGV9*	*TRGJP*	CALWEVQEFGKKIKVF	0.035585	39	*TRDV2*	*TRDJ1*	CACDPLLGDTPRDTDKLIF	0.054549
40	*TRGV9*	*TRGJP*	CALWEVQELGKKIKIF	0.034624	40	*TRDV2*	*TRDJ1*	CACDPLLGDAPRYTDKLIF	0.054549
41	*TRGV9*	*TRGJP*	CGLWEVQELGKKIKVF	0.033662	41	*TRDV2*	*TRDJ1*	CAWDPLLGDTPRYTDKLIF	0.052528
42	*TRGV9*	*TRGJP*	CALWEVEELGKKIKVF	0.0327	42	*TRDV2*	*TRDJ1*	CACDPLLGDTPRYIDKLIF	0.052528
43	*TRGV9*	*TRGJP*	CALWEVQEWGKKIKVF	0.031738	43	*TRDV2*	*TRDJ1*	CACDPLLGDTLRYTDKLIF	0.052528
44	*TRGV9*	*TRGJP*	CALLEVQELGKKIKVF	0.031738	44	*TRDV2*	*TRDJ1*	CAGDPLLGDTPRYTDKLIF	0.050508
45	*TRGV9*	*TRGJP*	CALCEVQELGKKIKVF	0.031738	45	*TRDV2*	*TRDJ1*	CACDPLLGDTPRYTDNLIF	0.050508
46	*TRGV9*	*TRGJP*	CALWEGQELGKKIKVF	0.030777	46	*TRDV2*	*TRDJ1*	CACDPLLGDTPRYTDKPIF	0.049498
47	*TRGV9*	*TRGJP*	CALWEVQELRKKIKVF	0.028853	47	*TRDV2*	*TRDJ1*	CACDPLLGDTPRYTDKLVF	0.049498

48	*TRGV9*	*TRGJP*	CALWEAQELGKKIKVF	0.028853
49	*TRGV9*	*TRGJP*	CALWEVQELGKKIQVF	0.02693
50	*TRGV9*	*TRGJP*	CSLWEVQELGKKIKVF	0.025968

48	*TRDV2*	*TRDJ1*	CACDPLLGDTPRYTDKHIF	0.045457
49	*TRDV2*	*TRDJ1*	CACDPLQGDTPRYTDKLIF	0.044447
50	*TRDV2*	*TRDJ1*	CACDPLLGDTSRYTDKLIF	0.043437

Table S3. Primers for RT-PCR to detect undifferentiated marker genes, related to Figure S1B

GAPDH	forward	AGCCACATCGCTCAGACAC
	reverse	GCCCAATACGACCAAATCC
SeV	forward	GGATCACTAGGTGATATCGAGC
	reverse	ACCAGACAAGAGTTTAAGAGATATGTATC
OCT3/4	forward	GACAGGGGGAGGGGAGGAGCTAGG
	reverse	CTTCCCTCCAACCAGTTGCCCCAAAC
SOX2	forward	GGGAAATGGGAGGGGTGCAAAAGAGG
	reverse	TTGCGTGAGTGTGGATGGGATTGGTG
NANOG	forward	TGAACCTCAGCTACAAACAG
	reverse	TGGTGGTAGGAAGAGTAAAG

Table S4. Primers for genomic PCR of TCR gene rearrangement, related to Figure S1E.

TCRG	forward	*Vγ9*	CGGCACTGTCAGAAAGGAATC
	reverse	*TCRG-JP1.JP2*	GAAGTTACTATGAGCTTAGTCCCTT
		TCRG-JP	AAGCTTTGTTCCGGGACCAAATAC
		TCRG-J1.J2	TACCTGTGACAACAAGTGTTGTTC
TCRD	forward	*Vδ2*	ATACCGAGAAAAGGACATCTATG
	reverse	*JD1*	GTTCCACAGTCACACGGGTTC
		JD2	GTTCCACGATGAGTTGTGTTC
		JD3	CTCACGGGGCTCCACGAAGAG

Table S5. Antibodies used in flowcytometry and immunofluorescent staining

Antibodies		
anti-human CD34-APC	Biolegend	Clone:561, Cat:343607
anti-human CD43-PE	eBiosciences	Clone:84-3C1, Cat:12-0439
anti-human CD45RA-FITC	eBiosciences	Clone:HI100, Cat:11-0458
anti-human CD27-PE	eBiosciences	Clone:O323, Cat:12-0279
anti-human CD25-PE	eBiosciences	Clone:BC96, Cat:12-0259
anti-human CD7-FITC	eBiosciences	Clone:4H9, Cat:17-0078
anti-human CD5-PE	eBiosciences	Clone:VCHT2, Cat:12-0059
anti-human CD3-PC7	eBiosciences	Clone:UCHT1, Cat:25-0038
anti-human αβTCR-FITC	eBiosciences	Clone:WT31, Cat:11-9955
anti-human γδTCR-PE	eBiosciences	Clone:B11, Cat:12-9959
anti-human TCRγ9-FITC	Beckman Coulter	Clone:IMMU360, Cat:IM1463
anti-human GranzymeB-PE	eBiosciences	Clone:GB11, Cat:12-8899
anti-human Perforin-FITC	Biolegend	Clone:dG9, Cat:308103
anti-human Interferon γ-PC7	Biolegend	Clone:4S.B3, Cat:502527
anti-human CD56-PE	eBiosciences	Clone:TULY56, Cat:12-0566-41
anti-human CD335-PE	Biolegend	Clone:9E2, Cat:331907
anti Oct3/4	BD	Clone:40-Oct3, Cat:611202
anti Nanog	R&D Systems	Polyclonal, Cat:AF1997
anti SOX2	abcam	Polyclonal, Cat:ab97959
anti SOX17	R&D Systems	Polyclonal, Cat:AF1924
anti α-SMA	DAKO	Clone:1A4. Cat:M0851
anti βIII-tubulin	Millipore	Clone:TU20, Cat:MAB1637
anti TCRγδ	eBiosciences	Clone:B1.1, Cat:14-9959-82
anti NKG2D	eBiosciences	Clone:1D11, Cat:14-5878-82

SUPPLEMENTAL EXPERIMENTAL PROCEDURE

Establishment of human induced pluripotent stem cell lines from human γδT cell

The generation of γδT-derived hiPSC clone 121-3 was performed as previously described (Watanabe et al., 2018) with slight modifications. Peripheral blood mononuclear cells (PBMCs) were collected from healthy volunteers after obtaining their informed consent. First, PBMCs were separated from whole blood using a BD Vacutainer blood collection tube and stimulated with 5 μM Zoledronic acid in RPMI 1640 medium (Nacalai Tesque, #30264-56) supplemented with 10% fetal bovine serum (Sigma-Aldrich, F7524), 100 IU/mL IL-2 (Imunace, Shionogi Pharmaceuticals), 10 μM 2-mercaptoethanol (Gibco, 21985-023), 50 U/mL penicillin and 50 μg/mL streptomycin (Life Technologies, 15140-122). Stimulated PBMCs were suspended in 100 μl medium with Cytotune-iPS 2.0 (ID pharma, 69000-41) at an MOI of 2 and cultivated in a 96-well plate. Twenty-four hours later, the cells were plated on a 6-well plate pre-coated with i-Matrix-511 silk (Nippi, 892 024). After transduction, Stemfit medium (Ajinomoto, AK02N) was changed every two days. From day 21, we started to pick up and expand colonies.

The experiment was approved by the institutional review board of Kobe University Graduate School of Medicine.

iPSC culture

We cultured iPSC lines according to a previously described method (Miyazaki et al., 2017). Passaging was performed once a week. After aspirating supernatant and PBS(-) washing, cells were dissociated with 0.5× TrypLE select (1× TrypLE select [Thermo Fisher, A1285901] diluted 1:1 with 0.5 mM EDTA/PBS[-]) and seeded with StemFit medium with 10 μM Y-27632 (WAKO, 034-24024) and iMatrix-511 silk (0.167 $\mu g/cm^2$) in un-coated usage. After 24 hours, the medium was refreshed with StemFit medium without Y-27632. The cells were maintained at 37°C under 5% CO_2.

To examine pluripotency, the conventional iPSC clone 201B7, which was established from fibroblasts, was used as a positive control

Feeder cell culture

The feeder cell line: OP9/N-DLL1 was purchased from Riken Cell Bank (RCB2927). OP9/N-DLL1 cells were maintained in αMEM (Thermo Fisher, 11900-016) supplemented with 20% fetal bovine serum, 50 U/ml penicillin, 50 μg/ml streptomycin and 2 mM L-glutamine (Life technologies, 25036-081). Media was changed every other day. Passaging was done once a week. After PBS(-) washing, cells were dissociated with 0.25% Trypsin (Gibco, 15090046) and incubated at 37°C for 10 minutes. Cells were collected by pipetting and filtered using a 35 μm cell strainer. After centrifugation at 800 rpm for 4 minutes, cells were suspended in fully refreshed medium and seeded at density of 5.0×10^4 cells/well (12-well plate)

for HPC co-culture and 2.0×10^5 cells/60 mm dish for maintaining culture.

Culture of cancer cells

The Jurkat and Huh-7 cancer cell lines were obtained from Riken Bio Resource Center (Tsukuba, Ibaraki, Japan). The SW480 cancer cell line was obtained from American Type Culture Collection ([ATCC] Manassas, VA). Jurkat cells (Riken BRC, RCB0806) were cultured in RPMI 1640 containing 10% FBS and were passaged every 3 days. The Huh-7 (Riken BRC, RCB1366) and SW480 (ATCC, CCL-228) cell lines were cultured in DMEM (Nacalai tesque, 08458-45) containing 10% FBS. They were passaged every 3–5 days.

RT-PCR

To test the expression of pluripotent genes in γδT cell-derived iPSC clones, total RNA was extracted using TRIzol reagent (Life Technologies, 15596026) and treated with a Turbo DNA-free kit (Life Technologies, AM1907) to remove genomic DNA contamination. Total RNA was reverse transcribed to cDNA using a PrimeScript II 1st Strand Synthesis Kit (Takara, 2690A) with oligo-dT primers according to the manufacturer's instructions. The cDNA was subjected to PCR with a Takara Ex Taq PCR kit (Takara, RR001A). As positive control, 46A1s3, which was established in our previous report, was used (Watanabe et al., 2018). The primer sequences of the RT-PCR are listed in Table S3.

Analysis of TCRG and TCRD gene rearrangement

To analyze the rearrangement of the TCRG and TCRD gene regions, genomic DNA was extracted using PCI solution and subjected to PCR with a Takara Ex Taq PCR kit. The primer sequences are listed in Table S4.

***In vitro* spontaneous differentiation via embryoid body formation.**

For embryoid body (EB) formation, undifferentiated iPSCs were dissociated into single cells, re-suspended in Primate ES medium (Reprocell, RCHEMD001) containing 20 µM Y-27632, and seeded onto low-cell-adhesion 96-well spindle-bottom plates at a density of 1.0×10^4 cells per well. After 7 days of culture, the EBs were transferred to gelatin-coated 24-well plates and cultured in the same medium for another 7 days. The differentiated cells were immunostained with antibodies listed in table S5.

Immunofluorescent staining

Cells were fixed on a culturing plate with 4% paraformaldehyde for 15–30 minutes at room temperature. After PBS(-) washing, cells were permeabilized and blocked with 1% BSA/PBS(-) containing 0.3% TritonX-100 and 5% donkey serum for 1 hour at room temperature. Then cells were incubated with

primary antibodies overnight at 4°C. The cells were rewashed, then incubated with secondary antibody for 1 hour at room temperature. Fluorescence images were obtained using a BZ-X700 microscope (Keyence).

Karyotype analyses

The Q-band karyotype analysis for γδT-iPSC was performed at Chromocenter Inc. (Yonago, Japan).

Repertoire analysis

CD3+γδTCR+cells from iγδTs (121-3 derivatives) were stimulated with HMBPP and IL-2 for 10 days from day 30, and sorted on BD FACS melody at day 40. PBγδTs were stimulated for two weeks and not sorted. Total RNA was extracted using TRIzol reagent and treated with a Turbo DNA-free kit. cDNA was synthesized and amplified for TCRγ or TCRδ genes with the ligation of a universal adaptor to the leader sequence of variable regions. Sequencing was performed using an illumina MiSeq. More than 1×10^5 total read sequences were analyzed at Repertoire Genesis (Osaka, Japan)

The amino acid sequence reads with a frequency of <1% were omitted from the subsequent analysis as this assay does not guarantee reliability for such low-frequency sequences.

Time-lapse imaging

GFP- Huh-7 cells were generated with piggyBac Mammalian Expression Vector containing CometGFP and puromycin-resistant gene (PJ509-02: ATUM). At day -1, tumor cells were seeded at density of 2×10^5 cells in a 35 mm dish. At day 0, 4×10^5 effector cells in 1 mL of γδT stimulating medium were added. Serial phase contrast images were captured at intervals with a microscope (Bio Studio: Nikon engineering).

Cytotoxic assay

Jurkat cells were collected into tubes and centrifuged at 300×g for 4 minutes. After PBS(-) washing, cells were suspended with buffer-added CFSE solution (Basic cytotoxicity: immunochemistry, #970) and incubated at room temperature for 15 min. We added FBS containing medium, centrifuged, PBS(-) washing was performed. Target cells were counted and suspended with fresh medium at density of 5.0×105 cells/mL. Target cell suspension (100 μL) was seeded onto a 96-well U-bottom plate and 100 μL of effector cell suspension in γδT stimulating medium was added at several concentrations. As a negative control, 100 μL of γδT stimulating medium without effector cells was added. After co-culturing at 37°C overnight, we added 7-AAD solution into the well and performed FCM.

The SW480 cells were incubated with CFSE for 15 min before co-culture. For adherent cells, at day -1,

tumor cells were seeded at density of 2×10^5 cells in a 35 mm dish. At day 0, 4×10^5 effector cells in 1 mL of γδT stimulating medium were added. They were assessed after 12 hours (Huh-7) or 16 hours (SW480) co-culture.

Serial phase contrast images were captured at intervals with a microscope (Bio Studio: Nikon engineering). The areas of tumor cells were quantified using the Image J software program (ImageJ ver 1.51k, NIH) and the areas of surviving cells with or without iγδTs were calculated after correcting with the value before co-culture (0 h) as follows:

$$\frac{\text{Area of tumor cells (12 h or 16 h, i}\gamma\delta\text{T)/Area of tumor cells (0 h, i}\gamma\delta\text{T)}}{\text{Area of tumor cells (12 h or 16 h, no effector)/Area of tumor cells (0 h, no effector)}}$$

= X%/100%

Real-time cytotoxicity assay (xCELLigence assay)

The cytolytic potential of isolated iγδT cells was analyzed in a real-time cytotoxicity assay with an xCELLigence RTCA system (Agilent, 380601030). Huh-7 cells (1×10^4) were seeded in each well of a 96-well E-Plate. After one day, iγδT, PBγδT or PBNK cells were added at an E:T ratio of 2:1. Cell viability was monitored every 15 min for 12 h. Cell indexes (CIs) were normalized to the CI of the time-point when effector cells were added and specific lysis was calculated in relation to the control cells lacking any effector cells. In the case of floating K562 cells, cells were seeded on pre-coated wells with an xCELLigence immunoassay kit anti-CD71 (Agilent, 8100017). If needed, the wells were added with 20 μg/mL anti-human TCRγδ Ab (clone B1.1, eBioscience) or 20 μg/mL anti-human NKG2D Ab (clone 1D11, eBioscience).

Flow cytometry, fluorescence-activated cell sorting

Cells were filtered using a 35 μm cell strainer and incubated with antibodies diluted in FACS buffer on 4°C for 30 minutes with light shielding. After washing twice with FACS buffer (Nacalai Tesque, 25976–14), cells were re-suspended in FACS buffer and filtered with a 35 μm cell strainer again, analyzed on RF-500 (Sysmex) or sorted on FACS Aria III (BD Biosciences) or FACS Melody (BD Biosciences). Antibodies were listed in supplemental table S5.

FCM of cytoplasmic molecules

Brefeldin A was supplemented in a medium at a final concentration of 3 μg/mL. Effector cells were incubated at 37°C for 30 minutes, and co-cultured with target cells for 6 hours in a condition of Brefeldin A-containing medium. Then, mixed cells were collected, subjected to PBS (-) washing, and fixed with 4% paraformaldehyde for 20 minutes. The cells were rewashed, then permeabilized, blocked and incubated with antibodies diluted in 0.3% TritonX-100 in 1% human serum for 30 minutes on ice with light shielding. After washing, the cells were analyzed on an RF-500 flow cytometer.

Magnetic-activated cell sorting

Before the cytotoxicity assay, iγδTs and PBγδTs were purified with MACS CD3-MicroBeads (Miltenyi Biotec, 130-050-101) according to the manufacturer's instructions. PBNK cells were collected using an EasySep Human NK Cell isolation Kit (STEMCELL technologies, 18000).

Human Subjects

Peripheral blood mononuclear cells were isolated from normal healthy donors using protocols approved by the Kobe University Institutional Review Board. Informed consent was obtained from all subjects in accordance with the IRB protocols.

SUPPLEMENTAL REFERENCES

Miyazaki, T., Isobe, T., Nakatsuji, N., and Suemori, H. (2017). Efficient Adhesion Culture of Human Pluripotent Stem Cells Using Laminin Fragments in an Uncoated Manner. Sci Rep *7*, 41165. DOI: 10.1038/srep41165

Watanabe, D., Koyanagi-Aoi, M., Taniguchi-Ikeda, M., Yoshida, Y., Azuma, T., and Aoi, T. (2018). The Generation of Human gammadeltaT Cell-Derived Induced Pluripotent Stem Cells from Whole Peripheral Blood Mononuclear Cell Culture. Stem Cells Transl Med *7*, 34-44. DOI: 10.1002/sctm.17-0021
