# Supplementary material for: Re-generation of cytotoxic γδT cells with distinctive signatures from human γδT-derived iPSCs
Source: Stem Cell Reports. 2023 Mar 23;18(4):853–68. doi: 10.1016/j.stemcr.2023.02.010 (PMC10147660; doi:10.1016/j.stemcr.2023.02.010)
Supplement: Document S2. Article plus supplemental information [file mmc3.pdf]

Stem Cell Reports
Article
OPEN ACCESS

Re-generation of cytotoxic γδT cells with distinctive signatures from human γδT-derived iPSCs

Nobuyuki Murai,[1,2,3] Michiyo Koyanagi-Aoi,[1,2,4] Hiroto Terashi,[3] and Takashi Aoi[1,2,4,*]
[1]Division of Stem Cell Medicine, Graduate School of Medicine, Kobe University, Kobe, Hyogo, Japan
[2]Division of Advanced Medical Science, Graduate School of Science, Technology and Innovation, Kobe University, Kobe, Hyogo, Japan
[3]Division of Plastic Surgery, Graduate School of Medicine, Kobe University, Kobe, Hyogo, Japan
[4]Center for Human Resource Development for Regenerative Medicine, Kobe University Hospital, Kobe, Hyogo, Japan
*Correspondence: takaaoi@med.kobe-u.ac.jp
https://doi.org/10.1016/j.stemcr.2023.02.010

SUMMARY

For a long time, *ex vivo*-expanded peripheral-blood-derived γδT cell (PBγδT)-based immunotherapy has been attractive, and clinical trials have been undertaken. However, the difficulty in expanding cytotoxic γδT cells to an adequate number has been a major limitation to the efficacy of treatment in most cases. We successfully re-generated γδT cells from γδT cell-derived human induced pluripotent stem cells (iPSCs). The iPSC-derived γδT cells (iγδTs) killed several cancer types in a major histocompatibility complex (MHC)-unrestricted manner. Single-cell RNA sequencing (scRNA-seq) revealed that the iγδTs were identical to a minor subset of PBγδTs. Compared with a major subset of PBγδTs, the iγδTs showed a distinctive gene expression pattern: lower *CD2*, *CD5*, and antigen-presenting genes; higher *CD7*, *KIT*, and natural killer (NK) cell markers. The iγδTs expressed granzyme B and perforin but not interferon gamma (IFNγ). Our data provide a new source for γδT cell-based immunotherapy without quantitative limitation.

INTRODUCTION

Gamma delta T (γδT) cells attack various types of cancer cells in a major histocompatibility complex (MHC)-unrestricted manner (Wrobel et al., 2007). Therefore, peripheral blood-derived γδT cell (PBγδT)-based immunotherapy has received attention, and clinical trials have been undertaken (Kobayashi et al., 2010). Because the proportion of γδT cells in adult peripheral blood mononuclear cells amounts to only a few percent or less (Aljurf et al., 2002), γδT cells need to be expanded by stimulants *ex vivo* for clinical use (Khan et al., 2021). However, in most cases, the difficulty in expanding cytotoxic γδT cells derived from peripheral blood to an adequate number has been a major limitation to the efficacy of the treatment (Wada et al., 2014). Furthermore, PBMC-derived γδT cells can be expanded enough for them to be used for autologous adoptive immunotherapy, but not enough for them to be used for allogenic mass-produced immunotherapeutic modalities.

Induced pluripotent stem cell (iPSC) technology may be able to overcome these limitations and enable us to realize off-the-self allogenic γδT cell-based therapy, which has several advantages over autologous γδT cells therapy: (1) the *ex vivo* expansion rate of PBMC-derived γδT cells varies widely from donor individual to individual; thus, *ex vivo*-expanded autologous PBγδT cell therapy cannot be applied to all patients; (2) patients have to wait for the expansion of autologous cells but not for off-the shelf allogenic cells; and (3) an autologous approach would be associated with high costs. iPSCs have infinite proliferation ability: they show logarithmic growth for at least 100 days and ∼1,028-fold expansion during 100 days (∼10,000-fold/2 weeks) (Nakagawa et al., 2014). To generate γδT cells from human iPSCs (hiPSCs), the favorable cell of origin of the iPSC is a γδT cell, because T cell receptor (TCR) gene rearrangement is theoretically retained throughout the process of reprogramming and differentiation. Indeed, αβT cells differentiated from αβT cell-derived hiPSCs reportedly retained parental αβTCR gene rearrangement (Nishimura et al., 2013; Vizcardo et al., 2013), and re-generated αβT cells from iPSCs showed an antigen-specific cytotoxicity to cancer cells (Nishimura et al., 2013).

Previously we successfully established hiPSC lines from peripheral blood-derived γδT cells with a simple and clinically applicable method (Watanabe et al., 2018). These γδT cell-derived iPSCs (γδT-iPSCs) were demonstrated to be able to differentiate into CD34(+)CD43(+) hematopoietic progenitor cells. However, it has not yet been clarified whether the γδT-iPSCs can differentiate into γδT cells that can kill various types of cancer in an MHC-unrestricted manner. In a previous report by Zeng et al. (2019), the authors reported the generation of "mimetic γδT cells" endowed with natural killer (NK) receptors from γδT cell-derived iPSCs and designated them as γδNKT cells. However, the cells do not match any type of physiologically existing, authentic, or *bona fide* lymphocyte, including γδT cells, and should be categorized as cells resulting from an aberrant characteristic of lymphocytes derived from iPSCs, or abnormal cells. Previous studies have demonstrated that such abnormal cells can be derived from pre-rearranged TCR-carrying pluripotent stem cells or hematopoietic progenitors, and such iPSCs reportedly gave rise to abnormal

This is an open access article under the CC BY license (http://creativecommons.org/licenses/by/4.0/).

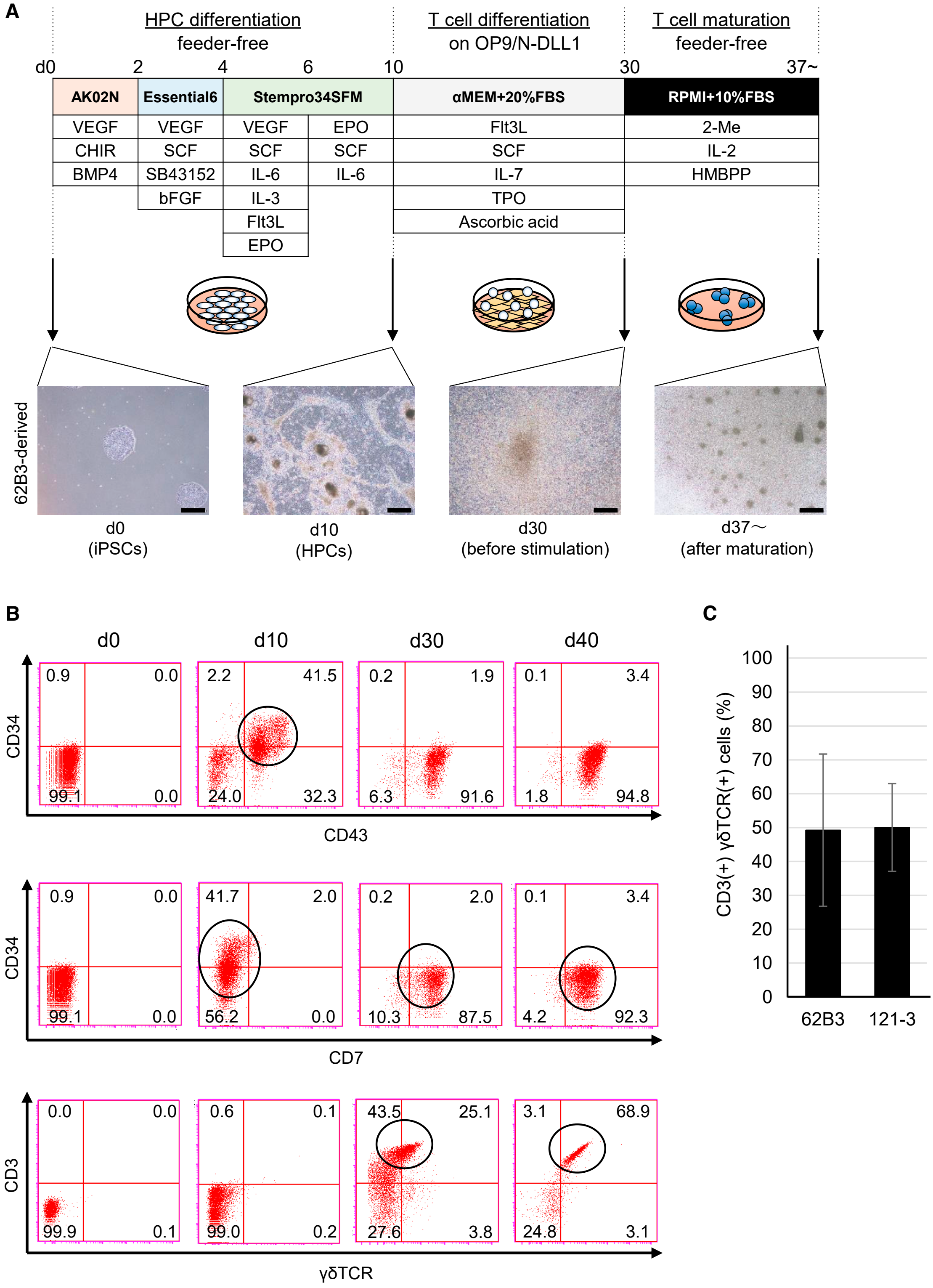

(legend on next page)

T cells expressing TCR, an NK cell marker NK1.1, and CD8αα (Vizcardo et al., 2018). Similarly, the resultant cells generated by Zeng et al. also express TCR, NK cell molecules, and CD8αα, suggesting the possibility that the cells might be mere "abnormal T cells" that had previously been known to be derived from pre-rearranged TCR-carrying pluripotent stem cells through unphysiological processes but not cells with any potential clinical utility.

Accordingly, if the γδT-iPSCs could differentiate into γδT cells, whether the molecular signatures of the re-generated resultant γδT cells from the γδT-iPSCs are identical to some subset of authentic γδT cells or absolutely artificial and unnatural cells should be revealed.

In this study, we successfully re-generated γδT cells from γδT-iPSCs. The iPSC-derived γδT cells (iγδTs) exhibited cytotoxicity against several cancer cell lines in an MHC-unrestricted manner. We identified distinctive molecular signatures of iγδTs and clarified that the iγδTs were identical to a minor subset of *ex vivo*-expanded PBγδT cells. Our data provide a new source for γδT cell-based immunotherapy without quantitative limitations.

RESULTS

Re-differentiation of γδT-iPSCs into γδT cells

For re-differentiation into γδT cells, we used two γδT-derived hiPSC lines from different donors: 62B3, which was established in our previous report (Watanabe et al., 2018), and 121-3, which was newly established in this study. We confirmed that both iPSC clones expressed undifferentiated markers (NANOG, OCT3/4, and SOX2) at protein levels (Figure S1A) and mRNA levels (Figure S1B) and that the Sendai virus vector used for the introduction of reprogramming factors had been removed (Figure S1B). An *in vitro* embryoid body (EB)-mediated differentiation experiment showed that they could differentiate into three germ layers (Figure S1C). In a Q-band analysis, karyotype abnormality was not observed (Figure S1D). Genomic PCR to examine the rearrangement at the *TCRG* and *TCRD* gene locus showed Vγ9-to-JP and Vδ2-to-JD1 recombination (Figure S1E). These data verified that the two lines (62B3 and 121-3) are γδT-derived iPSCs (γδT-iPSC) that carry Vγ9Vδ2-TCR genes.

Next, we re-differentiated these γδT-iPSCs into γδT cells according to previously reported protocols (Kutlesa et al., 2009; Watanabe et al., 2018) with slight modifications shown in Figure 1A. On day 10, we confirmed the induction of cells positive for both CD34 and CD43 (Figure 1B upper panels), the subset of which was shown to be hematopoietic progenitor cells (HPCs) (Timmermans et al., 2009). At this time point, no cells expressed CD7, CD3, or γδTCR (Figure 1B middle and lower panels). From day 10, the derivatives of γδT-iPS were co-cultured with OP9/N-DLL1 feeder cells, which have been commonly used for differentiation of HPCs into T lymphocytes (Kutlesa et al., 2009; Schmitt et al., 2004). Thereafter, the expression of CD34 gradually became negative, and cells positive for CD7, a pre-lymphoid and mature T cell marker (Ohishi et al., 2002; Timmermans et al., 2009), increased (Figure 1B middle panels).

On day 30, the expression of CD3 was clearly positive, while the expression of γδTCR was still weak (Figure 1B lower panels). To differentiate not only nonadherent differentiated cells but also more immature cells adhering to the feeder (Jing et al., 2010), we collected all cells, including feeder cells, and transferred them into a feeder-free dish. We then started γδTCR stimulation with (E)-4-Hydroxy-3-methyl-but-2-enyl diphosphate (HMBPP) (Figure 1A), which is a metabolite in a non-mevalonate pathway and which is known to activate Vγ9Vδ2 T cells (Nerdal et al., 2016). Although some feeder cells adhered to the new dish, they peeled off after several days (data not shown). Seven to 10 days after the start of γδTCR stimulation, we found cell aggregations with phase-contrast microscopy (Figure 1A), and most of the cells became clearly positive for both CD3 and γδTCR (Figure 1B lower panels), suggesting the maturation of the cells to γδT cells progressed. We confirmed reproducibility of the differentiation to CD3(+) γδTCR(+) cells from two γδT-iPSC lines (Figure 1C). We named the resultant cells iγδTs. At day 40 of differentiation, we obtained up to 3×10^5 iγδT cells from 2×10^3 iPSCs. Even after the induction of iγδT, CD3-negative cells still existed. Although it was unclear what type of cells the CD3-negative cells were, they were at least negative for CD56 and CD335, which are known markers of NK cells (Figure S2A).

Monoclonal γδTCR expression in iγδTs

Next, we examined whether the iγδTs expressed a monoclonal γδTCR, as theoretically expected. In contrast to CD3-positive cells in peripheral blood mononuclear cells

Figure 1. Re-differentiation of γδT-iPSCs into γδT cells

(A) Schematic diagram of the protocol for differentiation from γδT-iPSC into γδT cells is shown in the upper panel. Representative phase-contrast images of iPSCs (day 0) and iPSC derivatives (day 10, day 30, and day 37∼) are shown in the lower panels. Scale bars, 500 μm.

(B) Cell surface markers were analyzed by flow cytometry on days 0, 10, 30, and 40. The circle in scattergram indicates HPCs at day 10, immature T cells at day 30, and mature T cells at day 40.

(C) The proportions of CD3(+)γδTCR(+) cells in the derivatives of γδT-iPSCs (n = 4 independent experiments, mean ± SD).

(PBMCs) stimulated with HMBPP for 1 week being composed of both γδTCR-positive and αβTCR-positive cells, CD3-positive iγδTs contained no αβTCR-positive cells (Figure 2A). Genomic PCR to detect *TCRG* and *TCRD* genes showed that Vγ9-to-JP and Vδ2-to-JD1 recombination in γδT-iPSCs was retained in iγδTs (Figure 2B). Furthermore, we performed an analysis of the TCR γ and TCR δ repertoire of CD3(+) γδTCR(+) cells sorted from the iγδTs as well as HMBPP-stimulated PBγδTs. The results of the amino acid sequences in the CD3R lesion, which guarantee reliability only for amino acid sequences with a frequency of more than 1%, demonstrated that PBγδTs consisted of more than 25 clones (Figure 2C right panels; Table S1), whereas iγδTs consisted of only a single clone (Figure 2C left panels; Table S2)).

These data indicated that we successfully re-generated monoclonal γ9δ2 T cells via γδT-iPSCs.

Cytotoxicity of iγδTs against cancer cell lines

A key advantage of γδT cells for cancer immunotherapy is that one type of γδT cell is applicable for various types of cancer in a human leukocyte antigen (HLA)-unrestricted manner (Wrobel et al., 2007). We therefore evaluated the toxicity of the iγδTs against four types of cancer cell lines, including two non-solid tumor (Jurkat cells [an acute T cell leukemia cell line] and K562 cells [a chronic myelogenous leukemia cell line]) and two solid tumors (Huh-7 cells [a hepatocellular carcinoma cell line] and SW480 cells [a colorectal adenocarcinoma cell line]). We confirmed that these cell lines have different HLA types from the two iPSC lines used in this study (Table 1).

First, we labeled Jurkat cells with carboxyfluorescein diacetate succinimidyl ester (CFSE) as target cells, co-cultured with the iγδTs as effector cells at an effector (E):target (T) ratio of 2:1 and stained these cells with 7-aminoactinomycin-D (7-AAD) to identify dead cells, followed by flow cytometry (FCM). The no-effector condition showed that only approximately 5% of Jurkat cells were dead (Figure 3A left panel). In contrast, the derivatives of iPSCs showed obvious cytotoxicity; co-culture with effector cells for 1 day resulted in cell death of approximately half of the target cells, regardless of whether or not CD3 and γδTCR co-positive cells were sorted (Figure 3A middle and right panels). Accordingly, we decided to use unsorted iγδTs as effector cells for the subsequent cytotoxicity assays to avoid cellular damage caused by sorting. We confirmed the reproducibility of cytotoxicity using a γδT-iPSC line, 62B3-derived iγδTs, generated in eight independent differentiation experiments. All the experiments showed the cytotoxicity of iγδTs toward Jurkat cells (Figure 3B), although the magnitude of efficacy varied from experiment to experiment. Another γδT-iPSC line, 121-3-derived iγδTs, also showed cytotoxicity toward Jurkat cells (Figure S3A).

To assess the cytotoxicity of iγδTs toward solid tumor cells, we observed the co-culture of 62B3 γδT-iPSC line-derived iγδTs with GFP-Huh-7 cells by time-lapse imaging. After 12 h of co-culture, the areas of GFP-Huh-7 cells decreased to 28.7%, 30.1%, and 64.5% of those in no-effector control culture in three independent experiments (Figures 3C and 3D). Moreover, we were able to catch iγδTs coming into contact with GFP-Huh-7 cells and peeling off as time went on (Video S1). The change in the area of GFP-Huh-7 cells each hour is shown in Figure S3B. Similarly, co-culture of CFSE-labeled SW480 and the derivatives of a γδT-iPSC line, 62B3, resulted in a decrease in the areas of SW480 cells to 38.6%, 64.4%, and 66.0% of those of the no-effector control at 16 h in three independent experiments (Figures 3E and 3F). Another γδT-iPSC line, 121-3-derived iγδTs, also showed cytotoxicity toward Huh-7 cells (Figure S3C) and SW480 cells (Figure S3D).

Next, to confirm the cytotoxicity of purified-iγδTs, we co-cultured CD3-MACS-purified iγδT cells and tumor cells and quantified the tumor toxicity at an E:T ratio of 2:1 at 12 h using xCELLigence. Against Huh-7 cells, the purified iγδT and PBγδT cells showed no significant difference in cytotoxicity (Figure 3G). Moreover, because γδT cells reportedly express NK receptor molecules, such as NKG2D (Rincon-Orozco et al., 2005), we performed a cytotoxicity assay using K562 cells, which are known to be killed by NK cells, as target cells. iγδT cells and peripheral blood-derived NK (PBNK) cells showed similar cytotoxic activity against K562 cells, with no significant difference (Figure 3H).

These data demonstrated that the iγδTs have cytotoxicity for at least four different types of cancer cells in an HLA-unrestricted manner. Moreover, we were able to catch iγδTs coming into contact with GFP-Huh-7 cells and peeling off as time went on (Video S1).

Mode of action of iγδTs

We next performed several experiments to obtain insight into the mode of action of the iγδTs. The co-culture of iγδTs and Jurkat cells at various E:T ratios showed the dose-dependent cytotoxic effects of iγδTs (Figures 4A and S3E). Notably, even at an E:T ratio of only 0.25:1, cytotoxicity was clearly observed and reached a plateau at 2:1, while a previous report on iPSC-derived T cells showed their cytotoxicity toward lymphoma cell lines at E:T ratios of greater than 20:1 (Themeli et al., 2013). To evaluate the persistence of cytotoxicity, we co-cultured iγδTs and Jurkat cells at a low E:T ratio of 0.5:1 for up to 4 days. The results showed that cytotoxicity increased in a time-dependent manner and lasted for at least 4 days (Figures 4B and S3F).

Next, we investigated the mechanism by which iγδTs exhibit cytotoxicity. Both blocking antibodies for γδTCR

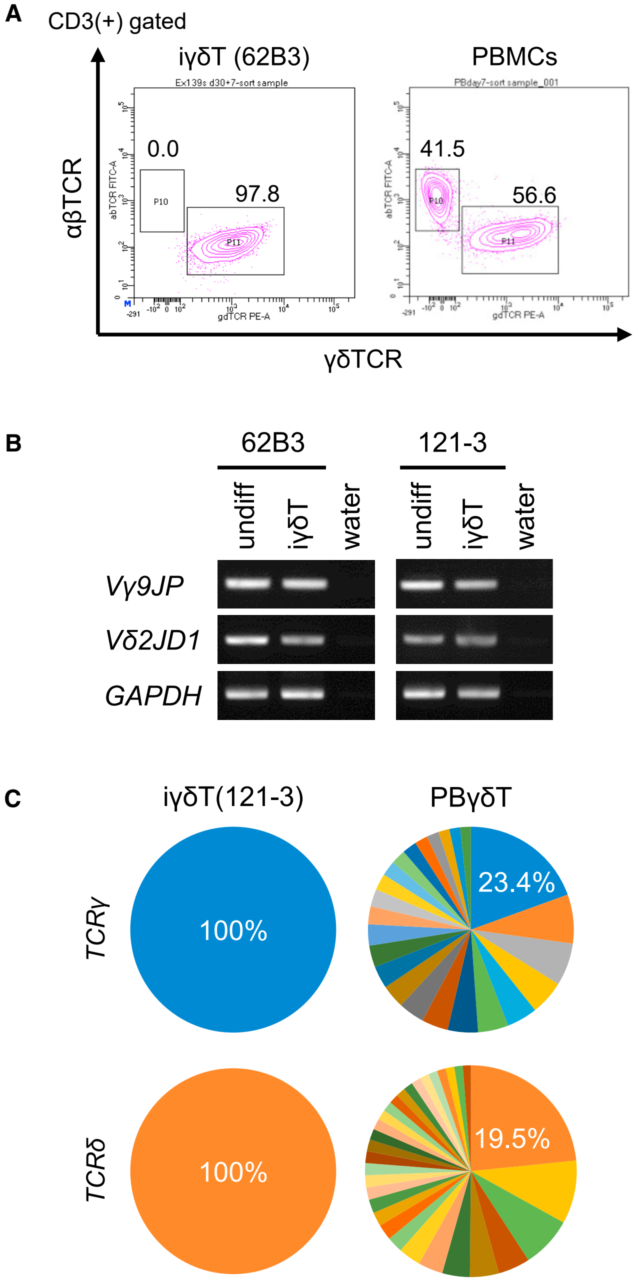

Figure 2. Validation of iγδTs

(A) Flow cytometry to detect the expression of γδT cell receptor (γδTCR) and αβTCR in CD3(+) sorted 62B3-derived iγδTs (left) and PBMCs (right). Both were stimulated with HMBPP and IL-2 for 7 days.

(B) Genomic PCR to detect TCR gene rearrangement in undifferentiated original iPSC (undiff) and CD3(+) γδTCR(+) sorted iγδTs derived from the iPSC lines (iγδT).

(C) The circle graph indicates the variance of the VDJ sequence in iγδTs and PBγδTs. The number indicates the proportion of the dominant repertoire.

and NKG2D reduced the cytotoxicity of the purified iγδTs, suggesting that the iγδTs recognize tumor cells by both γδTCR and NKG2D (Figure 4C). Perforin, granzyme B, and interferon gamma (IFNγ) are reported to play important roles in the cytotoxicity of γδT cells (O'Neill et al., 2020). To determine whether iγδTs release these factors, iγδTs re-generated from the 62B3 γδT-iPSC line and Jurkat cells were co-cultured with the addition of Brefeldin A, which blocks the transportation of proteins to the Golgi bodies and induces the accumulation of proteins in the ER. The iγδTs were pre-labeled with a CD3 antibody before co-culture to distinguish iγδTs from Jurkat cells. At 4 h of co-culture, the cells were fixed with 4% PFA and analyzed by FCM. Granzyme B and perforin, which are expressed in cytotoxic T cells (Brandes et al., 2009; Voskoboinik et al., 2015), were expressed in both the iγδTs and PBγδT cells (Figure 4D), suggesting that the iγδTs, like authentic γδT, directly attach to and attack tumor cells with lytic granules carried by secretory lysosomes.

Notably, no iγδTs were positive for IFNγ. In contrast, most granzyme B-positive cells in PBγδT cells were positive for IFNγ (Figure 4D). This finding raised the question as to whether iγδTs have other distinct molecular signatures from PBγδTs.

Comparison of cell surface markers in iγδTs and PBγδTs

We compared the cell surface markers of iγδTs with PBγδT cells by FCM (Figure 4E). Both derivatives of the two γδT-iPSC lines (62B3 and 121-3) and PBMCs were stimulated with HMBPP and interleukin (IL)-2 for 10 days (iγδTs, from day 30 of differentiation; PBγδTs, from the first day of culture). In iγδTs, >90% of CD3(+) cells were positive for CD7, whereas <80% of CD3(+) cells were positive for CD7 in PBγδTs (Figure 4E uppermost panels). Moreover, CD3(+) cells were also positive for CD5 in <20% of iγδTs and >90% of PBγδT cells (Figure 4E second row of panels). In TCRγ9(+) cells, <50% of the cells were positive for CD25 (IL2RA) in iγδTs, whereas >85% of PBγδTs were positive (Figure 4E third row of panels).

In general, T cells are divided into four subsets of naive or memory phenotypes corresponding to the CD45RA and CD27 expression patterns (Berard and Tough, 2002). Despite stimulation during the same period, most iγδTs showed a CD45RA(+) CD27(−) phenotype. In contrast, PBγδTs existed in all four subsets (Figure 4E bottom panels). CD45RA(+) CD27(−) γδT cells have been reported to correspond to terminally differentiated effector memory T cells, which have a low expansion capacity (Odaira et al., 2016). Although the significance of the expression patterns of CD45RA and CD27 in γδT cells remains unclear, the expression patterns of these molecules also differ between iγδTs and PBγδTs.

Table 1. HLA phenotyping of iPSC and cancer cell lines

	HLA-A		HLA-B		HLA-C		HLA-DRB1	
62B3	02:01	24:02	40:01	54:01	01:02	03:04	04:03	04:05
121-3	24:02	31:01	35:01	52:01	04:01	12:02	09:01	13:02
Jurkat	03:01	–	07:02	35:03	04:01	07:02	07:01	15:01
SW480	02:01	24:02	07:02	15:18	07:02	07:04	01:03	13:01
K562	31:01	–	40:01	50:01	03:04	05:01	03:04	–

scRNA-seq reveals distinct populations of γδT cells in PBγδT cells and iγδTs

γδT cells have been reported to have various subtypes (Lawand et al., 2017; Li et al., 2020; Wu et al., 2017) and it was found that iγδTs and PBγδTs show different expression patterns in the bulk state from the verification of cell surface markers. To examine whether there are differences in the subtypes of γδT cells between iγδTs and PBγδT cells, we performed targeted single-cell RNA sequencing (scRNA-seq) of the following three types of cells: (1) freshly isolated PBMCs (no stimulation and no sorting). (2) PBγδT cells; PBMCs were expanded with HMBPP *in vitro* for 7 days and CD3(+) γδTCR(+) cells were sorted. (iii) iγδTs; differentiated cells from γδT-iPSC clone 62B3 (according to the protocol shown in Figure 1A) were stimulated with HMBPP for 6–12 days in three independent experiments and CD3(+) γδTCR(+) cells were sorted. Unsupervised clustering of three datasets (freshly isolated PBMCs, PBγδT cells, and one of three iγδT samples) identified six distinct cell clusters, which was shown by t-distributed stochastic neighbor embedding (t-SNE) (Figure 5A).

Cells in cluster 1 were characterized by the expression pattern of *TRDC*(+), *CD3E*(+), *CD4*(−), *CD8A*(+), and *CD8B*(−) (Figures 5B and S4A), and we categorized these as γδT subset 1. Cells in clusters 2 and 3 were characterized by the expression pattern of *TRDC*(+), *CD3E*(+), *CD4*(−) *CD8A*(−), and *CD8B*(−) (Figures 5B and S4A), and we categorized these as γδT subset 2 and γδT subset 3, respectively. Cells in cluster 4, which expressed either *CD4* or *CD8A*/*CD8B*, were categorized as αβT cells. These cells also expressed *SELL* (Figure S4B). Cells in cluster 5 were non-T cells, consisting of *MS4A1*+ B cells and *S100A9*+ monocytes (Figure S4C). Cells in cluster 6 partially expressed *TRDC* and *CD3E* (Figure 5B), but did not have other features, except that their expression of *LGALS1* was higher compared with cells in other clusters (Figure S4D). We categorized these as unknown.

The t-SNE distribution of each sample and the fraction of clusters in each sample are shown in Figures 5C and 5D, respectively. As expected, freshly isolated PBMCs were mostly occupied by αβT cells (cluster 4) and non-T cells (cluster 5) and contained 5.2%, 1%, and 1% of γδT subsets 1, 2, and 3, respectively (Figure 5D left bar graph). In PBγδT cells, γδT subset 2 accounted for approximately 70% of the total with 16.3% of γδT subset 1 and 1.7% of γδT subset 3 (Figure 5D middle bar graph). On the other hand, γδT subset 1 accounted for the majority (89.1%) in iγδTs. The rest was γδT subset 3 at 2.8%, and γδT subset 2 was completely absent (Figure 5D right bar graph). These results indicated that the major γδT subsets differ between PBγδT cells and iγδTs, and that cells similar to the major subset of iγδTs exist in PBγδT as a minor subset as well as in PBMCs.

To examine the expression of major immune-related genes in each γδT subset, we created dot plots for T cell-differentiation marker genes (Terstappen et al., 1992; Tydell et al., 2007), cytokine receptors (Caccamo et al., 2005; Corpuz et al., 2016; Sugamura et al., 1996), effector molecules (Fan and Zhang, 2005; O'Neill et al., 2020; Yang et al., 2020), NK cell-related genes (Yang et al., 2020), inhibitory receptors (Themeli et al., 2013), and MHC class I and II (Hurley, 2021; Karakikes et al., 2012; Yang et al., 2020) (Figure 5E). These data suggest that the expression levels of immune-related genes and the proportion of cells expressing them differed among the γδT subsets.

Differentially expressed genes in each γδT cell subset

Next, we extracted differentially expressed genes in each cluster against the rest of the clusters (e.g., cluster 1 vs. the mean of clusters 2–6). Cells in γδT subset 1, the main population of iγδTs, were enriched for NK cell-related genes (e.g., *CTSW*, *FCER1G*, *KLRC3*, *CD244*, *NKG7*, as well as the cytotoxic marker perforin coding gene [*PRF1*]). Cells in γδT subset 2, the dominant population of *in vitro*-expanded PBγδTs stimulated with HMBPP, expressed immune checkpoint inhibitory receptors (*PDCD1* [PD-1], *CTLA4*, and *LAG3*) (Figure 5E). The dominant population of iγδT were positive for the expression of these inhibitory receptor genes in cells in γδT subset 1, but the expression levels were lower than those in γδT subset 2. Antigen-presenting genes (*CD74*, *HLA-DQB1*, *HLA-DMA*, *HLA-DPA1*, *HLA-DRA*) and *IFNγ* and IFNγ-inducing genes (*IL12RB*, *IRF4*) (Xu et al., 2010; Yao et al., 2013) were expressed at higher levels compared with cells in other clusters. The

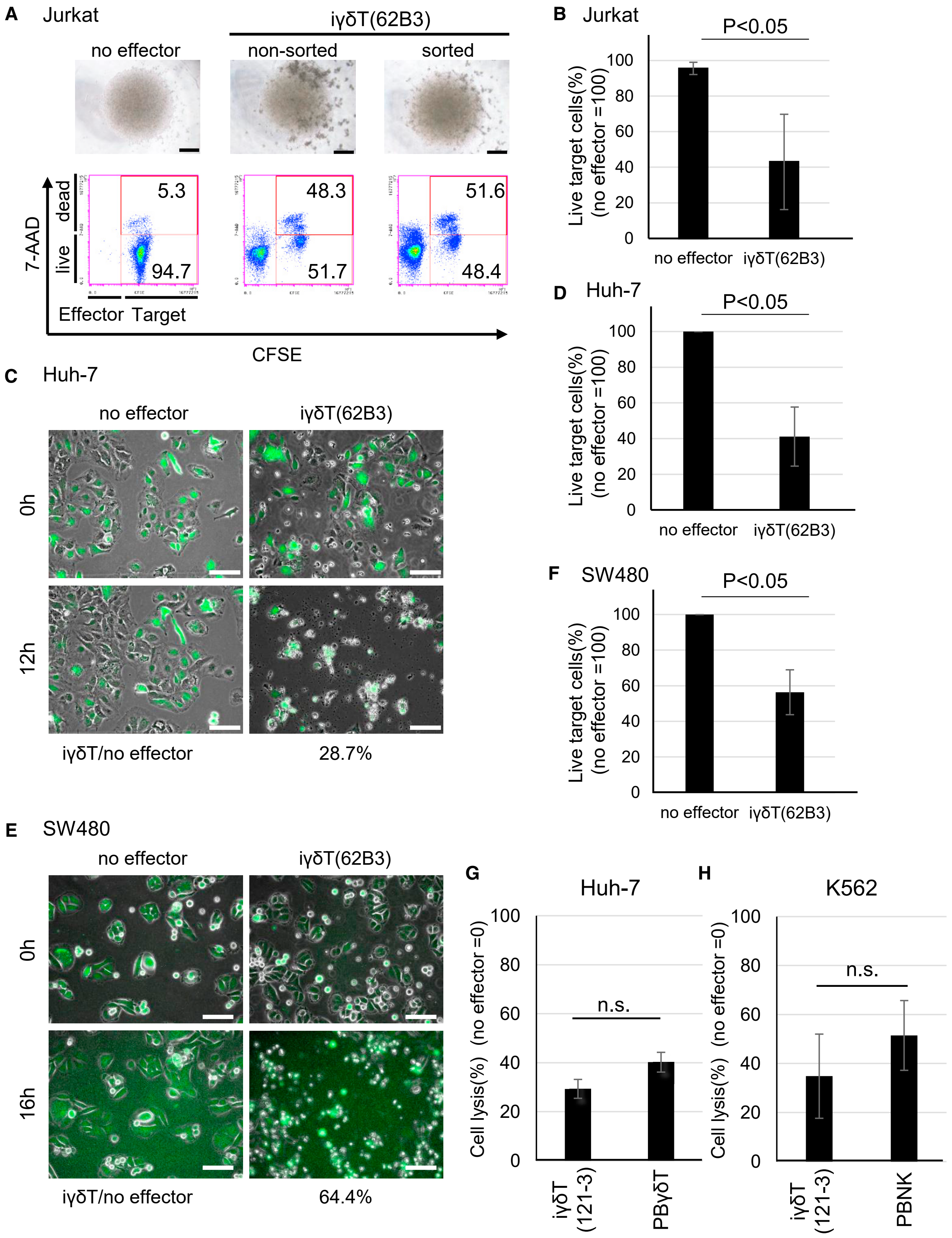

(legend on next page)

RORC expression was restricted to cells in γδT subset 3 (Figures 6A and 6B left panel). *IL-17A*, which was reported to be released from *RORC*+ γδT cells (Ben Hmid et al., 2020), was not expressed in cells in γδT subset 3 or in any of the other cells in this study (Figure 6B right panel). A violin plot showed that *KLRC3* and *LAG3* were specifically expressed in γδT subsets 1 and 2, respectively (Figure 6C). The expression of *KIT* was higher in the cells of γδT subsets 1 and 3 (Figures 6A and 6C). On the other hand, pan-T cell marker *CD2* and MHC class II molecule *HLA-DRA* were expressed in γδT subset 2, but not in γδT subsets 1 and 3 (Figures 6A and 6C).

In order to investigate the reproducibility of the iγδT data, the expression of these marker genes in scRNA-seq data of iγδTs prepared three times independently was shown by a heatmap. The similar expression patterns indicated that the gene expression of iγδTs was reproducible (Figure S4E).

Taken together, we successfully re-generated MHC-unrestricted cytotoxic γδT cells from iPSCs and clarified the distinctive molecular signatures of iPSC-derived γδT cells.

DISCUSSION

In the present study, we successfully re-generated CD3(+) γδTCR(+) cells from γδT cell-derived iPSCs. Although there have been reports of the re-generation of αβT cells from αβT cell-derived iPSCs (Maeda et al., 2016; Nishimura et al., 2013; Vizcardo et al., 2013), it was unclear whether a protocol similar to that for αβT cell differentiation from iPSCs could be applied to γδT cells, because the development process of γδT cells was reported to differ from that of αβT cells in several points. First, during fetal development, γδT cells precede αβT cells (Hayday, 2000). HPCs first differentiate into CD4/8 double-negative (DN) cells and then progress to CD4/8 double-positive (DP) cells (Seo and Taniuchi, 2016). While αβT cells differentiate from DP cells, γδT cells can differentiate from both DP and DN cells (Van Coppernolle et al., 2012). The weak and strong TCR signal strength received by DN cells favors αβT and γδT lineage development, respectively (Hayes et al., 2005). Furthermore, transcription factor Bcl11b-knockout mouse studies revealed that Bcl11b was essential for the differentiation of DN cells into αβT cells, but not necessary for differentiation into γδT cells (Ikawa et al., 2010), and these γδT cells without Bcl11b only show a CD5(−) phenotype (Hatano et al., 2017). The expression of Bcl11b and CD5 were low in our iγδTs, as shown in Figures 4E and 5E, suggesting that the development of iγδTs may be similar to that of CD5(−) γδ T cells *in vivo*.

With a scRNA-seq analysis, we revealed the distinct signatures of iγδTs from PBγδTs. They shared common clusters with a minor part of freshly isolated PBMCs and *ex vivo*-expanded PBγδTs, indicating that the cells

Figure 3. Cytotoxicity of iγδTs against cancer cell lines

(A) Phase-contrast images and dot plots for CFSE and 7-AAD staining of Jurkat cells after co-culture for 16 h with no-effector cells (left), whole iγδTs (non-sorted, middle) and CD3(+) γδTCR(+) sorted iγδTs (sorted, right) (E:T ratio = 2:1). Scale bars, 500 μm in upper panels. Flow cytometry was performed to determine the percentage of live (CFSE[+] and 7-AAD[−]) or dead (CFSE[+] and 7-AAD[+]) cells in target Jurkat cells.

(B) The proportions of live Jurkat cells after co-culture with or without 62B3-derived iγδTs for 1 day (n = 8 independent experiments, mean ± SD, two-tailed paired t test).

(C) Representative continuous images at 0 and 12 h displaying GFP-Huh-7 cells co-cultured with or without 62B3-derived iγδTs. The proportion of the GFP-positive area in target cells co-cultured with iγδTs for 12 h was 28.7% of the GFP-positive area of the target cells with no effector, which was set to 100%. See also Figure S3B. Scale bars indicate 100 μm.

(D) The relative proportion of live GFP-Huh-7 cells after co-culture with or without iγδTs was calculated according to the GFP-positive area. The proportion of the no-effector group at 12 h was set to 100%, as a control (n = 3 independent experiments, mean ± SD, two-tailed paired t test).

(E) Representative continuous images at 0 and 16 h displaying CFSE-stained SW480 cells co-cultured with or without 62B3-derived iγδTs. The proportion of the CFSE-positive area at 16 h in target cells co-cultured with iγδTs was 64.4% of the CFSE-positive area of the target cells with no effector, which was set to 100%. Scale bars indicate 100 μm.

(F) The relative proportion of live CFSE-stained SW480 cells after co-culture with or without iγδTs was calculated by CFSE-positive area. The proportion of the no-effector group at 16 h was set to 100%, as a control (n = 3 independent experiments, mean ± SD, two-tailed paired t test).

(G) Huh-7 cells were incubated with CD3(+) sorted iγδTs or PBγδTs at an E:T ratio of 2:1 and the cytotoxicity was determined using an xCELLigence RTCA system. The proportion of the no-effector group was set to 0%, as a control (n = 3 independent experiments, mean ± SD, two-tailed paired t test).

(H) K562 cells were incubated with CD3(+) sorted iγδT or PBNK cells, and the cytotoxicity was determined using an xCELLigence RTCA system. The proportion of the no-effector group was set to 0%, as a control (n = 3 independent experiments, mean ± SD, two-tailed paired t test).

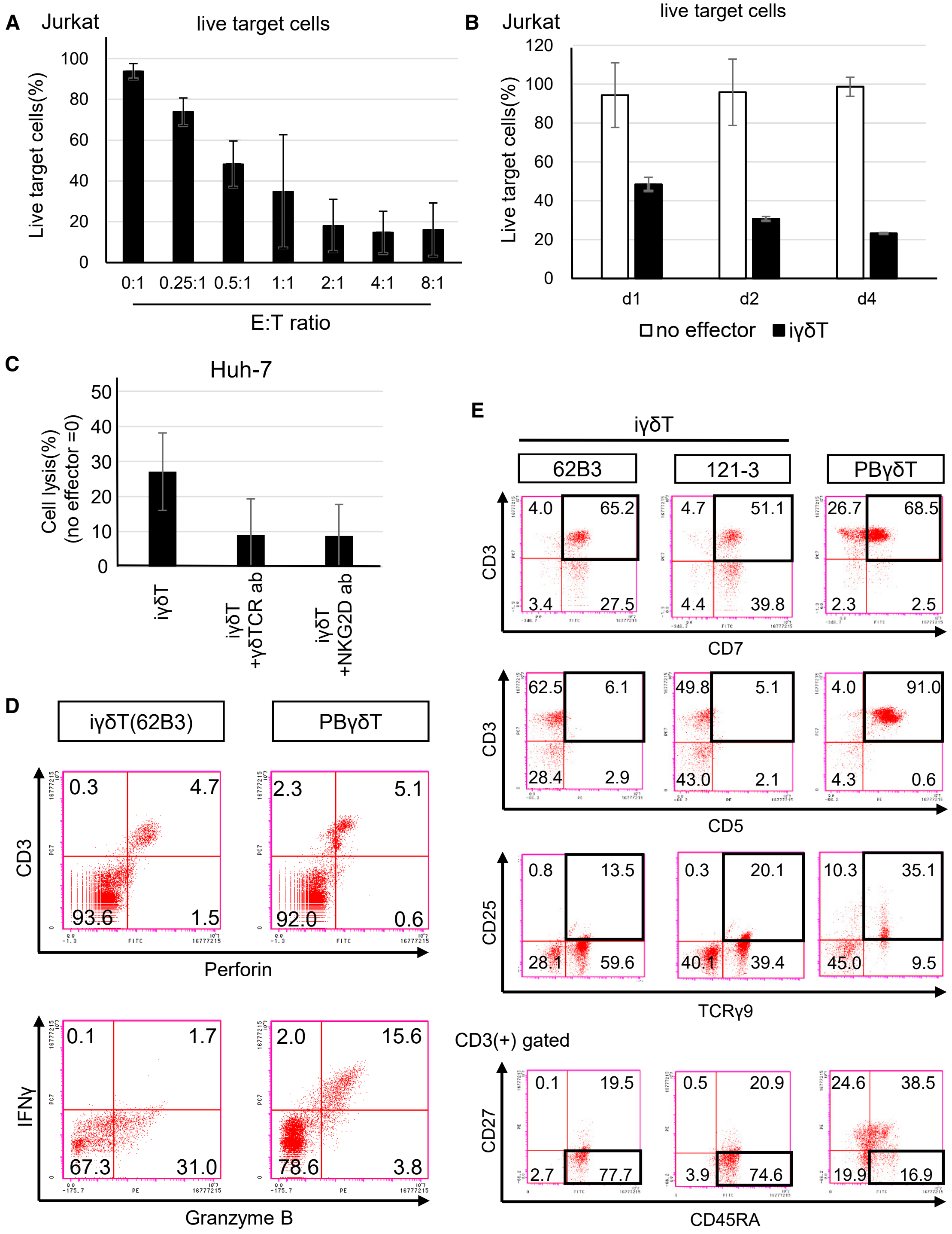

Figure 4. Modes of action of iγδTs and comparison with PBγδTs

(A) The percentage of live Jurkat cells after co-culture with 62B3-derived iγδTs at the indicated E:T ratios for 1 day (n = 3 independent experiments, mean ± SD). See also Figure S3E.

(B) The time course analysis of live Jurkat cells co-cultured with (black bars) or without (white bars) 62B3-derived iγδTs at an E:T ratio of 0.5: 1 for 4 days (n = 3 independent experiments, mean ± SD). See also Figure S3F.

(legend continued on next page)

resembling the major population of iγδTs exist in adult PBMCs in nature but are not expandable with HMBPP stimulation, at least under the culture condition used in this study. Previously there have been many studies concerning the classification of human peripheral γδT cells. For example, the functions of γδT cells were reportedly separated into five subsets: IFN-γ-producing, antigen-presenting, follicular B helper, regulatory γδT, and IL-17-producing cells (Wu et al., 2017). One other group classified γδT cells according to the effects on tumor cells: anti-tumor or tumor promoting (Li et al., 2020). Another group divided γδT cells according to the expression of activation marker genes, such as CD16 (Braakman et al., 1992), CD69 (Cibrian and Sanchez-Madrid, 2017), and RORC (Ben Hmid et al., 2020). Our iγδTs did not fully correspond to any of these previously reported γδT cell types in postnatal peripheral blood, in terms of the gene expression patterns. Notably, in contrast to PBγδTs, the iγδTs were negative for CD2 and positive for CD7. A previous report showed that a human fetal thymus-derived γδT cell clone showed a CD2 (low) CD7(+) phenotype and low IFNγ secretion (Carding et al., 1990). Our iγδTs might correspond to γδT cells derived from the fetal thymus.

The cytotoxicity of our iγδTs, which showed distinctive molecular signatures, can be supported by some previous reports. The iγδTs were CD5(−), and CD5(−) γδT cells were reported to be more cytotoxic than CD5(+) γδT cells (Srour et al., 1990). In addition, approximately three-quarters of the iγδTs showed a terminally differentiated T cell phenotype: CD45RA(+)/CD27(−). Terminally differentiated γ9δ2T cells reside in inflamed tissue, where they display an immediate effector function (Dieli et al., 2003) and exert higher cytotoxicity and lower IFNγ production compared with other subsets in terms of the CD45RA and CD27 expression pattern (Caccamo et al., 2005). Together, molecular mechanisms that link the molecular signatures of our iγδTs and their function should be clarified in future studies.

NK cell-related markers were expressed in iγδTs. A subset of authentic γδT cells reportedly expressed NK cell-related genes and recognize target cells by a similar mechanism to NK cells. In addition, it is reported that mimetic-γδ NKT cells, which expressed low T cell-related genes and high NK cell-related genes, were induced from iPSCs (Zeng et al., 2019). The shared NK cell markers support tumor direct recognition by γδT cells in an MHC-unrestricted manner (Wrobel et al., 2007). This NK-related gene expression may be responsible for the cytotoxicity of iγδTs.

We herein demonstrated that our iγδTs were completely negative for αβTCR and that they killed tumor cells in an MHC-independent manner. The negative expression of αβTCR may reduce the risk of graft-versus-host disease (Radestad et al., 2014). For this reason, there were studies in which allogenic PBγδTs were used as carriers for chimeric antigen receptor T (CAR-T) (Rozenbaum et al., 2020) and TCR-T (Ichiki et al., 2020).

Several limitations of the present study should be addressed in our future studies. First, the induction efficiency of iγδTs was not satisfactory, and we have not clarified what CD3(−) cells existing after iγδTs induction were. Second, it should be evaluated whether or not the iγδTs attack non-cancer cells of KIR-ligand mismatch recipients. Third, the iγδT induction protocol established in this study used xenogenic serum and feeder cells, which are difficult for clinical applications. We are currently trying to generate iγδTs under feeder-free and serum-free conditions (data not shown). Our technologies will advance off-the-shelf γδT cell-based immune therapy.

EXPERIMENTAL PROCEDURES

Resource availability

Materials availability

This study did not generate new unique reagents.

Data and code availability

The accession number for the scRNAseq reported in this paper is GEO: GSE194072.

Differentiation to γδT cells from iPSCs

Seven days before induction, human γδT-iPSCs were seeded onto a six-well plate at a density of 2.0×10^3 cells and cultured in StemFit medium (Ajinomoto, AK02N).

On day 0, the medium was completely replaced by StemFit medium supplemented with 4 μM CHIR99021 (Tocris, 4423), 80 ng/mL BMP4 (R&D, 314-BP), and 80 ng/mL vascular endothelial growth factor (VEGF) (R&D, 293-VE). On day 2, the medium was replaced by Essential 6 medium (Thermo Fisher, A1516501) supplemented with 2 μM SB431542 (WAKO, 033-24631), 50 ng/mL bFGF (WAKO, 060-04543), 50 ng/mL SCF (R&D, 255-SC), and 80 ng/mL VEGF. On day 4, the medium was replaced by StemPRO-34 SFM (Thermo Fisher, 10639-011) supplemented with 2 mM L-glutamine, 50 ng/mL IL-3 (Peprotech, AF-200-03), 50 ng/mL IL-6 (R&D, 206-IL), 50 ng/mL FLT3L (R&D, 308-FK),

(C) The percentage cytolysis of Huh-7 cells after 12 h of co-culture with or without neutralizing antibodies (20 μg/mL). The percentage cytolysis of the no-effector group was set to 0%, as a control (n = 3 independent experiments, mean ± SD).
(D) Flow cytometry to detect cytotoxic molecules. PBγδTs and iγδTs were pre-incubated with Brefeldin A and co-cultured with Jurkat cells at the E:T ratio of 2:1 for 4 h. In the upper panels, effector cells were pre-labeled with a CD3 antibody before the start of co-culture.
(E) The expression of T cell-related markers were analyzed by flow cytometry in γδT-iPSC-derived iγδTs and PBγδTs. The cells were stimulated with HMBPP and IL-2 for 10 days and sorting was not performed.

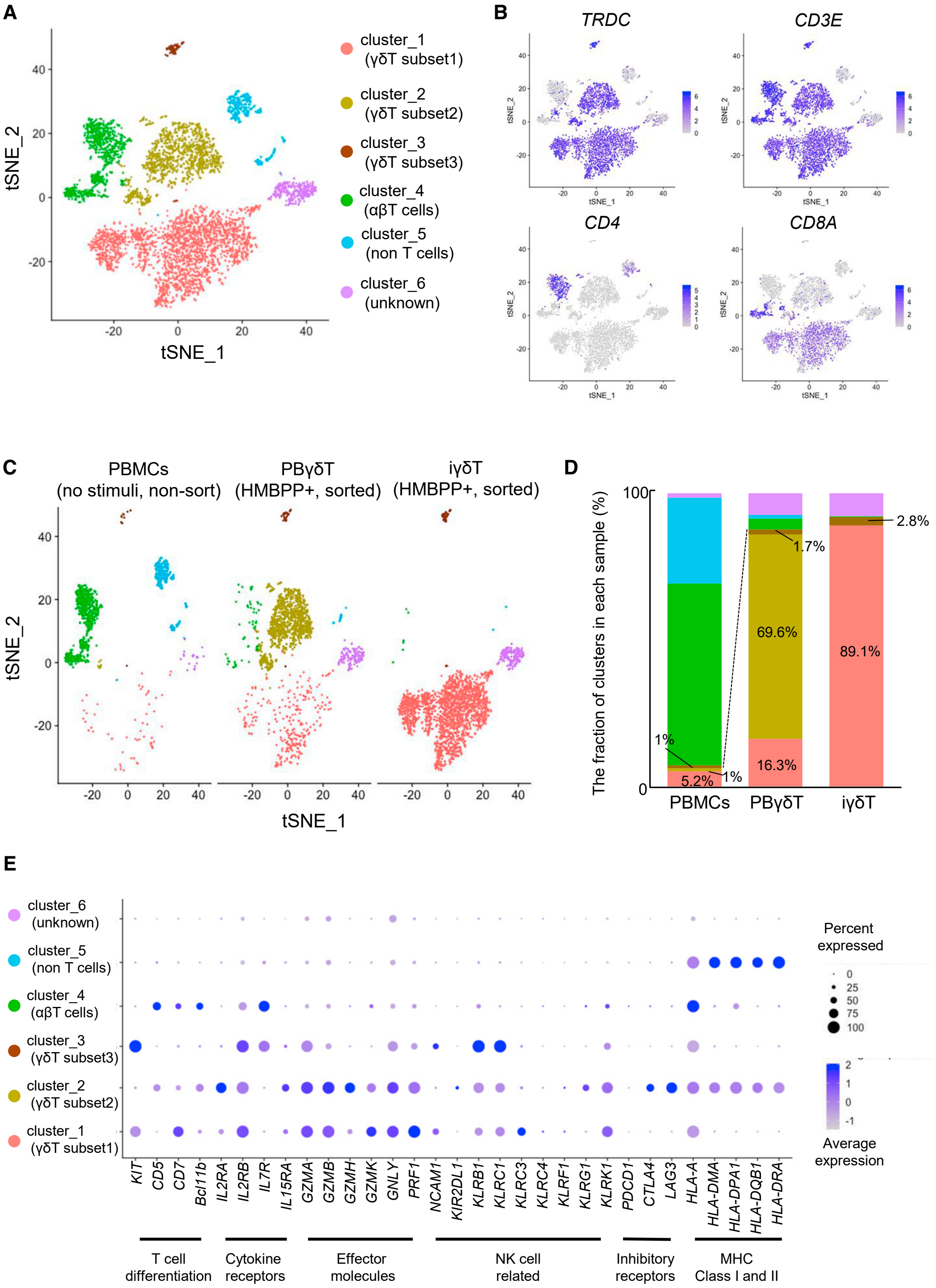

(legend on next page)

50 ng/mL SCF, 20 ng/mL VEGF, and 10 IU/mL EPO (ESPO, Kyowa Kirin).

On days 6 and 8, the medium was replaced with StemPRO-34 SFM supplemented with 2 mM L-glutamine, 50 ng/mL IL-6, 50 ng/mL SCF, and 10 IU/mL EPO.

On day 10, hematopoietic cells were transferred into wells co-cultured with feeder cells. Floating cells and supernatant were collected in the tube. Adhesive cells were dissociated with Accutase (Nacalai Tesque, 12679-54), and incubated at 37°C for 10 min. Supernatant was returned to the well, pipetted, and filtered using a 35-μm cell strainer. After centrifugation at 1,200 rpm for 4 min, cells were suspended in OP9 medium supplemented with 10 ng/mL SCF, 10 ng/mL TPO (Peprotech, AF-300-18), 5 ng/mL IL-7 (R&D, 207-IL), 5 ng/mL FLT3L, and 100 μg/mL L-ascorbic acid (Nacalai Tesque, 30264-56). Cells were reseeded to the same well and incubated at 37°C for 30 min. Without pipetting, supernatant and floating cells were transferred into a new well confluent with pre-seeded OP9/N-DLL1 cells.

On day 12, half of the medium was changed and cells were transferred into new wells with fresh OP9/N-DLL1 cells by vigorous pipetting. Then, half of the medium was changed every other day and cells were transferred onto fresh OP9/N-DLL1 cells every 6 days.

On day 30, we collected cells with Accutase, similarly to day 10. Cells were suspended with RPMI1640 medium supplemented with 10% FBS, 1 nM HMBPP (Cayman Chemical, 13580), 100 IU/mL IL-2 (Shionogi Pharmaceuticals, Imunace), and 10 μM 2-mercaptoethanol and seeded onto new plates in a feeder-free condition. Half of the medium was changed every other day. After more than a week of stimulation, cytotoxicity was analyzed.

scRNA-seq

The day before the targeted scRNA-seq analysis, CD3(+)γδTCR(+) cells were sorted on a BD FACS Aria III from PBγδTs and iγδTs that were stimulated with HMBPP for the indicated days as described above. To infer the origin of the sample, all cells were labeled with multiplex sample tags. Single-cell capture and cDNA library preparation were performed using a BD Rhapsody Single-Cell Analysis System with a BD Human Single-Cell Multiplexing Kit (BD Biosciences, #633781) and BD Human Immune Response Targeted Panel for Human (BD Biosciences, #633750), which contains 399 primer pairs, targeting 397 different genes, according to the manufacturer's recommendations. The concentration, size, and integrity of the resulting PCR products were assessed using a Qubit High-Sensitivity dsDNA Kit.

Sequencing was performed using an Illumina HiSeq X (Illumina, San Diego, CA) in Macrogen (Tokyo, Japan). Fastq files were uploaded to the Seven Bridges Genomics online platform. The obtained counts were adjusted by distribution-based error correction (DBEC), an error correction algorithm developed by BD Biosciences. DBEC data were then loaded into Seurat (version 4.0.4.). Cells were then clustered using a resolution of 0.03 and visualized by t-SNE. The Seurat functions FeaturePlot, DotPlot, DoHeatmap, and Vlnplot were used to visualize the gene expression with feature plot, dot plot, heatmap, and violin plot, respectively. Markers for a specific cluster against all remaining cells were found by using the Seurat function FindAllMarkers.

ScRNA-seq data have been deposited in GEO under accession number GSE194072.

Statistical analysis

Data are expressed as the mean ± SD. Differences between two groups were analyzed using a paired t test. Statistical analyses were performed using Microsoft Excel 2013 and EZR. p values of <0.05 were considered statistically significant.

SUPPLEMENTAL INFORMATION

Supplemental information can be found online at https://doi.org/10.1016/j.stemcr.2023.02.010.

AUTHOR CONTRIBUTIONS

Conceptualization, T.A; methodology, N.M; software, T.A. and M.K.-A; validation, M.K.-A.; formal analysis M.K.-A.; investigation, N.M.; resources, N.M.; writing – original draft, N.M.; writing – review & editing, T.A., M.K.-A.; supervision, T.A. and H.T.; project administration, T.A.

ACKNOWLEDGMENTS

We are grateful to all of the members of our laboratory for their scientific comments and valuable discussion. We also thank Yoko Matsuoka and Yukari Takatani for the administrative support. Naoya Hosono and Takehiro Jimbo supported the scRNA-seq analysis.

This work was supported by a grant for Research Center Network for Realization of Regenerative Medicine (16817073) from the Japan Agency for Medical Research and Development, AMED (T.A. and M.K.-A.), JSPS KAKENHI (18H02796; T.A.), Akira Sakagami Fund for Research and Education, Kobe University Graduate School of Medicine (T.A. and M.K.-A.), and Research Assistance Funds from Shinryokukai General Incorporated Association (T.A.).

CONFLICT OF INTERESTS

The authors declare no competing interests.

Figure 5. Clustering by single-cell RNA-seq

(A) Combined scRNA-seq analysis of freshly isolated PBMCs, PBγδTs, and iγδTs. t-SNE visualization showing six clusters.

(B) Feature plots show the expression of marker genes to define clusters. Blue indicates a high expression level; light gray indicates that the gene was not expressed. See also Figure S4A.

(C and D) Comparison of cluster distribution across three samples illustrated by split t-SNE (C) and a bar graph (D). Freshly isolated PBMCs, unstimulated and unsorted PBMCs; PBγδT, *in vitro* expanded with HMBPP and CD3(+) γδTCR(+) sorted peripheral blood-derived γδT cells; iγδT, *in vitro* expanded with HMBPP and CD3(+) γδTCR(+) sorted iγδTs.

(E) Dot plot showing the expression of immune-related genes for the cells in each cluster. Dot size represents the percentage of cells expressing the genes; color scale represents the gene expression level.

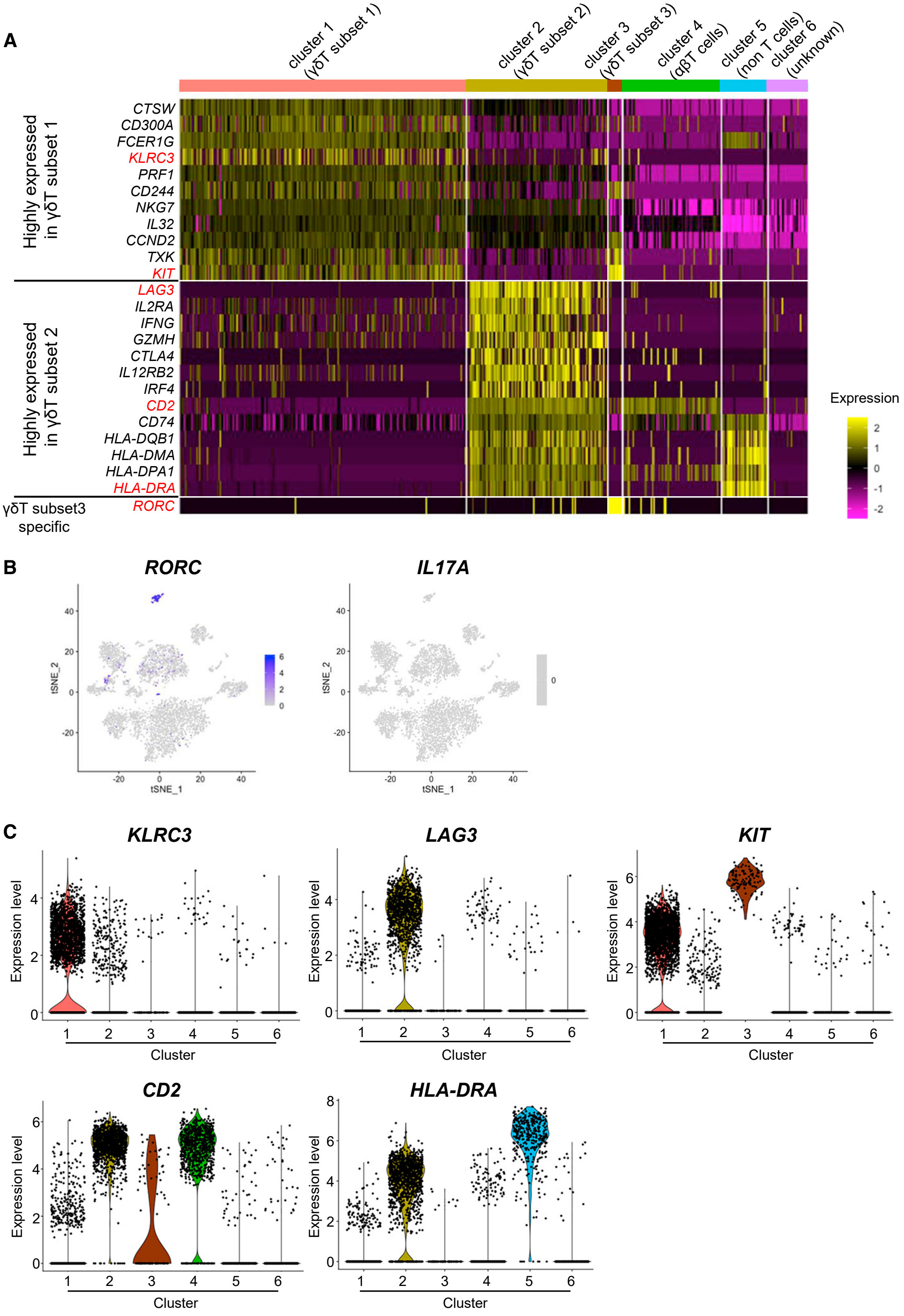

(legend on next page)

Received: May 27, 2022
Revised: February 24, 2023
Accepted: February 24, 2023
Published: March 23, 2023

REFERENCES

Aljurf, M., Ezzat, A., and O Musa, M. (2002). Emerging role of γδ T-cells in health and disease. Blood Rev. *16*, 203–206. https://doi.org/10.1016/s0268-960x(02)00029-2.

Ben Hmid, A., Selmi, O., Rekik, R., Lamari, H., Zamali, I., Ladeb, S., Safra, I., Ben Othman, T., Ben Romdhane, N., and Ben Ahmed, M. (2020). RORC overexpression as a sign of Th17 lymphocytes accumulation in multiple myeloma bone marrow. Cytokine *134*, 155210. https://doi.org/10.1016/j.cyto.2020.155210.

Berard, M., and Tough, D.F. (2002). Qualitative differences between naive and memory T cells. Immunology *106*, 127–138. https://doi.org/10.1046/j.1365-2567.2002.01447.x.

Braakman, E., van de Winkel, J.G., van Krimpen, B.A., Jansze, M., and Bolhuis, R.L. (1992). CD16 on human gamma delta T lymphocytes: expression, function, and specificity for mouse IgG isotypes. Cell. Immunol. *143*, 97–107. https://doi.org/10.1016/0008-8749(92)90008-d.

Brandes, M.n., Willimann, K., Romero, P., and Moser, B. (2009). Cross-presenting human gdT cells induce robust CD8+abT cell responses.pdf. Proc. Natl. Acad. Sci. USA *106*, 2307–2312.

Caccamo, N., Meraviglia, S., Ferlazzo, V., Angelini, D., Borsellino, G., Poccia, F., Battistini, L., Dieli, F., and Salerno, A. (2005). Differential requirements for antigen or homeostatic cytokines for proliferation and differentiation of human Vgamma9Vdelta2 naive, memory and effector T cell subsets. Eur. J. Immunol. *35*, 1764–1772. https://doi.org/10.1002/eji.200525983.

Carding, S.R., McNamara, J.G., Pan, M., and Bottomly, K. (1990). Characterization of gamma/delta T cell clones isolated from human fetal liver and thymus. Eur. J. Immunol. *20*, 1327–1335. https://doi.org/10.1002/eji.1830200619.

Cibrian, D., and Sanchez-Madrid, F. (2017). CD69: from activation marker to metabolic gatekeeper. Eur. J. Immunol. *47*, 946–953. https://doi.org/10.1002/eji.201646837.

Corpuz, T.M., Stolp, J., Kim, H.O., Pinget, G.V., Gray, D.H., Cho, J.H., Sprent, J., and Webster, K.E. (2016). Differential responsiveness of innate-like IL-17- and IFN-gamma-Producing gammadelta T cells to homeostatic cytokines. J. Immunol. *196*, 645–654. https://doi.org/10.4049/jimmunol.1502082.

Dieli, F., Poccia, F., Lipp, M., Sireci, G., Caccamo, N., Di Sano, C., and Salerno, A. (2003). Differentiation of effector/memory Vdelta2 T cells and migratory routes in lymph nodes or inflammatory sites. J. Exp. Med. *198*, 391–397. https://doi.org/10.1084/jem.20030235.

Fan, Z., and Zhang, Q. (2005). Molecular mechanisms of lymphocyte-mediated cytotoxicity. Cell. Mol. Immunol. *2*, 259–264.

Hatano, S., Murakami, T., Noguchi, N., Yamada, H., and Yoshikai, Y. (2017). CD5(-)NK1.1(+) gammadelta T Cells that develop in a Bcl11b-independent manner participate in early protection against infection. Cell Rep. *21*, 1191–1202. https://doi.org/10.1016/j.celrep.2017.10.007.

Hayday, A.C. (2000). γδ cells : a right time and a right place for a conserved third way of protection. Annu. Rev. Immunol. *18*, 975–1026. https://doi.org/10.1146/annurev.immunol.18.1.975.

Hayes, S.M., Li, L., and Love, P.E. (2005). TCR signal strength influences alphabeta/gammadelta lineage fate. Immunity *22*, 583–593. https://doi.org/10.1016/j.immuni.2005.03.014.

Hurley, C.K. (2021). Naming HLA diversity: a review of HLA nomenclature. Hum. Immunol. *82*, 457–465. https://doi.org/10.1016/j.humimm.2020.03.005.

Ichiki, Y., Shigematsu, Y., Baba, T., Shiota, H., Fukuyama, T., Nagata, Y., So, T., Yasuda, M., Takenoyama, M., and Yasumoto, K. (2020). Development of adoptive immunotherapy with KK-LC-1-specific TCR-transduced gammadeltaT cells against lung cancer cells. Cancer Sci. *111*, 4021–4030. https://doi.org/10.1111/cas.14612.

Ikawa, T., Hirose, S., Masuda, K., Kakugawa, K., Satoh, R., Shibano-Satoh, A., Kominami, R., Katsura, Y., and Kawamoto, H. (2010). An essential developmental checkpoint for production of the T cell lineage. Science *329*, 93–96.

Jing, D., Fonseca, A.V., Alakel, N., Fierro, F.A., Muller, K., Bornhauser, M., Ehninger, G., Corbeil, D., and Ordemann, R. (2010). Hematopoietic stem cells in co-culture with mesenchymal stromal cells–modeling the niche compartments in vitro. Haematologica *95*, 542–550. https://doi.org/10.3324/haematol.2009.010736.

Karakikes, I., Morrison, I.E., O'Toole, P., Metodieva, G., Navarrete, C.V., Gomez, J., Miranda-Sayago, J.M., Cherry, R.J., Metodiev, M., and Fernandez, N. (2012). Interaction of HLA-DR and CD74 at the cell surface of antigen-presenting cells by single particle image analysis. FASEB J. *26*, 4886–4896. https://doi.org/10.1096/fj.12-211466.

Khan, M.W.A., Otaibi, A.A., Sherwani, S., Alshammari, E.M., Al-Zahrani, S.A., Khan, W.A., Alsukaibi, A.K.D., Alouffi, S., and Khan, S.N. (2021). Optimization of methods for peripheral blood mononuclear cells isolation and expansion of human gamma delta T cells. Bioinformation *17*, 460–469. https://doi.org/10.6026/97320630017460.

Kobayashi, H., Tanaka, Y., Shimmura, H., Minato, N., and Tanabe, K. (2010). Complete remission of lung metastasis following

Figure 6. Extraction of genes with characteristic expression in each γδT cell subset

(A) A heatmap of genes that were highly expressed in γδT subset 1 (n = 11), γδT subset 2 (n = 13), and γδT subset 3 (n = 1) in the analyzed cells. Each column represents the gene expression profile of a single cell. The gene expression is color coded with a scale based on the *Z* score distribution, from low (purple) to high (yellow).

(B) Feature plots show the expression of marker genes. Blue indicates a high expression level; light gray indicates that the gene was not expressed.

(C) Violin plots showing the expression of selected genes from each cluster.

adoptive immunotherapy using activated autologous gammadelta T-cells in a patient with renal cell carcinoma. Anticancer Res. *30*, 575–579.

Kutlesa, S., Zayas, J., Valle, A., Levy, R.B., and Jurecic, R. (2009). T-cell differentiation of multipotent hematopoietic cell line EML in the OP9-DL1 coculture system. Exp. Hematol. *37*, 909–923. https://doi.org/10.1016/j.exphem.2009.05.002.

Lawand, M., Dechanet-Merville, J., and Dieu-Nosjean, M.C. (2017). Key features of gamma-delta T-cell subsets in human diseases and their immunotherapeutic implications. Front. Immunol. *8*, 761. https://doi.org/10.3389/fimmu.2017.00761.

Li, Y., Li, G., Zhang, J., Wu, X., and Chen, X. (2020). The dual roles of human gammadelta T cells: anti-tumor or tumor-promoting. Front. Immunol. *11*, 619954. https://doi.org/10.3389/fimmu.2020.619954.

Maeda, T., Nagano, S., Ichise, H., Kataoka, K., Yamada, D., Ogawa, S., Koseki, H., Kitawaki, T., Kadowaki, N., Takaori-Kondo, A., et al. (2016). Regeneration of CD8alphabeta T cells from T-cell-derived iPSC imparts potent tumor antigen-specific cytotoxicity. Cancer Res. *76*, 6839–6850. https://doi.org/10.1158/0008-5472.CAN-16-1149.

Nakagawa, M., Taniguchi, Y., Senda, S., Takizawa, N., Ichisaka, T., Asano, K., Morizane, A., Doi, D., Takahashi, J., Nishizawa, M., et al. (2014). A novel efficient feeder-free culture system for the derivation of human induced pluripotent stem cells. Sci. Rep. *4*, 3594. https://doi.org/10.1038/srep03594.

Nerdal, P.T., Peters, C., Oberg, H.H., Zlatev, H., Lettau, M., Quabius, E.S., Sousa, S., Gonnermann, D., Auriola, S., Olive, D., et al. (2016). Butyrophilin 3A/CD277-dependent activation of human gammadelta T cells: accessory cell capacity of distinct leukocyte populations. J. Immunol. *197*, 3059–3068. https://doi.org/10.4049/jimmunol.1600913.

Nishimura, T., Kaneko, S., Kawana-Tachikawa, A., Tajima, Y., Goto, H., Zhu, D., Nakayama-Hosoya, K., Iriguchi, S., Uemura, Y., Shimizu, T., et al. (2013). Generation of rejuvenated antigen-specific T cells by reprogramming to pluripotency and redifferentiation. Cell Stem Cell *12*, 114–126. https://doi.org/10.1016/j.stem.2012.11.002.

O'Neill, K., Pastar, I., Tomic-Canic, M., and Strbo, N. (2020). Perforins expression by cutaneous gamma delta T cells. Front. Immunol. *11*, 1839. https://doi.org/10.3389/fimmu.2020.01839.

Odaira, K., Kimura, S.N., Fujieda, N., Kobayashi, Y., Kambara, K., Takahashi, T., Izumi, T., Matsushita, H., and Kakimi, K. (2016). CD27(-)CD45(+) gammadelta T cells can be divided into two populations, CD27(-)CD45(int) and CD27(-)CD45(hi) with little proliferation potential. Biochem. Biophys. Res. Commun. *478*, 1298–1303. https://doi.org/10.1016/j.bbrc.2016.08.115.

Ohishi, K., Varnum-Finney, B., and Bernstein, I.D. (2002). The notch Pathway_Modulation of cell fate decisions in hematopoiesis. Int. J. Hematol. *75*, 449–459.

Radestad, E., Wikell, H., Engstrom, M., Watz, E., Sundberg, B., Thunberg, S., Uzunel, M., Mattsson, J., and Uhlin, M. (2014). Alpha/beta T-cell depleted grafts as an immunological booster to treat graft failure after hematopoietic stem cell transplantation with HLA-matched related and unrelated donors. J. Immunol. Res. *2014*, 578741. https://doi.org/10.1155/2014/578741.

Rincon-Orozco, B., Kunzmann, V., Wrobel, P., Kabelitz, D., Steinle, A., and Herrmann, T. (2005). Activation of V gamma 9V delta 2 T cells by NKG2D. J. Immunol. *175*, 2144–2151. https://doi.org/10.4049/jimmunol.175.4.2144.

Rozenbaum, M., Meir, A., Aharony, Y., Itzhaki, O., Schachter, J., Bank, I., Jacoby, E., and Besser, M.J. (2020). Gamma-delta CAR-T cells show CAR-directed and independent activity against leukemia. Front. Immunol. *11*, 1347. https://doi.org/10.3389/fimmu.2020.01347.

Schmitt, T.M., de Pooter, R.F., Gronski, M.A., Cho, S.K., Ohashi, P.S., and Zuniga-Pflucker, J.C. (2004). Induction of T cell development and establishment of T cell competence from embryonic stem cells differentiated in vitro. Nat. Immunol. *5*, 410–417. https://doi.org/10.1038/ni1055.

Seo, W., and Taniuchi, I. (2016). Transcriptional regulation of early T-cell development in the thymus. Eur. J. Immunol. *46*, 531–538. https://doi.org/10.1002/eji.201545821.

Srour, E.F., Leemhuis, T., Jenski, L., Redmond, R., Fillak, D., and Jansen, J. (1990). Characterization of normal human CD3+ CD5- and gamma delta T cell receptor positive T lymphocytes. Clin. Exp. Immunol. *80*, 114–121. https://doi.org/10.1111/j.1365-2249.1990.tb06450.x.

Sugamura, K., Asao, H., Kondo, M., Tanaka, N., Ishii, N., Ohbo, K., Nakamura, M., and Takeshita, T. (1996). The interleukin-2 receptor gamma chain: its role in the multiple cytokine receptor complexes and T cell development in XSCID. Annu. Rev. Immunol. *14*, 179–205. https://doi.org/10.1146/annurev.immunol.14.1.179.

Terstappen, L.W., Huang, S., and Picker, L.J. (1992). Flow cytometric assessment of human T-cell differentiation in thymus and bone marrow. Blood *79*, 666–677. https://doi.org/10.1182/blood.V79.3.666.666.

Themeli, M., Kloss, C.C., Ciriello, G., Fedorov, V.D., Perna, F., Gonen, M., and Sadelain, M. (2013). Generation of tumor-targeted human T lymphocytes from induced pluripotent stem cells for cancer therapy. Nat. Biotechnol. *31*, 928–933. https://doi.org/10.1038/nbt.2678.

Timmermans, F., Velghe, I., Vanwalleghem, L., De Smedt, M., Van Coppernolle, S., Taghon, T., Moore, H.D., Leclercq, G., Langerak, A.W., Kerre, T., et al. (2009). Generation of T cells from human embryonic stem cell-derived hematopoietic zones. J. Immunol. *182*, 6879–6888. https://doi.org/10.4049/jimmunol.0803670.

Tydell, C.C., David-Fung, E.S., Moore, J.E., Rowen, L., Taghon, T., and Rothenberg, E.V. (2007). Molecular dissection of prethymic progenitor entry into the T lymphocyte developmental pathway. J. Immunol. *179*, 421–438. https://doi.org/10.4049/jimmunol.179.1.421.

Van Coppernolle, S., Vanhee, S., Verstichel, G., Snauwaert, S., van der Spek, A., Velghe, I., Sinnesael, M., Heemskerk, M.H., Taghon, T., Leclercq, G., et al. (2012). Notch induces human T-cell receptor gammadelta+ thymocytes to differentiate along a parallel, highly proliferative and bipotent CD4 CD8 double-positive pathway. Leukemia *26*, 127–138. https://doi.org/10.1038/leu.2011.324.

Vizcardo, R., Klemen, N.D., Islam, S.M.R., Gurusamy, D., Tamaoki, N., Yamada, D., Koseki, H., Kidder, B.L., Yu, Z., Jia, L., et al. (2018). Generation of tumor antigen-specific iPSC-derived thymic emigrants using a 3D thymic culture system. Cell Rep. *22*, 3175–3190. https://doi.org/10.1016/j.celrep.2018.02.087.

Vizcardo, R., Masuda, K., Yamada, D., Ikawa, T., Shimizu, K., Fujii, S., Koseki, H., and Kawamoto, H. (2013). Regeneration of human tumor antigen-specific T cells from iPSCs derived from mature CD8(+) T cells. Cell Stem Cell *12*, 31–36. https://doi.org/10.1016/j.stem.2012.12.006.

Voskoboinik, I., Whisstock, J.C., and Trapani, J.A. (2015). Perforin and granzymes: function, dysfunction and human pathology. Nat. Rev. Immunol. *15*, 388–400. https://doi.org/10.1038/nri3839.

Wada, I., Matsushita, H., Noji, S., Mori, K., Yamashita, H., Nomura, S., Shimizu, N., Seto, Y., and Kakimi, K. (2014). Intraperitoneal injection of in vitro expanded Vgamma9Vdelta2 T cells together with zoledronate for the treatment of malignant ascites due to gastric cancer. Cancer Med. *3*, 362–375. https://doi.org/10.1002/cam4.196.

Watanabe, D., Koyanagi-Aoi, M., Taniguchi-Ikeda, M., Yoshida, Y., Azuma, T., and Aoi, T. (2018). The generation of human gammadeltaT cell-derived induced pluripotent stem cells from whole peripheral blood mononuclear cell culture. Stem Cells Transl. Med. *7*, 34–44. https://doi.org/10.1002/sctm.17-0021.

Wrobel, P., Shojaei, H., Schittek, B., Gieseler, F., Wollenberg, B., Kalthoff, H., Kabelitz, D., and Wesch, D. (2007). Lysis of a broad range of epithelial tumour cells by human gamma delta T cells: involvement of NKG2D ligands and T-cell receptor- versus NKG2D-dependent recognition. Scand. J. Immunol. *66*, 320–328. https://doi.org/10.1111/j.1365-3083.2007.01963.x.

Wu, D., Wu, P., Qiu, F., Wei, Q., and Huang, J. (2017). Human gammadeltaT-cell subsets and their involvement in tumor immunity. Cell. Mol. Immunol. *14*, 245–253. https://doi.org/10.1038/cmi.2016.55.

Xu, M., Mizoguchi, I., Morishima, N., Chiba, Y., Mizuguchi, J., and Yoshimoto, T. (2010). Regulation of antitumor immune responses by the IL-12 family cytokines, IL-12, IL-23, and IL-27. Clin. Dev. Immunol. *2010*. https://doi.org/10.1155/2010/832454.

Yang, Y., Liu, F., Liu, W., Ma, M., Gao, J., Lu, Y., Huang, L.H., Li, X., Shi, Y., Wang, X., et al. (2020). Analysis of single-cell RNAseq identifies transitional states of T cells associated with hepatocellular carcinoma. Clin. Transl. Med. *10*, e133. https://doi.org/10.1002/ctm2.133.

Yao, S., Buzo, B.F., Pham, D., Jiang, L., Taparowsky, E.J., Kaplan, M.H., and Sun, J. (2013). Interferon regulatory factor 4 sustains CD8(+) T cell expansion and effector differentiation. Immunity *39*, 833–845. https://doi.org/10.1016/j.immuni.2013.10.007.

Zeng, J., Tang, S.Y., and Wang, S. (2019). Derivation of mimetic gammadelta T cells endowed with cancer recognition receptors from reprogrammed gammadelta T cell. PLoS One *14*, e0216815. https://doi.org/10.1371/journal.pone.0216815.

Stem Cell Reports, Volume *18*

Supplemental Information

Re-generation of cytotoxic $\gamma\delta$T cells with distinctive signatures from human $\gamma\delta$T-derived iPSCs

Nobuyuki Murai, Michiyo Koyanagi-Aoi, Hiroto Terashi, and Takashi Aoi

Figure.S1

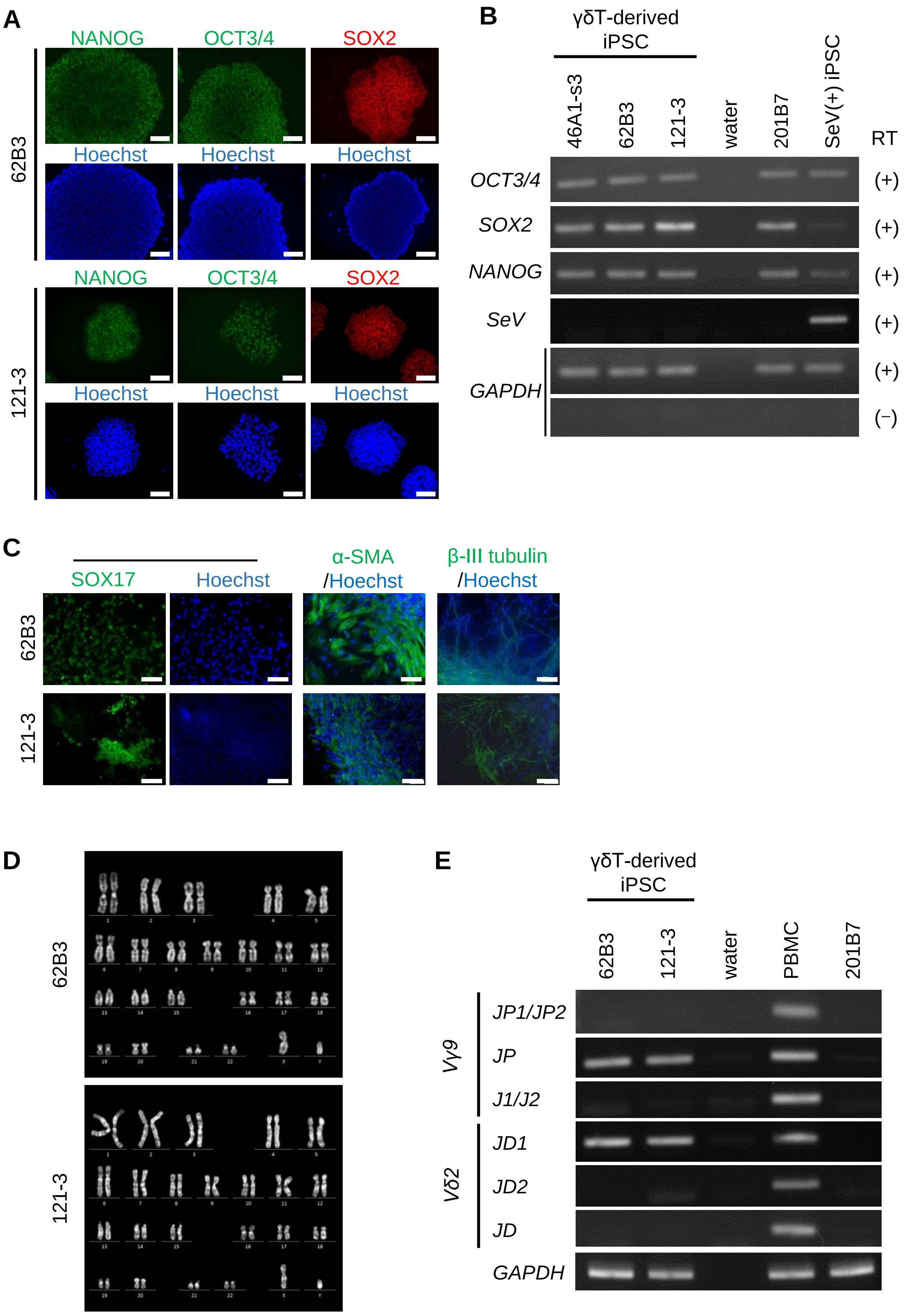

A
NANOG
OCT3/4
SOX2
Hoechst
62B3
121-3
B
γδT-derived iPSC
46A1-s3
62B3
121-3
water
201B7
SeV(+) iPSC
RT
OCT3/4
SOX2
NANOG
SeV
GAPDH
(+)
(−)
C
SOX17
Hoechst
α-SMA /Hoechst
β-III tubulin /Hoechst
62B3
121-3
D
62B3
121-3
E
γδT-derived iPSC
62B3
121-3
water
PBMC
201B7
Vγ9
JP1/JP2
JP
J1/J2
Vδ2
JD1
JD2
JD
GAPDH

Figure.S2

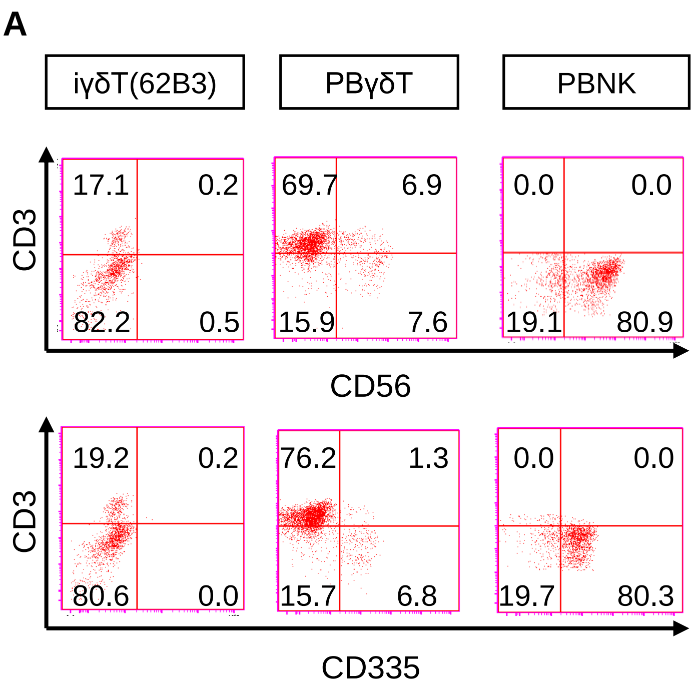
A
iγδT(62B3)
PBγδT
PBNK
CD3
17.1
0.2
82.2
0.5
69.7
6.9
15.9
7.6
0.0
0.0
19.1
80.9
CD56
CD3
19.2
0.2
80.6
0.0
76.2
1.3
15.7
6.8
0.0
0.0
19.7
80.3
CD335

Figure.S3

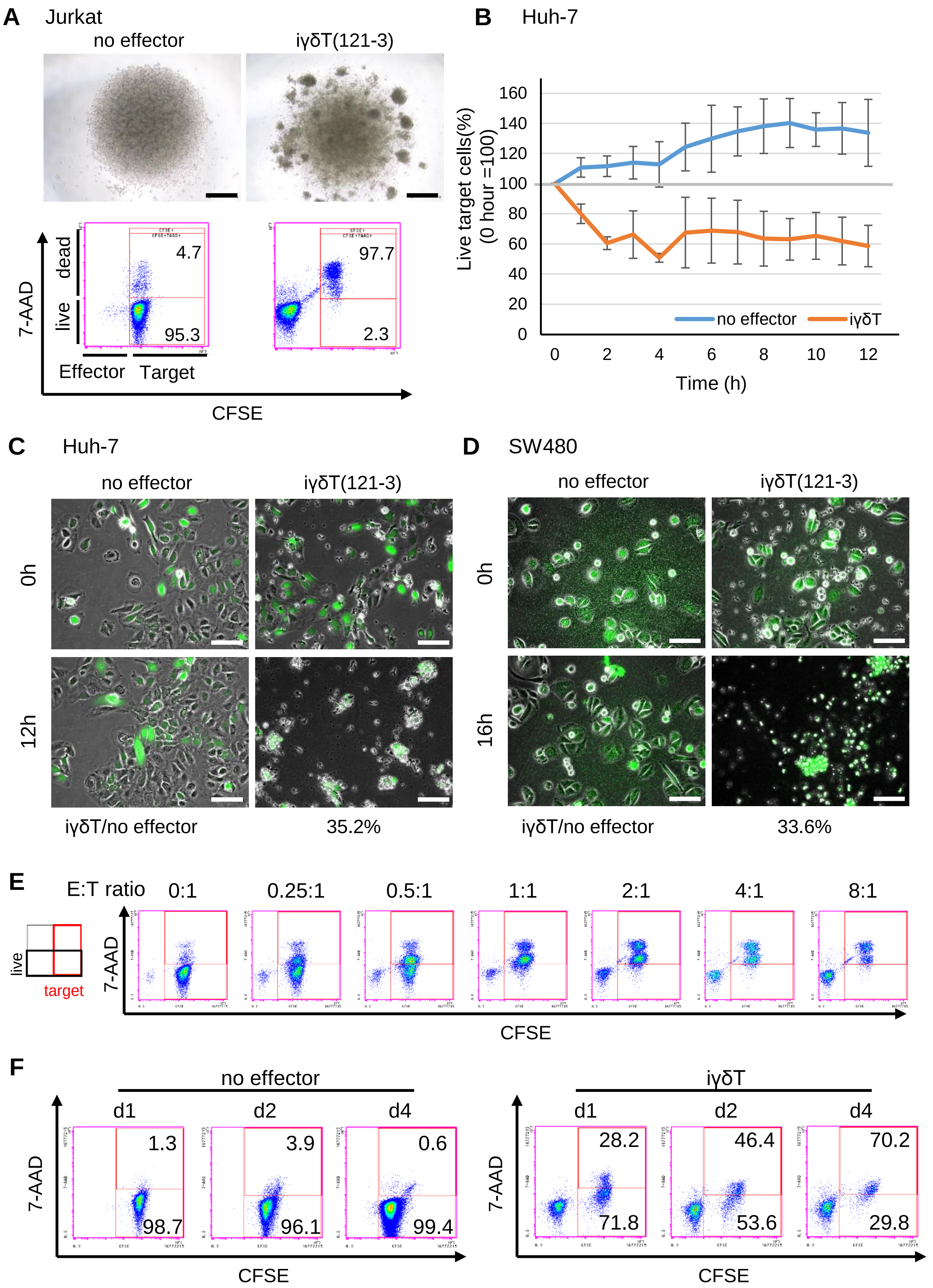

A
Jurkat
no effector
iγδT(121-3)
7-AAD
dead
live
4.7
95.3
97.7
2.3
Effector
Target
CFSE
B
Huh-7
Live target cells(%)
(0 hour =100)
160
140
120
100
80
60
40
20
0
0
2
4
6
8
10
12
Time (h)
no effector
iγδT
C
Huh-7
no effector
iγδT(121-3)
0h
12h
iγδT/no effector
35.2%
D
SW480
no effector
iγδT(121-3)
0h
16h
iγδT/no effector
33.6%
E
E:T ratio
0:1
0.25:1
0.5:1
1:1
2:1
4:1
8:1
live
target
7-AAD
CFSE
F
no effector
iγδT
d1
d2
d4
1.3
98.7
3.9
96.1
0.6
99.4
28.2
71.8
46.4
53.6
70.2
29.8
7-AAD
CFSE

Figure.S4

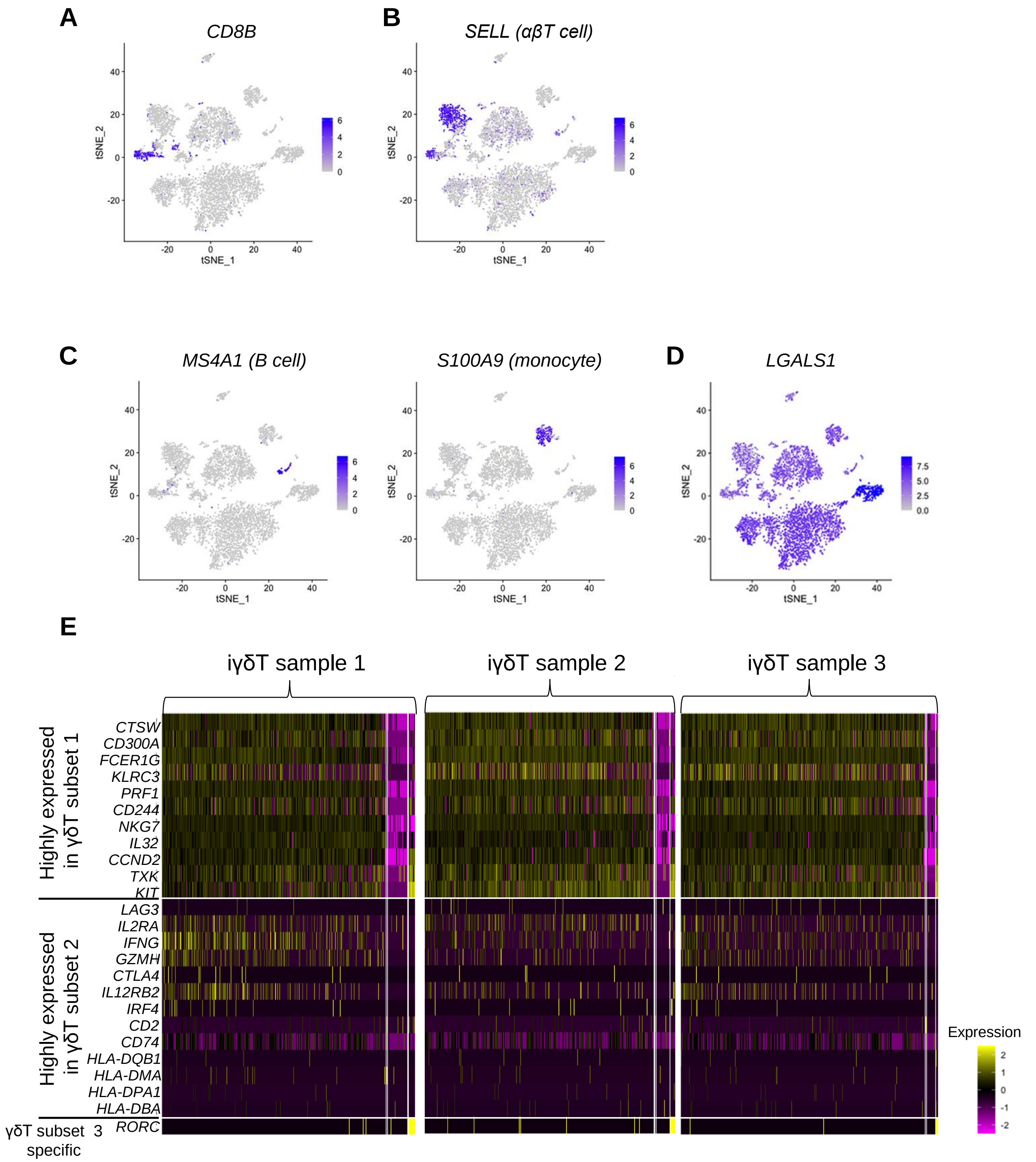
A
CD8B
B
SELL (αβT cell)
C
MS4A1 (B cell)
S100A9 (monocyte)
D
LGALS1
tSNE_1
tSNE_2
E
iγδT sample 1
iγδT sample 2
iγδT sample 3
Highly expressed in γδT subset 1
CTSW
CD300A
FCER1G
KLRC3
PRF1
CD244
NKG7
IL32
CCND2
TXK
KIT
Highly expressed in γδT subset 2
LAG3
IL2RA
IFNG
GZMH
CTLA4
IL12RB2
IRF4
CD2
CD74
HLA-DQB1
HLA-DMA
HLA-DPA1
HLA-DBA
γδT subset 3 specific
RORC
Expression

SUPPLEMENTARY FIGURE LEGENDS

Figure S1. Validation of γδT-iPSCs

A) Immunofluorescent staining of pluripotent cell markers NANOG, OCT3/4 and SOX2 in 62B3 (upper panels) and 121-3 (lower panels). Nuclei were stained with Hoechst 33342. Scale bars indicate 100 µm.

B) RT-PCR to detect pluripotent cell markers. The absence of Sendai virus (SeV) in γδT-derived iPSCs (46A1-s3, 62B3 and 121-3) was also confirmed. The conventional clone 201B7, which was established by retrovirus vectors, was used as a negative control. RT: reverse transcriptase.

C) *In vitro* differentiation via embryoid body formation of 62B3 (upper panels) and 121-3 (lower panels). Immunofluorescent staining for SOX17 (endoderm marker), α-SMA (mesoderm marker) and β-III-tubulin (ectoderm marker) in γδT-iPSCs-derived differentiated cells. Scale bars indicate 50 µm.

D) Karyotyping of γδT-iPSCs. Images of the Q-band analysis are shown.

E) Genomic PCR for TCR gene rearrangement in γδT- iPSCs. PBMCs were used as a positive control. The conventional iPSC clone 201B7, which was established from fibroblasts, was used as a negative control.

Figure S2. Flow cytometry for NK cell markers

A) Cell surface markers, including CD3/ CD56 (upper panels) and CD3/ CD335 (NKp46) (lower panels) were analyzed by flow cytometry. Each cell was stimulated (iγδT at day 48, PBγδT and PBNK at day 18) and not sorted.

Figure S3. Verification of Cytotoxicity in iγδTs

A) Phase contrast images and dot plots for CFSE and 7-AAD staining in CFSE-stained Jurkat cells (target) co-cultured with or without 121-3-derived iγδTs (effector) for one day (E:T ratio = 2: 1). Scale bars indicate 500μm (upper panels). Numbers in scattergrams indicate the percentages of live or dead cells in CFSE-stained target cells (lower panels).

B) The time course analysis of the relative proportion of live GFP-Huh-7 cells (=GFP-positive area) during co-culture with or without iγδTs. A series of image frames were acquired by fluorescence microscopy at 1 h/frame over a 12 h period. The area of GFP-positive cells at 0 h in each condition was set to 100 %. Changes in relative proportion of live target cells were shown (n=3 independent experiments, mean ±SD). See also Figure 3C.

C) Representative continuous images at 0 and 12 h displaying GFP-labeled Huh-7 cells co-cultured with or without 121-3 derived iγδTs are shown. The proportion of GFP-positive area at 12 h in target cells co-cultured with iγδT was 35.2% of that with no effector, which was set to 100%. Scale bars indicate 100 μm.

D) Representative continuous images at 0 and 16 h displaying CFSE-labeled SW480 cells co-cultured with or without 121-3 derived iγδTs are shown. The proportion of CFSE-positive area at 16 h in target cells co-cultured with iγδTs was 33.6% of that with no effector, which was set to 100%. Scale bars indicate 100 μm.

E) Dot plots showing the cytotoxicity of 62B3-derived iγδTs against Jurkat cells, which was analyzed using a CFSE/7-AAD assay with the indicated E:T ratios after one day of co-culture. See also Figure 4.

F) Dot plots showing the results of the time course analysis of the proportions of live Jurkat cells co-cultured with or without 62B3-derived iγδTs for 4 days at an E:T ratio of 0.5: 1. Numbers in scattergrams indicate the percentage of live or dead cells in CFSE-stained target cells. See also Figure 4B.

Figure S4. Characterization of each cluster and reproducibility of γδT cell induction from iPSCs

A)-D) Feature plots show the expression of CD8B (A), SELL (B), MS4A1 and S100A9 (C) and LGALS1 (D) to define clusters. Blue indicates a high expression level; light grey indicates no expression.

E) Heatmap of genes that were highly expressed in γδT subset 1 (n=11), γδT subset 2 (n=13) and γδT subset 3 (n=1) in iγδTs from three independent experiments. Three iγδT samples were resultant cells stimulated with HMBPP for 6, 11, and 12 days respectively.

SUPPLEMENTAL TABLES

Table S1. Repertoire analysis of PBγδTs.

Rearrangement of the TCRγ (TRG), TCRδ (TRD) gene locus and amino acid sequence of CDR3, related to Figure 2C.

ranking	*TRGV*	*TRGJ*	CDRJ	%reads	ranking	*TRDV*	*TRDJ*	CDRJ	%reads
1	*TRGV9*	*TRGJP*	CALWEVQELGKKIKVF	13.04841	1	*TRDV2*	*TRDJ3*	CACDSGEAGWDTRQMFF	19.46068
2	*TRGV9*	*TRGJP*	CALWEEELGKKIKVF	5.168883	2	*TRDV2*	*TRDJ1*	CACDTLLKQGDLITDKLIF	8.034431
3	*TRGV9*	*TRGJP*	CALWEVLELGKKIKVF	4.480577	3	*TRDV1*	*TRDJ1*	CALGPRYFRNWGIRPNADKLIF	6.500837
4	*TRGV10*	*TRGJP1*	CAAWDRPRRWFKIF	3.634133	4	*TRDV2*	*TRDJ1*	CACDTVDGGYAHTDKLIF	4.056395
5	*TRGV9*	*TRGJP*	CALWEVRELGKKIKVF	3.221555	5	*TRDV2*	*TRDJ1*	CACDTVGDSNTDKLIF	3.667546
6	*TRGV9*	*TRGJP*	CALWEVKAPQELGKKIKVF	3.204322	6	*TRDV2*	*TRDJ1*	CACAALPTMGMGYTDKLIF	3.502581
7	*TRGV8*	*TRGJP2*	CATWDRGSDWIKTF	3.186076	7	*TRDV2*	*TRDJ1*	CACDTVLARQYTDKLIF	3.142012
8	*TRGV9*	*TRGJP*	CALWEEFQELGKKIKVF	2.777553	8	*TRDV2*	*TRDJ3*	CACDHWGSSSWDTRQMFF	2.853322
9	*TRGV9*	*TRGJP*	CALWEAQELGKKIKVF	2.753224	9	*TRDV2*	*TRDJ3*	CACDTVSSWDTRQMFF	2.023189
10	*TRGV9*	*TRGJP*	CALWEVEELGKKIKVF	2.4704	10	*TRDV2*	*TRDJ3*	CACDTAITGGSSSWDTRQMFF	2.004925
11	*TRGV9*	*TRGJP*	CALWEALQELGKKIKVF	2.36092	11	*TRDV2*	*TRDJ1*	CACEALAYTDKLIF	1.787524
12	*TRGV9*	*TRGJP*	CALWEVQRAQELGKKIKVF	2.282864	12	*TRDV2*	*TRDJ1*	CACDTVGGGYASDKLIF	1.738034
13	*TRGV9*	*TRGJP*	CALWEMQELGKKIKVF	2.237248	13	*TRDV2*	*TRDJ1*	CACDTVGGPYTDKLIF	1.555982
14	*TRGV9*	*TRGJP*	CALWEVKELGKKIKVF	1.89056	14	*TRDV2*	*TRDJ1*	CACDTLGDRRTDKLIF	1.554215
15	*TRGV10*	*TRGJP1*	CAAWDGPTGWFKIF	1.834807	15	*TRDV2*	*TRDJ1*	CACDTLLGDKVDKLIF	1.535362
16	*TRGV9*	*TRGJP*	CALWESQELGKKIKVF	1.732422	16	*TRDV2*	*TRDJ1*	CACDTIPGGSLGTDKLIF	1.517687
17	*TRGV9*	*TRGJP*	CALWEDPELGKKIKVF	1.592531	17	*TRDV2*	*TRDJ1*	CACDIMGDTPFADKLIF	1.477034
18	*TRGV5*	*TRGJ2*	CATWDRLDYYKKLF	1.550969	18	*TRDV2*	*TRDJ1*	CACDGLGDIPYTDKLIF	1.386303

19	*TRGV8*	*TRGJ2*	CATWGRAVYYKKLF	1.512448	19	*TRDV2*	*TRDJ3*	CACDTSTGGPFSWDTRQMFF	1.384536
20	*TRGV9*	*TRGJP*	CALWEVQERELGKKIKVF	1.37053	20	*TRDV2*	*TRDJ1*	CACDTVLTGGYVDDKLIF	1.293215
21	*TRGV9*	*TRGJP*	CALWEVWELGKKIKVF	1.295515	21	*TRDV2*	*TRDJ1*	CACDTVGGHGGTDKLIF	1.274951
22	*TRGV9*	*TRGJP*	CALWEVSEELGKKIKVF	1.161706	22	*TRDV2*	*TRDJ1*	CACDTVGIRGPDKLIF	1.213678
23	*TRGV8*	*TRGJ2*	CATWDKNYYKKLF	1.134336	23	*TRDV2*	*TRDJ1*	CACDTLLGDTREGDKLIF	1.201306
24	*TRGV8*	*TRGJ2*	CATWGWTRNYYKKLF	1.117103	24	*TRDV2*	*TRDJ1*	CAVLPVGHITDKLIF	1.200127
25	*TRGV9*	*TRGJ2*	CALWEGSNYYKKLF	1.098857	25	*TRDV2*	*TRDJ1*	CACETLDGGSQYTDKLIF	1.199538
26	*TRGV9*	*TRGJ2*	CALWEVQLAGRYKKLF	0.937677	26	*TRDV2*	*TRDJ1*	CACDPVLGDTTYTDKLIF	1.166545
27	*TRGV9*	*TRGJP*	CALWESQQELGKKIKVF	0.90017	27	*TRDV2*	*TRDJ1*	CACDSIGGGYASQLIF	1.140622
28	*TRGV9*	*TRGJP*	CALWEVVIELGKKIKVF	0.858608	28	*TRDV2*	*TRDJ1*	CACDSLRPGGAHTDKLIF	1.117055
29	*TRGV9*	*TRGJP*	CALWEEQELGKKIKVF	0.840362	29	*TRDV2*	*TRDJ1*	CACDTAYAGSTDKLIF	1.100559
30	*TRGV9*	*TRGJP*	CALWEVGELGKKIKVF	0.835293	30	*TRDV2*	*TRDJ1*	CACDSVTGGYTPDKLIF	1.078759
31	*TRGV9*	*TRGJP*	CALWEAKQELGKKIKVF	0.795759	31	*TRDV2*	*TRDJ1*	CACDTLGDQPRHTDKLIF	1.016308
32	*TRGV3*	*TRGJ2*	CATWDISYYKKLF	0.765347	32	*TRDV2*	*TRDJ1*	CACDTVKGLGENTDKLIF	0.721137
33	*TRGV9*	*TRGJP*	CALWELELGKKIKVF	0.746087	33	*TRDV2*	*TRDJ1*	CACDTIGLMLGERRDTDKLIF	0.716424
34	*TRGV9*	*TRGJ1*	CALWEVSPHKKLF	0.701484	34	*TRDV2*	*TRDJ1*	CACDSLGGPYTDKLIF	0.688144
35	*TRGV9*	*TRGJP*	CALWEESQELGKKIKVF	0.680196	35	*TRDV2*	*TRDJ1*	CACDPWGPHTDKLIF	0.643956
36	*TRGV9*	*TRGJP*	CALWIQELGKKIKVF	0.670059	36	*TRDV2*	*TRDJ1*	CACDTLPTRGVLGDTLYTDKLIF	0.592699
37	*TRGV9*	*TRGJP*	CALWEVRSELGKKIKVF	0.644717	37	*TRDV2*	*TRDJ3*	CACDKTGGHLSSWDTRQMFF	0.586807
38	*TRGV9*	*TRGJP*	CALWSQELGKKIKVF	0.59099	38	*TRDV2*	*TRDJ1*	CACRSLGEDTDKLIF	0.56383
39	*TRGV9*	*TRGJP*	CALWEVPELGKKIKVF	0.580853	39	*TRDV2*	*TRDJ3*	CACDTSGGYASSWDTRQMFF	0.524945
40	*TRGV9*	*TRGJP*	CALWEVQIGELGKKIKVF	0.577812	40	*TRDV2*	*TRDJ1*	CACDTFPVLGDKELIF	0.5002
41	*TRGV9*	*TRGJP*	CALWEVLGLQELGKKIKVF	0.53625	41	*TRDV2*	*TRDJ3*	YACDSGEAGWDTRQMFF	0.443641
42	*TRGV9*	*TRGJP*	CALWEHKGEEKLGKKIKVF	0.518003	42	*TRDV2*	*TRDJ1*	CACDTILGDTSDTDKLIF	0.384724
43	*TRGV9*	*TRGJP*	CALWAAELGKKIKVF	0.512935	43	*TRDV2*	*TRDJ1*	CACDDLGDTATDKLIF	0.378243
44	*TRGV9*	*TRGJP*	CALWEAHEELGKKIKVF	0.501784	44	*TRDV2*	*TRDJ3*	CACDTGFVGGSHSWDTRQMFF	0.375298

45	*TRGV9*	*TRGJP*	CALWELQELGKKIKVF	0.497729	45	*TRDV2*	*TRDJ1*	CACDTVKRGPHLTDKLIF	0.273961
46	*TRGV9*	*TRGJP*	CALWEPTRRTQELGKKIKVF	0.495702	46	*TRDV2*	*TRDJ1*	CACDTLDPLGDTDHTDKLIF	0.244503
47	*TRGV9*	*TRGJP*	CALWEVHELGKKIKVF	0.477455	47	*TRDV2*	*TRDJ3*	CACDSEEAGWDTRQMFF	0.076591
48	*TRGV9*	*TRGJP*	CALWEADPQELGKKIKVF	0.471373	48	*TRDV2*	*TRDJ1*	CACGTLLKQGDLITDKLIF	0.074235
49	*TRGV9*	*TRGJP*	CALWEVRLELGKKIKVF	0.464277	49	*TRDV2*	*TRDJ1*	CVCDTLGDRRTDKLIF	0.060684
50	*TRGV9*	*TRGJP*	CALWEVMELGKKIKVF	0.463263	50	*TRDV2*	*TRDJ4*	CARPLIF	0.05656

Table S2. Repertoire analysis of iγδTs.

Rearrangement of the TCRγ (TRG), TCRδ (TRD) gene locus and amino acid sequence of CDR3, related to Figure 2C.

ranking	*TRGV*	*TRGJ*	CDRJ	%reads	ranking	*TRDV*	*TRDJ*	CDRJ	%reads
1	*TRGV9*	*TRGJP*	CALWEVQELGKKIKVF	94.51407	1	*TRDV2*	*TRDJ1*	CACDPLLGDTPRYTDKLIF	90.05091
2	*TRGV9*	*TRGJP*	CALWEVQELDKKIKVF	0.254869	2	*TRDV2*	*TRDJ1*	CACEPLLGDTPRYTDKLIF	0.330323
3	*TRGV9*	*TRGJP*	CALWEVQELSKKIKVF	0.242366	3	*TRDV2*	*TRDJ1*	CACDPLLGDMPRYTDKLIF	0.264663
4	*TRGV9*	*TRGJP*	CALWGVQELGKKIKVF	0.166386	4	*TRDV2*	*TRDJ1*	CACDPLLGDTPGYTDKLIF	0.259612
5	*TRGV9*	*TRGJP*	GALWEVQELGKKIKVF	0.142342	5	*TRDV2*	*TRDJ1*	CACDPLLGDTTRYTDKLIF	0.236378
6	*TRGV9*	*TRGJP*	CALWEVQELGKKSKVF	0.142342	6	*TRDV2*	*TRDJ1*	CACDPLLGGTPRYTDKLIF	0.210114
7	*TRGV9*	*TRGJP*	CALWELQELGKKIKVF	0.123107	7	*TRDV2*	*TRDJ1*	CACDQLLGDTPRYTDKLIF	0.204053
8	*TRGV9*	*TRGJP*	CALWEVKELGKKIKVF	0.118298	8	*TRDV2*	*TRDJ1*	CACDTLLGDTPRYTDKLIF	0.192941
9	*TRGV9*	*TRGJP*	CVLWEVQELGKKIKVF	0.114451	9	*TRDV2*	*TRDJ1*	CDCDPLLGDTPRYTDKLIF	0.1889
10	*TRGV9*	*TRGJP*	CAVWEVQELGKKIKVF	0.089445	10	*TRDV2*	*TRDJ1*	CARDPLLGDTPRYTDKLIF	0.182839
11	*TRGV9*	*TRGJP*	CALWEVRELGKKIKVF	0.083674	11	*TRDV2*	*TRDJ1*	CVCDPLLGDTPRYTDKLIF	0.164656
12	*TRGV9*	*TRGJP*	RALWEVQELGKKIKVF	0.082712	12	*TRDV2*	*TRDJ1*	CACDPILGDTPRYTDKLIF	0.162636
13	*TRGV9*	*TRGJP*	CALWEVQELGKKIKVV	0.076942	13	*TRDV2*	*TRDJ1*	CACDPLLGDKPRYTDKLIF	0.153545

14	*TRGV9*	*TRGJP*	YALWEVQELGKKI KVF	0.073094
15	*TRGV9*	*TRGJP*	SALWEVQELGKKI KVF	0.073094
16	*TRGV9*	*TRGJP*	CASWEVQELGKKI KVF	0.073094
17	*TRGV9*	*TRGJP*	CALWEVQELGKKI KVC	0.069247
18	*TRGV9*	*TRGJP*	CALWEVQELGKRI KVF	0.068286
19	*TRGV9*	*TRGJP*	CALWEVQELGKKI KGF	0.063477
20	*TRGV9*	*TRGJP*	CALWEVQELGKKI EVF	0.063477
21	*TRGV9*	*TRGJP*	WALWEVQELGKK IKVF	0.062515
22	*TRGV9*	*TRGJP*	CAWWEVQELGKK IKVF	0.061553
23	*TRGV9*	*TRGJP*	CALWEVQELGKKI KVL	0.061553
24	*TRGV9*	*TRGJP*	CDLWEVQELGKKI KVF	0.05963
25	*TRGV9*	*TRGJP*	CALWEVQELGKEI KVF	0.056744
26	*TRGV9*	*TRGJP*	CTLWEVQELGKKI KVF	0.051936
27	*TRGV9*	*TRGJP*	CALWEVQELGKKI RVF	0.051936
28	*TRGV9*	*TRGJP*	CALWEVQGLGKKI KVF	0.050974
29	*TRGV9*	*TRGJP*	CALWEVQELGKK NKVF	0.050012
30	*TRGV9*	*TRGJP*	CAMWEVQELGKK IKVF	0.04905

14	*TRDV2*	*TRDJ1*	CACDPLLGDTPRYTD KRIF	0.151524
15	*TRDV2*	*TRDJ1*	CACDPRLGDTPRYTD KLIF	0.148494
16	*TRDV2*	*TRDJ1*	WACDPLLGDTPRYTD KLIF	0.137382
17	*TRDV2*	*TRDJ1*	CACGPLLGDTPRYTD KLIF	0.133341
18	*TRDV2*	*TRDJ1*	CACDPLMGDTPRYTD KLIF	0.117179
19	*TRDV2*	*TRDJ1*	CACDPLLGDTPKYTD KLIF	0.105057
20	*TRDV2*	*TRDJ1*	CACDPLLGDTQRYTD KLIF	0.103037
21	*TRDV2*	*TRDJ1*	CACDPLLRDTPRYTD KLIF	0.101016
22	*TRDV2*	*TRDJ1*	CACDPLLGDTPRYTN KLIF	0.100006
23	*TRDV2*	*TRDJ1*	CTCDPLLGDTPRYTD KLIF	0.096976
24	*TRDV2*	*TRDJ1*	CACDPLRGDTPRYTD KLIF	0.088894
25	*TRDV2*	*TRDJ1*	CGCDPLLGDTPRYTD KLIF	0.086874
26	*TRDV2*	*TRDJ1*	CAYDPLLGDTPRYTD KLIF	0.086874
27	*TRDV2*	*TRDJ1*	CACDPLLGDTPRYTD KLIL	0.078793
28	*TRDV2*	*TRDJ1*	CACNPLLGDTPRYTD KLIF	0.072732
29	*TRDV2*	*TRDJ1*	CACDPLLGNTPRYTD KLIF	0.071722
30	*TRDV2*	*TRDJ1*	CACDPLLEDTPRYTD KLIF	0.070711

31	*TRGV9*	*TRGJP*	CALWEVQELGKKIKVY	0.045203
32	*TRGV9*	*TRGJP*	CALWEVQELGKKIKLF	0.041356
33	*TRGV9*	*TRGJP*	CALREVQELGKKIKVF	0.041356
34	*TRGV9*	*TRGJP*	CALWEVQELGKKINVF	0.040394
35	*TRGV9*	*TRGJP*	CALWEVQELCKKIKVF	0.039433
36	*TRGV9*	*TRGJP*	CALWEMQELGKKIKVF	0.039433
37	*TRGV9*	*TRGJP*	CALWEVQKLGKKIKVF	0.037509
38	*TRGV9*	*TRGJP*	CALWKVQELGKKIKVF	0.036547
39	*TRGV9*	*TRGJP*	CALWEVQEFGKKIKVF	0.035585
40	*TRGV9*	*TRGJP*	CALWEVQELGKKIKIF	0.034624
41	*TRGV9*	*TRGJP*	CGLWEVQELGKKIKVF	0.033662
42	*TRGV9*	*TRGJP*	CALWEVEELGKKIKVF	0.0327
43	*TRGV9*	*TRGJP*	CALWEVQEWGKKIKVF	0.031738
44	*TRGV9*	*TRGJP*	CALLEVQELGKKIKVF	0.031738
45	*TRGV9*	*TRGJP*	CALCEVQELGKKIKVF	0.031738
46	*TRGV9*	*TRGJP*	CALWEGQELGKKIKVF	0.030777
47	*TRGV9*	*TRGJP*	CALWEVQELRKKIKVF	0.028853

31	*TRDV2*	*TRDJ1*	YACDPLLGDTPRYTDKLIF	0.068691
32	*TRDV2*	*TRDJ1*	CASDPLLGDTPRYTDKLIF	0.067681
33	*TRDV2*	*TRDJ1*	CACDPLLGDTPRYNDKLIF	0.066671
34	*TRDV2*	*TRDJ1*	RACDPLLGDTPRYTDKLIF	0.06465
35	*TRDV2*	*TRDJ1*	CACDPLLGDTPRYTGKLIF	0.057579
36	*TRDV2*	*TRDJ1*	CACDPLLGDTPRYTDKLSF	0.057579
37	*TRDV2*	*TRDJ1*	CSCDPLLGDTPRYTDKLIF	0.055559
38	*TRDV2*	*TRDJ1*	CACDPLLGDTPRYTEKLIF	0.054549
39	*TRDV2*	*TRDJ1*	CACDPLLGDTPRDTDKLIF	0.054549
40	*TRDV2*	*TRDJ1*	CACDPLLGDAPRYTDKLIF	0.054549
41	*TRDV2*	*TRDJ1*	CAWDPLLGDTPRYTDKLIF	0.052528
42	*TRDV2*	*TRDJ1*	CACDPLLGDTPRYIDKLIF	0.052528
43	*TRDV2*	*TRDJ1*	CACDPLLGDTLRYTDKLIF	0.052528
44	*TRDV2*	*TRDJ1*	CAGDPLLGDTPRYTDKLIF	0.050508
45	*TRDV2*	*TRDJ1*	CACDPLLGDTPRYTDNLIF	0.050508
46	*TRDV2*	*TRDJ1*	CACDPLLGDTPRYTDKPIF	0.049498
47	*TRDV2*	*TRDJ1*	CACDPLLGDTPRYTDKLVF	0.049498

48	*TRGV9*	*TRGJP*	CALWEAQELGKKIKVF	0.028853
49	*TRGV9*	*TRGJP*	CALWEVQELGKKIQVF	0.02693
50	*TRGV9*	*TRGJP*	CSLWEVQELGKKIKVF	0.025968

48	*TRDV2*	*TRDJ1*	CACDPLLGDTPRYTDKHIF	0.045457
49	*TRDV2*	*TRDJ1*	CACDPLQGDTPRYTDKLIF	0.044447
50	*TRDV2*	*TRDJ1*	CACDPLLGDTSRYTDKLIF	0.043437

Table S3. Primers for RT-PCR to detect undifferentiated marker genes, related to Figure S1B

GAPDH	forward	AGCCACATCGCTCAGACAC
	reverse	GCCCAATACGACCAAATCC
SeV	forward	GGATCACTAGGTGATATCGAGC
	reverse	ACCAGACAAGAGTTTAAGAGATATGTATC
OCT3/4	forward	GACAGGGGGAGGGGAGGAGCTAGG
	reverse	CTTCCCTCCAACCAGTTGCCCCAAAC
SOX2	forward	GGGAAATGGGAGGGGTGCAAAAGAGG
	reverse	TTGCGTGAGTGTGGATGGGATTGGTG
NANOG	forward	TGAACCTCAGCTACAAACAG
	reverse	TGGTGGTAGGAAGAGTAAAG

Table S4. Primers for genomic PCR of TCR gene rearrangement, related to Figure S1E.

TCRG	forward	*Vγ9*	CGGCACTGTCAGAAAGGAATC
	reverse	*TCRG-JP1.JP2*	GAAGTTACTATGAGCTTAGTCCCTT
		TCRG-JP	AAGCTTTGTTCCGGGACCAAATAC
		TCRG-J1.J2	TACCTGTGACAACAAGTGTTGTTC
TCRD	forward	*Vδ2*	ATACCGAGAAAAGGACATCTATG
	reverse	*JD1*	GTTCCACAGTCACACGGGTTC
		JD2	GTTCCACGATGAGTTGTGTTC
		JD3	CTCACGGGGCTCCACGAAGAG

Table S5. Antibodies used in flowcytometry and immunofluorescent staining

Antibodies		
anti-human CD34-APC	Biolegend	Clone:561, Cat:343607
anti-human CD43-PE	eBiosciences	Clone:84-3C1, Cat:12-0439
anti-human CD45RA-FITC	eBiosciences	Clone:HI100, Cat:11-0458
anti-human CD27-PE	eBiosciences	Clone:O323, Cat:12-0279
anti-human CD25-PE	eBiosciences	Clone:BC96, Cat:12-0259
anti-human CD7-FITC	eBiosciences	Clone:4H9, Cat:17-0078
anti-human CD5-PE	eBiosciences	Clone:VCHT2, Cat:12-0059
anti-human CD3-PC7	eBiosciences	Clone:UCHT1, Cat:25-0038
anti-human αβTCR-FITC	eBiosciences	Clone:WT31, Cat:11-9955
anti-human γδTCR-PE	eBiosciences	Clone:B11, Cat:12-9959
anti-human TCRγ9-FITC	Beckman Coulter	Clone:IMMU360, Cat:IM1463
anti-human GranzymeB-PE	eBiosciences	Clone:GB11, Cat:12-8899
anti-human Perforin-FITC	Biolegend	Clone:dG9, Cat:308103
anti-human Interferon γ-PC7	Biolegend	Clone:4S.B3, Cat:502527
anti-human CD56-PE	eBiosciences	Clone:TULY56, Cat:12-0566-41
anti-human CD335-PE	Biolegend	Clone:9E2, Cat:331907
anti Oct3/4	BD	Clone:40-Oct3, Cat:611202
anti Nanog	R&D Systems	Polyclonal, Cat:AF1997
anti SOX2	abcam	Polyclonal, Cat:ab97959
anti SOX17	R&D Systems	Polyclonal, Cat:AF1924
anti α-SMA	DAKO	Clone:1A4. Cat:M0851
anti βIII-tubulin	Millipore	Clone:TU20, Cat:MAB1637
anti TCRγδ	eBiosciences	Clone:B1.1, Cat:14-9959-82
anti NKG2D	eBiosciences	Clone:1D11, Cat:14-5878-82

SUPPLEMENTAL EXPERIMENTAL PROCEDURE

Establishment of human induced pluripotent stem cell lines from human γδT cell

The generation of γδT-derived hiPSC clone 121-3 was performed as previously described (Watanabe et al., 2018) with slight modifications. Peripheral blood mononuclear cells (PBMCs) were collected from healthy volunteers after obtaining their informed consent. First, PBMCs were separated from whole blood using a BD Vacutainer blood collection tube and stimulated with 5 μM Zoledronic acid in RPMI 1640 medium (Nacalai Tesque, #30264-56) supplemented with 10% fetal bovine serum (Sigma-Aldrich, F7524), 100 IU/mL IL-2 (Imunace, Shionogi Pharmaceuticals), 10 μM 2-mercaptoethanol (Gibco, 21985-023), 50 U/mL penicillin and 50 μg/mL streptomycin (Life Technologies, 15140-122). Stimulated PBMCs were suspended in 100 μl medium with Cytotune-iPS 2.0 (ID pharma, 69000-41) at an MOI of 2 and cultivated in a 96-well plate. Twenty-four hours later, the cells were plated on a 6-well plate pre-coated with i-Matrix-511 silk (Nippi, 892 024). After transduction, Stemfit medium (Ajinomoto, AK02N) was changed every two days. From day 21, we started to pick up and expand colonies.

The experiment was approved by the institutional review board of Kobe University Graduate School of Medicine.

iPSC culture

We cultured iPSC lines according to a previously described method (Miyazaki et al., 2017). Passaging was performed once a week. After aspirating supernatant and PBS(-) washing, cells were dissociated with 0.5× TrypLE select (1× TrypLE select [Thermo Fisher, A1285901] diluted 1:1 with 0.5 mM EDTA/PBS[-]) and seeded with StemFit medium with 10 μM Y-27632 (WAKO, 034-24024) and iMatrix-511 silk (0.167 μg/cm^2) in un-coated usage. After 24 hours, the medium was refreshed with StemFit medium without Y-27632. The cells were maintained at 37°C under 5% CO_2.

To examine pluripotency, the conventional iPSC clone 201B7, which was established from fibroblasts, was used as a positive control

Feeder cell culture

The feeder cell line: OP9/N-DLL1 was purchased from Riken Cell Bank (RCB2927). OP9/N-DLL1 cells were maintained in αMEM (Thermo Fisher, 11900-016) supplemented with 20% fetal bovine serum, 50 U/ml penicillin, 50 μg/ml streptomycin and 2 mM L-glutamine (Life technologies, 25036-081). Media was changed every other day. Passaging was done once a week. After PBS(-) washing, cells were dissociated with 0.25% Trypsin (Gibco, 15090046) and incubated at 37°C for 10 minutes. Cells were collected by pipetting and filtered using a 35 μm cell strainer. After centrifugation at 800 rpm for 4 minutes, cells were suspended in fully refreshed medium and seeded at density of 5.0×10^4 cells/well (12-well plate)

for HPC co-culture and 2.0×10^5 cells/60 mm dish for maintaining culture.

Culture of cancer cells

The Jurkat and Huh-7 cancer cell lines were obtained from Riken Bio Resource Center (Tsukuba, Ibaraki, Japan). The SW480 cancer cell line was obtained from American Type Culture Collection ([ATCC] Manassas, VA). Jurkat cells (Riken BRC, RCB0806) were cultured in RPMI 1640 containing 10% FBS and were passaged every 3 days. The Huh-7 (Riken BRC, RCB1366) and SW480 (ATCC, CCL-228) cell lines were cultured in DMEM (Nacalai tesque, 08458-45) containing 10% FBS. They were passaged every 3–5 days.

RT-PCR

To test the expression of pluripotent genes in γδT cell-derived iPSC clones, total RNA was extracted using TRIzol reagent (Life Technologies, 15596026) and treated with a Turbo DNA-free kit (Life Technologies, AM1907) to remove genomic DNA contamination. Total RNA was reverse transcribed to cDNA using a PrimeScript II 1st Strand Synthesis Kit (Takara, 2690A) with oligo-dT primers according to the manufacturer's instructions. The cDNA was subjected to PCR with a Takara Ex Taq PCR kit (Takara, RR001A). As positive control, 46A1s3, which was established in our previous report, was used (Watanabe et al., 2018). The primer sequences of the RT-PCR are listed in Table S3.

Analysis of TCRG and TCRD gene rearrangement

To analyze the rearrangement of the TCRG and TCRD gene regions, genomic DNA was extracted using PCI solution and subjected to PCR with a Takara Ex Taq PCR kit. The primer sequences are listed in Table S4.

***In vitro* spontaneous differentiation via embryoid body formation.**

For embryoid body (EB) formation, undifferentiated iPSCs were dissociated into single cells, re-suspended in Primate ES medium (Reprocell, RCHEMD001) containing 20 µM Y-27632, and seeded onto low-cell-adhesion 96-well spindle-bottom plates at a density of 1.0×10^4 cells per well. After 7 days of culture, the EBs were transferred to gelatin-coated 24-well plates and cultured in the same medium for another 7 days. The differentiated cells were immunostained with antibodies listed in table S5.

Immunofluorescent staining

Cells were fixed on a culturing plate with 4% paraformaldehyde for 15–30 minutes at room temperature. After PBS(-) washing, cells were permeabilized and blocked with 1% BSA/PBS(-) containing 0.3% TritonX-100 and 5% donkey serum for 1 hour at room temperature. Then cells were incubated with

primary antibodies overnight at 4°C. The cells were rewashed, then incubated with secondary antibody for 1 hour at room temperature. Fluorescence images were obtained using a BZ-X700 microscope (Keyence).

Karyotype analyses

The Q-band karyotype analysis for γδT-iPSC was performed at Chromocenter Inc. (Yonago, Japan).

Repertoire analysis

CD3+γδTCR+cells from iγδTs (121-3 derivatives) were stimulated with HMBPP and IL-2 for 10 days from day 30, and sorted on BD FACS melody at day 40. PBγδTs were stimulated for two weeks and not sorted. Total RNA was extracted using TRIzol reagent and treated with a Turbo DNA-free kit. cDNA was synthesized and amplified for TCRγ or TCRδ genes with the ligation of a universal adaptor to the leader sequence of variable regions. Sequencing was performed using an illumina MiSeq. More than 1×10^5 total read sequences were analyzed at Repertoire Genesis (Osaka, Japan)

The amino acid sequence reads with a frequency of <1% were omitted from the subsequent analysis as this assay does not guarantee reliability for such low-frequency sequences.

Time-lapse imaging

GFP- Huh-7 cells were generated with piggyBac Mammalian Expression Vector containing CometGFP and puromycin-resistant gene (PJ509-02: ATUM). At day -1, tumor cells were seeded at density of 2×10^5 cells in a 35 mm dish. At day 0, 4×10^5 effector cells in 1 mL of γδT stimulating medium were added. Serial phase contrast images were captured at intervals with a microscope (Bio Studio: Nikon engineering).

Cytotoxic assay

Jurkat cells were collected into tubes and centrifuged at 300×g for 4 minutes. After PBS(-) washing, cells were suspended with buffer-added CFSE solution (Basic cytotoxicity: immunochemistry, #970) and incubated at room temperature for 15 min. We added FBS containing medium, centrifuged, PBS(-) washing was performed. Target cells were counted and suspended with fresh medium at density of 5.0×105 cells/mL. Target cell suspension (100 μL) was seeded onto a 96-well U-bottom plate and 100 μL of effector cell suspension in γδT stimulating medium was added at several concentrations. As a negative control, 100 μL of γδT stimulating medium without effector cells was added. After co-culturing at 37°C overnight, we added 7-AAD solution into the well and performed FCM.

The SW480 cells were incubated with CFSE for 15 min before co-culture. For adherent cells, at day -1,

tumor cells were seeded at density of 2×10^5 cells in a 35 mm dish. At day 0, 4×10^5 effector cells in 1 mL of γδT stimulating medium were added. They were assessed after 12 hours (Huh-7) or 16 hours (SW480) co-culture.

Serial phase contrast images were captured at intervals with a microscope (Bio Studio: Nikon engineering). The areas of tumor cells were quantified using the Image J software program (ImageJ ver 1.51k, NIH) and the areas of surviving cells with or without iγδTs were calculated after correcting with the value before co-culture (0 h) as follows:

$$\frac{\text{Area of tumor cells (12 h or 16 h, i}\gamma\delta\text{T)/Area of tumor cells (0 h, i}\gamma\delta\text{T)}}{\text{Area of tumor cells (12 h or 16 h, no effector)/Area of tumor cells (0 h, no effector)}}$$

= X%/100%

Real-time cytotoxicity assay (xCELLigence assay)

The cytolytic potential of isolated iγδT cells was analyzed in a real-time cytotoxicity assay with an xCELLigence RTCA system (Agilent, 380601030). Huh-7 cells (1×10^4) were seeded in each well of a 96-well E-Plate. After one day, iγδT, PBγδT or PBNK cells were added at an E:T ratio of 2:1. Cell viability was monitored every 15 min for 12 h. Cell indexes (CIs) were normalized to the CI of the time-point when effector cells were added and specific lysis was calculated in relation to the control cells lacking any effector cells. In the case of floating K562 cells, cells were seeded on pre-coated wells with an xCELLigence immunoassay kit anti-CD71 (Agilent, 8100017). If needed, the wells were added with 20 µg/mL anti-human TCRγδ Ab (clone B1.1, eBioscience) or 20 µg/mL anti-human NKG2D Ab (clone 1D11, eBioscience).

Flow cytometry, fluorescence-activated cell sorting

Cells were filtered using a 35 µm cell strainer and incubated with antibodies diluted in FACS buffer on 4°C for 30 minutes with light shielding. After washing twice with FACS buffer (Nacalai Tesque, 25976–14), cells were re-suspended in FACS buffer and filtered with a 35 µm cell strainer again, analyzed on RF-500 (Sysmex) or sorted on FACS Aria III (BD Biosciences) or FACS Melody (BD Biosciences). Antibodies were listed in supplemental table S5.

FCM of cytoplasmic molecules

Brefeldin A was supplemented in a medium at a final concentration of 3 µg/mL. Effector cells were incubated at 37°C for 30 minutes, and co-cultured with target cells for 6 hours in a condition of Brefeldin A-containing medium. Then, mixed cells were collected, subjected to PBS (-) washing, and fixed with 4% paraformaldehyde for 20 minutes. The cells were rewashed, then permeabilized, blocked and incubated with antibodies diluted in 0.3% TritonX-100 in 1% human serum for 30 minutes on ice with light shielding. After washing, the cells were analyzed on an RF-500 flow cytometer.

Magnetic-activated cell sorting

Before the cytotoxicity assay, iγδTs and PBγδTs were purified with MACS CD3-MicroBeads (Miltenyi Biotec, 130-050-101) according to the manufacturer's instructions. PBNK cells were collected using an EasySep Human NK Cell isolation Kit (STEMCELL technologies, 18000).

Human Subjects

Peripheral blood mononuclear cells were isolated from normal healthy donors using protocols approved by the Kobe University Institutional Review Board. Informed consent was obtained from all subjects in accordance with the IRB protocols.

SUPPLEMENTAL REFERENCES

Miyazaki, T., Isobe, T., Nakatsuji, N., and Suemori, H. (2017). Efficient Adhesion Culture of Human Pluripotent Stem Cells Using Laminin Fragments in an Uncoated Manner. Sci Rep *7*, 41165. DOI: 10.1038/srep41165

Watanabe, D., Koyanagi-Aoi, M., Taniguchi-Ikeda, M., Yoshida, Y., Azuma, T., and Aoi, T. (2018). The Generation of Human gammadeltaT Cell-Derived Induced Pluripotent Stem Cells from Whole Peripheral Blood Mononuclear Cell Culture. Stem Cells Transl Med *7*, 34-44. DOI: 10.1002/sctm.17-0021
